# Supplementary material for: Genomic Epidemiology Unveil the Omicron Transmission Dynamics in Rome, Italy
Source: Pathogens. 2022 Sep 4;11(9):1011. doi: 10.3390/pathogens11091011 (PMC9505927; doi:10.3390/pathogens11091011)
Supplement: Supplementary file 1 [file pathogens-11-01011-s001.zip › pathogens-1894226-supplementary.pdf]

Table S1. Complete genomes of the SARS-CoV2 Omicron variant.

Accession ID	Collection date	Lineage	Lin	Clade	AA Substitutions
EPI_ISL_9014138	21/12/2021	BA.1	BA.1.17.2	GRA	(NSP5_P132H,Spike_H69del,Spike_T95I,Spike_A67V,Spike_S373P,Spike_N969K,Spike_H655Y,Spike_N856K,N_R203K,Spike_G142D,NSP3_A1892T,Spike_Q954H,N_P13L,NSP3_L1266I,N_R32del,M_Q19E,Spike_N440K,NSP4_T492I,NSP6_L105del,Spike_N679K,Spike_N764K,Spike_L212I,NSP6_G107del,NSP6_I189V,Spike_T547K,M_D3G,Spike_D796Y,N_G204R,Spike_T478K,Spike_V143del,M_A63T,NSP3_V1069I,Spike_K417N,NSP6_S106del,Spike_S371L,Spike_G339D,NSP3_S1265del,NSP3_L1870F,NSP14_I42V,Spike_P681H,Spike_Y144del,Spike_ins214EPE,N_S33del,Spike_S375F,NSP1_F143del,Spike_G446S,NSP1_K141del,N_E31del,NSP3_K38R,Spike_N211del,NSP1_S142del,E_T9I,Spike_V70del,Spike_L981F,NSP12_P323L,Spike_Y145del,Spike_D614G)
EPI_ISL_9014182	21/12/2021	BA.1	BA.1	GRA	(NSP5_P132H,Spike_H69del,Spike_T95I,Spike_A67V,Spike_S373P,Spike_Q493R,Spike_N969K,Spike_H655Y,Spike_N856K,N_R203K,Spike_G142D,NSP3_A1892T,Spike_Q954H,N_P13L,NSP3_L1266I,Spike_N501Y,N_R32del,M_Q19E,Spike_N440K,NSP4_T492I,NSP6_L105del,Spike_N679K,Spike_N764K,Spike_L212I,NSP6_G107del,Spike_Y505H,NSP6_I189V,Spike_T547K,M_D3G,Spike_D796Y,N_G204R,Spike_V143del,M_A63T,Spike_G496S,Spike_K417N,NSP6_S106del,Spike_S371L,Spike_G339D,NSP3_S1265del,NSP14_I42V,Spike_P681H,Spike_Y144del,Spike_ins214EPE,N_S33del,Spike_S375F,Spike_Q498R,Spike_G446S,N_E31del,NSP3_K38R,Spike_N211del,Spike_E484A,E_T9I,Spike_V70del,Spike_L981F,NSP12_P323L,Spike_D614G,Spike_Y145del)
EPI_ISL_9014189	21/12/2021	BA.1	BA.1.17.2	GRA	(NSP5_P132H,Spike_H69del,Spike_T95I,Spike_A67V,Spike_S373P,Spike_Q493R,Spike_N969K,Spike_H655Y,Spike_N856K,N_R203K,Spike_G142D,NSP3_A1892T,Spike_Q954H,Spike_A701V,N_P13L,NSP3_L1266I,Spike_N501Y,N_R32del,M_Q19E,Spike_N440K,NSP4_T492I,NSP6_L105del,Spike_N679K,Spike_N764K,Spike_L212I,NSP6_G107del,Spike_Y505H,NSP6_I189V,Spike_T547K,M_D3G,Spike_D796Y,N_G204R,Spike_V143del,M_A63T,Spike_G496S,NSP3_V1069I,Spike_K417N,NSP6_S106del,Spike_S371L,Spike_G339D,NSP3_S1265del,NSP3_L1870F,NSP14_I42V,Spike_P681H,Spike_Y144del,Spike_ins214EPE,N_S33del,Spike_S375F,NSP1_F143del,Spike_Q498R,Spike_G446S,NSP1_K141del,N_E31del,NSP3_K38R,Spike_N211del,NSP1_S142del,Spike_E484A,E_T9I,Spike_V70del,Spike_L981F,NSP12_P323L,Spike_D614G,Spike_Y145del)
EPI_ISL_9014201	21/12/2021	BA.1	BA.1.17.2	GRA	(NSP5_P132H,Spike_H69del,Spike_T95I,Spike_A67V,Spike_S373P,Spike_N969K,Spike_H655Y,Spike_N856K,N_R203K,Spike_G142D,NSP3_A1892T,Spike_Q954H,Spike_A701V,N_P13L,NSP3_L1266I,N_R32del,M_Q19E,Spike_N440K,NSP4_T492I,NSP6_L105del,Spike_N679K,Spike_N764K,Spike_L212I,NSP6_G107del,NSP6_I189V,Spike_T547K,M_D3G,Spike_D796Y,N_G204R,Spike_V143del,M_A63T,NSP3_V1069I,Spike_K417N,NSP6_S106del,Spike_S371L,Spike_G339D,NSP3_S1265del,NSP14_I42V,Spike_P681H,Spike_Y144del,Spike_ins214EPE,N_S33del,Spike_S375F,Spike_G446S,N_E31del,NSP3_K38R,Spike_N211del,E_T9I,Spike_V70del,Spike_L981F,NSP12_P323L,Spike_Y145del,Spike_D614G)
EPI_ISL_9014218	21/12/2021	BA.1	BA.1	GRA	(NSP5_P132H,N_G215C,Spike_T95I,Spike_S373P,Spike_N969K,Spike_N856K,N_R203K,Spike_G142D,NSP3_A1892T,N_P13L,Spike_N501Y,N_R32del,M_Q19E,NSP4_T492I,Spike_T19R,NSP6_L105del,Spike_D950N,Spike_N764K,Spike_L212I,Spike_Y505H,NSP6_G107del,Spike_T547K,M_D3G,Spike_D796Y,N_G204R,Spike_T478K,Spike_V143del,M_A63T,Spike_G496S,Spike_K417N,NSP6_S106del,Spike_S371L,Spike_G339D,Spike_R158del,Spike_Y144del,Spike_ins214EPE,Spike_F157del,N_S33del,Spike_S375F,Spike_Q498R,NSP13_P77L,N_E31del,Spike_E156G,Spike_N211del,Spike_L981F,NSP12_P323L,Spike_D614G,Spike_Y145del,Spike_L452R,NSP12_L749M)
EPI_ISL_9012399	17/01/2022	BA.1	BA.1.1	GRA	(NSP5_P132H,Spike_H69del,Spike_T95I,Spike_A67V,Spike_S373P,Spike_Q493R,Spike_N969K,Spike_H655Y,Spike_N856K,N_R203K,Spike_G142D,NSP3_A1892T,Spike_Q954H,N_P13L,NSP3_L1266I,Spike_N501Y,N_R32del,M_Q19E,Spike_N440K,NSP4_T492I,NSP6_L105del,Spike_N679K,Spike_N764K,Spike_L212I,NSP6_G107del,Spike_Y505H,Spike_R346K,NSP6_I189V,N_G204L,Spike_T547K,M_D3G,Spike_D796Y,Spike_V143del,M_A63T,Spike_G496S,Spike_K417N,NSP6_S106del,Spike_S371L,Spike_G339D,NSP3_S1265del,NSP14_I42V,Spike_P681H,Spike_Y144del,Spike_ins214EPE,N_S33del,Spike_S375F,Spike_Q498R,Spike_G446S,N_E31del,NSP3_K38R,Spike_N211del,Spike_E484A,E_T9I,Spike_V70del,Spike_L981F,NSP12_P323L,Spike_D614G,Spike_Y145del)
EPI_ISL_9012400	17/01/2022	BA.1	BA.1.1	GRA	(NSP5_P132H,Spike_H69del,Spike_T95I,Spike_A67V,Spike_S373P,Spike_N969K,Spike_H655Y,Spike_N856K,N_R203K,Spike_G142D,NSP3_A1892T,Spike_Q954H,N_P13L,NSP3_L1266I,N_R32del,M_Q19E,Spike_N440K,NSP4_T492I,NSP6_L105del,Spike_N679K,Spike_N764K,Spike_L212I,NSP6_G107del,Spike_R346K,NSP6_I189V,N_G204L,Spike_T547K,M_D3G,Spike_D796Y,Spike_V143del,M_A63T,Spike_K417N,NSP6_S106del,Spike_S371L,Spike_G339D,NSP3_S1265del,NSP14_I42V,Spike_P681H,Spike_Y144del,Spike_ins214EPE,N_S33del,Spike_S375F,Spike_G446S,N_E31del,NSP3_K38R,Spike_N211del,E_T9I,Spike_V70del,Spike_L981F,NSP12_P323L,Spike_Y145del,Spike_D614G)
EPI_ISL_9012401	17/01/2022	BA.1	BA.1.17.2	GRA	(NSP5_P132H,Spike_H69del,Spike_T95I,Spike_A67V,Spike_S373P,Spike_N969K,Spike_H655Y,Spike_N856K,N_R203K,Spike_G142D,NSP3_A1892T,Spike_Q954H,Spike_A701V,N_P13L,NSP3_L1266I,N_R32del,M_Q19E,Spike_N440K,NSP4_T492I,NSP6_L105del,Spike_N679K,Spike_N764K,Spike_L212I,NSP6_G107del,NSP6_I189V,Spike_T547K,M_D3G,Spike_D796Y,N_G204R,Spike_V143del,M_A63T,NS7a_S98F,NSP3_V1069I,Spike_K417N,NSP6_S106del,Spike_S371L,Spike_G339D,NSP3_S1265del,NSP14_I42V,Spike_P681H,Spike_Y144del,Spike_ins214EPE,N_S33del,Spike_S375F,Spike_G446S,N_E31del,NSP3_K38R,Spike_N211del,E_T9I,Spike_V70del,Spike_L981F,NSP12_P323L,Spike_Y145del,Spike_D614G)
EPI_ISL_9012403	17/01/2022	BA.1	BA.1.1	GRA	(NSP5_P132H,Spike_H69del,Spike_T95I,Spike_A67V,Spike_S373P,Spike_N969K,Spike_H655Y,Spike_N856K,N_R203K,Spike_G142D,NSP3_A1892T,Spike_Q954H,N_P13L,NSP3_L1266I,N_R32del,M_Q19E,Spike_N440K,NSP4_T492I,NSP6_L105del,Spike_N679K,Spike_N764K,Spike_L212I,NSP6_G107del,Spike_R346K,NSP6_I189V,Spike_T547K,M_D3G,Spike_D796Y,N_G204R,Spike_V143del,M_A63T,Spike_K417N,NSP6_S106del,Spike_S371L,Spike_G339D,NSP3_S1265del,NSP14_I42V,Spike_P681H,Spike_Y144del,Spike_ins214EPE,N_S33del,Spike_S375F,Spike_G446S,N_E31del,NSP3_K38R,Spike_N211del,E_T9I,Spike_V70del,Spike_L981F,NSP12_P323L,Spike_Y145del,Spike_D614G)
EPI_ISL_9012404	17/01/2022	BA.1	BA.1.21.1	GRA	(NSP5_P132H,Spike_H69del,Spike_T95I,Spike_A67V,NS7b_E3stop,Spike_S373P,Spike_N969K,Spike_H655Y,Spike_N856K,N_R203K,Spike_G142D,NSP3_A1892T,Spike_Q954H,N_P13L,NSP3_L1266I,N_R32del,M_Q19E,Spike_N440K,NSP4_T492I,NSP6_L105del,Spike_N679K,Spike_N764K,Spike_L212I,NSP6_G107del,NSP6_I189V,Spike_T547K,M_D3G,Spike_D796Y,N_G204R,Spike_V143del,M_A63T,Spike_K417N,NSP6_S106del,Spike_S371L,Spike_G339D,NSP3_S1265del,NSP14_I42V,Spike_P681H,Spike_Y144del,Spike_ins214EPE,N_S33del,Spike_S375F,Spike_G446S,N_E31del,NSP3_K38R,Spike_N211del,E_T9I,Spike_V70del,Spike_L981F,NSP12_P323L,Spike_Y145del,Spike_D614G,NSP12_L749M)

EPI_ISL_9012405	17/01/2022	BA.1	BA.1.17.2	GRA	(NSP5_P132H,Spike_H69del,Spike_T95I,Spike_A67V,Spike_S373P,Spike_N969K,Spike_H655Y,Spike_N856K,N_R203K,Spike_G142D,NSP3_A1892T,Spike_Q954H,Spike_A701V,N_P13L,NSP3_L1266I,N_R32del,M_Q19E,Spike_N440K,NSP4_T492I,NSP6_L105del,Spike_N679K,Spike_N764K,Spike_L212I,NSP6_G107del,NSP6_I189V,Spike_T547K,M_D3G,Spike_D796Y,N_G204R,Spike_V143del,M_A63T,NSP3_V1069I,Spike_K417N,NSP6_S106del,Spike_S371L,Spike_G339D,NSP3_S1265del,NSP14_I42V,Spike_P681H,Spike_Y144del,Spike_ins214EPE,N_S33del,Spike_S375F,Spike_G446S,N_E31del,NSP3_K38R,Spike_N211del,E_T9I,Spike_V70del,Spike_L981F,NSP12_P323L,Spike_Y145del,Spike_D614G)
EPI_ISL_9012406	17/01/2022	BA.1	BA.1.1.1	GRA	(NSP5_P132H,Spike_H69del,Spike_T95I,Spike_A67V,Spike_S373P,Spike_Q493R,Spike_N969K,Spike_H655Y,Spike_N856K,N_R203K,Spike_G142D,NSP3_A1892T,Spike_Q954H,N_P13L,NSP3_L1266I,Spike_N501Y,N_R32del,M_Q19E,Spike_N440K,NSP4_T492I,NSP6_L105del,Spike_N679K,Spike_N764K,Spike_L212I,NSP6_G107del,Spike_Y505H,Spike_R346K,NSP6_I189V,Spike_T547K,M_D3G,Spike_D796Y,N_G204R,Spike_V143del,M_A63T,Spike_G496S,NSP12_Q875R,Spike_K417N,NSP6_S106del,Spike_S371L,Spike_G339D,NSP3_S1265del,NSP14_I42V,Spike_P681H,Spike_Y144del,Spike_ins214EPE,N_S33del,Spike_S375F,Spike_Q498R,Spike_G446S,N_E31del,NSP3_K38R,Spike_N211del,Spike_E484A,E_T9I,Spike_V70del,Spike_L981F,NSP12_P323L,Spike_D614G,Spike_Y145del)
EPI_ISL_9012454	17/01/2022	BA.1	BA.1.17.2	GRA	(NSP5_P132H,Spike_H69del,Spike_T95I,Spike_A67V,Spike_S373P,Spike_N969K,Spike_H655Y,Spike_N856K,N_R203K,Spike_G142D,NSP3_A1892T,Spike_Q954H,Spike_A701V,N_P13L,NSP3_L1266I,N_R32del,M_Q19E,Spike_N440K,NSP4_T492I,NSP6_L105del,Spike_N679K,Spike_N764K,Spike_L212I,NSP6_G107del,NSP6_I189V,Spike_T547K,M_D3G,Spike_D796Y,N_G204R,Spike_V143del,M_A63T,NSP3_V1069I,Spike_K417N,NSP6_S106del,Spike_S371L,Spike_G339D,NSP3_S1265del,NSP14_I42V,Spike_P681H,Spike_Y144del,Spike_ins214EPE,N_S33del,Spike_S375F,Spike_G446S,N_E31del,NSP3_K38R,Spike_N211del,E_T9I,Spike_V70del,Spike_L981F,NSP12_P323L,Spike_Y145del,Spike_D614G)
EPI_ISL_9012455	17/01/2022	BA.1	BA.1.17	GRA	(NSP5_P132H,Spike_H69del,Spike_T95I,Spike_A67V,Spike_S373P,Spike_N969K,Spike_H655Y,Spike_N856K,N_R203K,Spike_G142D,NSP3_A1892T,Spike_Q954H,N_P13L,NSP3_L1266I,N_R32del,M_Q19E,Spike_N440K,NSP4_T492I,NSP6_L105del,Spike_N679K,Spike_N764K,Spike_L212I,NSP6_G107del,NSP6_I189V,Spike_T547K,M_D3G,Spike_D796Y,N_G204R,Spike_V143del,M_A63T,NSP3_P985S,NSP3_V1069I,Spike_K417N,NSP6_S106del,Spike_S371L,Spike_G339D,NSP3_S1265del,NSP14_I42V,Spike_P681H,Spike_Y144del,Spike_ins214EPE,N_S33del,Spike_S375F,Spike_G446S,N_E31del,NSP3_K38R,Spike_N211del,E_T9I,Spike_V70del,Spike_L981F,NSP12_P323L,Spike_Y145del,Spike_D614G)
EPI_ISL_9012456	17/01/2022	BA.1	BA.1.17.2	GRA	(NSP5_P132H,Spike_H69del,Spike_T95I,Spike_A67V,Spike_S373P,Spike_Q493R,Spike_N969K,Spike_H655Y,Spike_N856K,N_R203K,Spike_G142D,NSP3_A1892T,Spike_Q954H,Spike_A701V,N_P13L,NSP3_L1266I,N_R32del,M_Q19E,Spike_N440K,NSP4_T492I,NSP6_L105del,Spike_N679K,Spike_N764K,Spike_L212I,NSP6_G107del,NSP6_I189V,Spike_T547K,M_D3G,Spike_D796Y,N_G204R,Spike_V143del,M_A63T,NSP3_V1069I,Spike_K417N,NSP6_S106del,Spike_S371L,Spike_G339D,NSP3_S1265del,NSP14_I42V,Spike_P681H,Spike_Y144del,Spike_ins214EPE,N_S33del,Spike_S375F,Spike_G446S,N_E31del,NSP3_K38R,Spike_N211del,Spike_E484A,E_T9I,Spike_V70del,Spike_L981F,NSP12_P323L,Spike_Y145del,Spike_D614G)
EPI_ISL_9012458	17/01/2022	BA.1	BA.1	GRA	(NSP5_P132H,Spike_H69del,Spike_T95I,Spike_A67V,Spike_S373P,Spike_Q493R,Spike_N969K,Spike_H655Y,Spike_N856K,N_R203K,Spike_G142D,NSP3_A1892T,Spike_Q954H,N_P13L,NSP3_L1266I,Spike_N501Y,N_R32del,M_Q19E,Spike_N440K,NSP4_T492I,NSP6_L105del,Spike_N679K,Spike_N764K,Spike_L212I,NSP6_G107del,Spike_Y505H,NSP6_I189V,Spike_T547K,M_D3G,Spike_D796Y,N_G204R,Spike_V143del,M_A63T,Spike_G496S,Spike_K417N,NSP6_S106del,Spike_S371L,Spike_G339D,NSP3_S1265del,NSP14_I42V,Spike_P681H,Spike_Y144del,Spike_ins214EPE,N_S33del,Spike_S375F,Spike_Q498R,Spike_G446S,N_E31del,NSP3_K38R,Spike_N211del,Spike_E484A,E_T9I,Spike_V70del,Spike_L981F,NSP12_P323L,Spike_D614G,Spike_Y145del)
EPI_ISL_9012459	17/01/2022	BA.1	BA.1.1	GRA	(NSP5_P132H,Spike_H69del,Spike_T95I,Spike_A67V,Spike_S373P,Spike_N969K,Spike_H655Y,Spike_N856K,N_R203K,Spike_G142D,NSP3_A1892T,Spike_Q954H,N_P13L,NSP3_L1266I,N_R32del,M_Q19E,Spike_N440K,NSP4_T492I,NSP6_L105del,Spike_N679K,Spike_N764K,Spike_L212I,NSP6_G107del,Spike_R346K,NSP6_I189V,Spike_T547K,M_D3G,Spike_D796Y,N_G204R,Spike_V143del,M_A63T,Spike_K417N,NSP6_S106del,Spike_S371L,Spike_G339D,NSP3_S1265del,NSP14_I42V,Spike_P681H,Spike_Y144del,Spike_ins214EPE,N_S33del,Spike_S375F,Spike_G446S,N_E31del,NSP3_K38R,Spike_N211del,E_T9I,Spike_V70del,Spike_L981F,NSP12_P323L,Spike_Y145del,Spike_D614G)
EPI_ISL_9012460	17/01/2022	BA.1	BA.1.1	GRA	(NSP5_P132H,Spike_H69del,Spike_T95I,Spike_A67V,Spike_S373P,Spike_N969K,Spike_H655Y,Spike_N856K,N_R203K,Spike_G142D,NSP3_A1892T,Spike_Q954H,N_P13L,NSP3_L1266I,N_R32del,M_Q19E,Spike_N440K,NSP4_T492I,NSP6_L105del,Spike_N679K,Spike_N764K,Spike_L212I,NSP6_G107del,Spike_R346K,NSP6_I189V,Spike_T547K,M_D3G,Spike_D796Y,N_G204R,Spike_V143del,M_A63T,Spike_G496S,Spike_K417N,NSP6_S106del,Spike_S371L,Spike_G339D,NSP3_S1265del,NSP14_I42V,Spike_P681H,Spike_Y144del,Spike_ins214EPE,N_S33del,Spike_S375F,Spike_G446S,N_E31del,NSP3_K38R,Spike_N211del,Spike_E484A,E_T9I,Spike_V70del,Spike_L981F,NSP12_P323L,Spike_D614G,Spike_Y145del)
EPI_ISL_9012461	17/01/2022	BA.1	BA.1.17	GRA	(NSP5_P132H,Spike_H69del,Spike_T95I,Spike_A67V,Spike_S373P,Spike_N969K,Spike_H655Y,Spike_N856K,N_R203K,Spike_G142D,NSP3_A1892T,Spike_Q954H,N_P13L,NSP3_L1266I,N_R32del,M_Q19E,Spike_N440K,NSP4_T492I,NSP6_L105del,Spike_N679K,Spike_N764K,Spike_L212I,NSP6_G107del,NSP6_I189V,Spike_T547K,M_D3G,Spike_D796Y,NS3_A103T,N_G204R,Spike_V143del,M_A63T,NSP3_P985S,NSP13_R129K,NSP3_V1069I,Spike_K417N,NSP6_S106del,Spike_S371L,Spike_G339D,NSP3_S1265del,NSP14_I42V,Spike_P681H,Spike_Y144del,Spike_ins214EPE,N_S33del,Spike_S375F,Spike_G446S,N_E31del,NSP3_K38R,Spike_N211del,E_T9I,Spike_V70del,Spike_L981F,NSP12_P323L,Spike_Y145del,Spike_D614G)
EPI_ISL_9012573	17/01/2022	BA.1	BA.1	GRA	(NSP5_P132H,Spike_H69del,Spike_T95I,Spike_A67V,Spike_S373P,Spike_N969K,Spike_H655Y,Spike_N856K,N_R203K,Spike_G142D,NSP3_A1892T,Spike_Q954H,N_P13L,NSP3_L1266I,N_R32del,M_Q19E,Spike_N440K,NSP4_T492I,NSP6_L105del,Spike_N679K,Spike_N764K,Spike_L212I,NSP6_G107del,NSP6_I189V,Spike_T547K,M_D3G,Spike_D796Y,N_G204R,Spike_V143del,M_A63T,Spike_K417N,NSP6_S106del,Spike_S371L,Spike_G339D,NSP3_S1265del,NSP14_I42V,Spike_P681H,Spike_Y144del,Spike_ins214EPE,N_S33del,Spike_S375F,Spike_G446S,N_E31del,NSP3_K38R,Spike_N211del,E_T9I,Spike_V70del,Spike_L981F,NSP12_P323L,Spike_Y145del,Spike_D614G)
EPI_ISL_9012579	17/01/2022	BA.1	BA.1.15	GRA	(NSP5_P132H,Spike_H69del,Spike_T95I,Spike_A67V,Spike_S373P,Spike_Q493R,Spike_N969K,Spike_H655Y,Spike_N856K,N_R203K,Spike_G142D,NSP3_A1892T,Spike_Q954H,NS3_L106F,N_P13L,NSP3_L1266I,Spike_N501Y,N_R32del,M_Q19E,Spike_N440K,NSP4_T492I,NSP6_L105del,Spike_N679K,Spike_N764K,Spike_L212I,NSP6_G107del,Spike_Y505H,NSP6_I189V,Spike_T547K,M_D3G,Spike_D796Y,N_G204R,N_D343G,Spike_V143del,M_A63T,Spike_G496S,Spike_K417N,NSP6_S106del,Spike_S371L,Spike_G339D,NSP3_S1265del,NSP14_I42V,Spike_P681H,Spike_Y144del,Spike_ins214EPE,N_S33del,Spike_S375F,Spike_Q498R,Spike_G446S,N_E31del,NSP3_K38R,Spike_N211del,Spike_E484A,E_T9I,Spike_V70del,Spike_L981F,NSP12_P323L,Spike_D614G,Spike_Y145del)

EPI_ISL_9012617	17/01/2022	BA.1	BA.1	GRA	(NSP5_P132H,Spike_H69del,Spike_T95I,Spike_A67V,Spike_S373P,Spike_N969K,Spike_H655Y,Spike_N856K,N_R203K,Spike_G142D,NSP3_A1892T,Spike_Q954H,N_P13L,NSP3_L1266I,N_R32del,M_Q19E,Spike_N440K,NSP4_T492I,NSP6_L105del,Spike_N679K,Spike_N764K,Spike_L212I,NSP6_G107del,NSP6_I189V,Spike_T547K,M_D3G,Spike_D796Y,N_G204R,Spike_V143del,M_A63T,Spike_K417N,NSP6_S106del,Spike_S371L,Spike_G339D,NSP3_S1265del,NSP14_I42V,Spike_P681H,Spike_Y144del,Spike_ins214EPE,N_S33del,Spike_S375F,Spike_G446S,N_E31del,NSP3_K38R,Spike_N211del,E_T9I,Spike_V70del,Spike_L981F,NSP12_P323L,Spike_Y145del,Spike_D614G)
EPI_ISL_9012619	17/01/2022	BA.1	BA.1	GRA	(NSP5_P132H,Spike_H69del,Spike_T95I,Spike_A67V,Spike_S373P,Spike_Q493R,Spike_N969K,Spike_H655Y,Spike_N856K,N_R203K,Spike_G142D,NSP3_A1892T,Spike_Q954H,N_P13L,NSP3_L1266I,Spike_N501Y,N_R32del,M_Q19E,Spike_N440K,NSP4_T492I,NSP6_L105del,Spike_N679K,Spike_N764K,Spike_L212I,NSP6_G107del,Spike_Y505H,NSP6_I189V,Spike_T547K,M_D3G,Spike_D796Y,N_G204R,Spike_V143del,M_A63T,Spike_G496S,Spike_K417N,NSP6_S106del,Spike_S371L,Spike_G339D,NSP3_S1265del,NSP14_I42V,Spike_P681H,Spike_Y144del,Spike_ins214EPE,N_S33del,Spike_S375F,Spike_Q498R,Spike_G446S,N_E31del,NSP3_K38R,Spike_N211del,Spike_E484A,E_T9I,Spike_V70del,Spike_L981F,NSP12_P323L,Spike_D614G,Spike_Y145del)
EPI_ISL_9012620	17/01/2022	BA.1	BA.1	GRA	(NSP5_P132H,Spike_H69del,Spike_T95I,Spike_A67V,Spike_S373P,Spike_Q493R,Spike_N969K,Spike_H655Y,Spike_N856K,N_R203K,Spike_G142D,NSP3_A1892T,Spike_Q954H,N_P13L,NSP3_L1266I,Spike_N501Y,N_R32del,M_Q19E,Spike_N440K,NSP4_T492I,NSP6_L105del,Spike_N679K,Spike_N764K,Spike_L212I,NSP6_G107del,Spike_Y505H,NSP6_I189V,Spike_T547K,M_D3G,Spike_D796Y,N_G204R,Spike_V143del,M_A63T,Spike_G496S,Spike_K417N,NSP6_S106del,Spike_S371L,Spike_G339D,NSP3_S1265del,NSP14_I42V,Spike_P681H,Spike_Y144del,Spike_ins214EPE,N_S33del,Spike_S375F,Spike_Q498R,Spike_G446S,N_E31del,NSP3_K38R,Spike_N211del,Spike_E484A,E_T9I,Spike_V70del,Spike_L981F,NSP12_P323L,Spike_D614G,Spike_Y145del)
EPI_ISL_9012621	17/01/2022	BA.1	BA.1.18	GRA	(NSP5_P132H,Spike_H69del,Spike_T95I,Spike_A67V,Spike_S373P,Spike_N969K,Spike_H655Y,Spike_N856K,N_R203K,Spike_G142D,NSP3_A1892T,Spike_Q954H,NS3_D155Y,N_P13L,NS3_Y264H,NSP3_L1266I,N_R32del,M_Q19E,Spike_N440K,NSP4_T492I,NSP6_L105del,Spike_N679K,Spike_N764K,Spike_L212I,NSP6_G107del,NSP6_I189V,Spike_T547K,M_D3G,Spike_D796Y,N_G204R,Spike_V143del,M_A63T,Spike_K417N,NSP6_S106del,Spike_S371L,Spike_G339D,NSP3_S1265del,NSP14_I42V,Spike_P681H,Spike_Y144del,Spike_ins214EPE,N_S33del,Spike_S375F,Spike_G446S,NSP3_T1004I,N_E31del,NSP3_K38R,Spike_N211del,E_T9I,Spike_V70del,Spike_L981F,NSP12_P323L,Spike_Y145del,Spike_D614G)
EPI_ISL_9012622	17/01/2022	BA.1	BA.1	GRA	(NSP5_P132H,Spike_H69del,Spike_T95I,Spike_A67V,Spike_S373P,Spike_N969K,Spike_H655Y,Spike_N856K,N_R203K,Spike_G142D,NSP3_A1892T,Spike_Q954H,N_P13L,NSP3_L1266I,N_R32del,M_Q19E,Spike_N440K,NSP4_T492I,NSP6_L105del,Spike_N679K,Spike_N764K,Spike_L212I,NSP6_G107del,NSP6_I189V,Spike_T547K,M_D3G,Spike_D796Y,N_G204R,Spike_V143del,M_A63T,Spike_K417N,NSP6_S106del,Spike_S371L,Spike_G339D,NSP3_S1265del,NSP14_I42V,Spike_P681H,Spike_Y144del,Spike_ins214EPE,N_S33del,Spike_S375F,Spike_G446S,N_E31del,NSP3_K38R,Spike_N211del,E_T9I,Spike_V70del,Spike_L981F,NSP12_P323L,Spike_Y145del,Spike_D614G)
EPI_ISL_9012623	17/01/2022	BA.1	BA.1	GRA	(NSP5_P132H,Spike_H69del,Spike_T95I,Spike_A67V,Spike_S373P,Spike_N969K,Spike_H655Y,Spike_N856K,N_R203K,Spike_G142D,NSP3_A1892T,Spike_Q954H,N_P13L,NSP3_L1266I,N_R32del,M_Q19E,Spike_N440K,NSP4_T492I,NSP6_L105del,Spike_N679K,Spike_N764K,Spike_L212I,NSP6_G107del,NSP6_I189V,Spike_T547K,M_D3G,Spike_D796Y,N_G204R,Spike_V143del,M_A63T,Spike_K417N,NSP6_S106del,Spike_S371L,Spike_G339D,NSP3_S1265del,NSP14_I42V,Spike_P681H,Spike_Y144del,Spike_ins214EPE,N_S33del,Spike_S375F,Spike_G446S,N_E31del,NSP3_K38R,Spike_N211del,E_T9I,Spike_V70del,Spike_L981F,NSP12_P323L,Spike_Y145del,Spike_D614G)
EPI_ISL_9012624	17/01/2022	BA.1	BA.1.1	GRA	(NSP5_P132H,Spike_H69del,NSP2_T590I,Spike_T95I,Spike_A67V,Spike_S373P,Spike_Q493R,Spike_N969K,Spike_H655Y,Spike_N856K,N_R203K,Spike_G142D,NSP3_A1892T,Spike_Q954H,N_P13L,NSP3_L1266I,Spike_N501Y,N_R32del,M_Q19E,Spike_N440K,NSP4_T492I,NSP6_L105del,Spike_N679K,Spike_N764K,Spike_L212I,NSP6_G107del,Spike_Y505H,Spike_R346K,NSP6_I189V,Spike_T547K,M_D3G,Spike_D796Y,N_G204R,Spike_V143del,M_A63T,Spike_G496S,Spike_K417N,NSP6_S106del,Spike_S371L,Spike_G339D,NSP3_S1265del,NSP14_I42V,Spike_P681H,Spike_Y144del,Spike_ins214EPE,N_S33del,Spike_S375F,Spike_Q498R,Spike_G446S,N_E31del,NSP3_K38R,Spike_N211del,Spike_E484A,E_T9I,Spike_V70del,Spike_L981F,NS3_E241G,NSP12_P323L,Spike_D614G,Spike_Y145del)
EPI_ISL_9012625	17/01/2022	BA.1	BA.1.1	GRA	(NSP5_P132H,Spike_H69del,Spike_T95I,Spike_A67V,Spike_S373P,Spike_N969K,Spike_H655Y,Spike_N856K,N_R203K,Spike_G142D,NSP3_A1892T,Spike_Q954H,N_P13L,NSP3_L1266I,N_R32del,M_Q19E,Spike_N440K,NSP4_T492I,NSP6_L105del,Spike_N679K,Spike_N764K,Spike_L212I,NSP6_G107del,Spike_R346K,NSP6_I189V,Spike_T547K,M_D3G,Spike_D796Y,N_G204R,Spike_V143del,M_A63T,Spike_K417N,NSP6_S106del,Spike_S371L,Spike_G339D,NSP3_S1265del,NSP14_I42V,Spike_P681H,Spike_Y144del,Spike_ins214EPE,N_S33del,Spike_S375F,Spike_G446S,N_E31del,NSP3_K38R,Spike_N211del,E_T9I,Spike_V70del,Spike_L981F,NSP12_P323L,Spike_Y145del,Spike_D614G)
EPI_ISL_9012626	17/01/2022	BA.1	BA.1.17	GRA	(NSP5_P132H,Spike_H69del,Spike_T95I,Spike_A67V,Spike_S373P,Spike_N969K,Spike_H655Y,Spike_N856K,N_R203K,Spike_G142D,NSP3_A1892T,Spike_Q954H,N_P13L,NSP3_L1266I,N_R32del,M_Q19E,Spike_N440K,NSP4_T492I,NSP6_L105del,Spike_N679K,Spike_N764K,Spike_L212I,NSP6_G107del,NSP6_I189V,Spike_T547K,M_D3G,Spike_D796Y,N_G204R,Spike_V143del,M_A63T,NSP3_V1069I,Spike_K417N,NSP6_S106del,Spike_S371L,Spike_G339D,NSP3_S1265del,NSP14_I42V,Spike_P681H,Spike_Y144del,Spike_ins214EPE,N_S33del,Spike_S375F,Spike_G446S,N_E31del,NSP3_K38R,Spike_N211del,E_T9I,Spike_V70del,Spike_L981F,NSP12_P323L,Spike_Y145del,Spike_D614G)
EPI_ISL_9012686	17/01/2022	BA.1	BA.1.1	GRA	(NSP5_P132H,Spike_H69del,Spike_T95I,Spike_A67V,Spike_S373P,Spike_N969K,Spike_H655Y,Spike_N856K,N_R203K,Spike_G142D,NSP3_A1892T,Spike_Q954H,N_P13L,NSP3_L1266I,N_R32del,M_Q19E,Spike_N440K,NSP4_T492I,NSP6_L105del,Spike_N679K,Spike_N764K,Spike_L212I,NSP6_G107del,Spike_R346K,NSP6_I189V,Spike_T547K,M_D3G,Spike_D796Y,N_G204R,Spike_V143del,M_A63T,Spike_K417N,NS8_I121L,NSP6_S106del,Spike_S371L,Spike_G339D,NSP3_S1265del,NSP14_I42V,Spike_P681H,Spike_Y144del,Spike_ins214EPE,N_S33del,Spike_S375F,Spike_G446S,NS8_F120V,N_E31del,NSP3_K38R,Spike_N211del,E_T9I,Spike_V70del,Spike_L981F,NSP12_P323L,Spike_Y145del,Spike_D614G)
EPI_ISL_9012687	17/01/2022	BA.1	BA.1.17.2	GRA	(NSP5_P132H,Spike_H69del,Spike_T95I,Spike_A67V,Spike_S373P,Spike_N969K,Spike_H655Y,Spike_N856K,N_R203K,Spike_G142D,NSP3_A1892T,Spike_Q954H,Spike_A701V,N_P13L,NSP3_L1266I,N_R32del,M_Q19E,Spike_N440K,NSP4_T492I,NSP6_L105del,Spike_N679K,Spike_N764K,Spike_L212I,NSP6_G107del,NSP6_I189V,Spike_T547K,M_D3G,Spike_D796Y,N_G204R,Spike_V143del,M_A63T,NSP3_L672F,NSP3_V1069I,Spike_K417N,NSP6_S106del,Spike_S371L,Spike_G339D,NSP3_S1265del,NSP14_I42V,Spike_P681H,NSP2_L270I,Spike_Y144del,Spike_ins214EPE,N_S33del,Spike_S375F,Spike_G446S,N_E31del,NSP3_K38R,Spike_N211del,NSP3_E229K,E_T9I,Spike_V70del,Spike_L981F,NSP12_P323L,Spike_Y145del,Spike_D614G)

EPI_ISL_9012688	17/01/2022	BA.1	BA.1.17.2	GRA	(NSP5_P132H,Spike_H69del,Spike_T95I,Spike_A67V,NSP3_A1311V,Spike_S373P,Spike_N969K,Spike_H655Y,Spike_N856K,N_R203K,Spike_G142D,NSP3_A1892T,Spike_Q954H,Spike_A701V,N_P13L,NSP3_L1266I,N_R32del,M_Q19E,Spike_N440K,NSP4_T492I,NSP6_L105del,Spike_N679K,Spike_N764K,Spike_L212I,NSP6_G107del,NSP6_I189V,Spike_T547K,M_D3G,Spike_D796Y,N_G204R,Spike_V143del,M_A63T,NSP3_V1069I,Spike_K417N,NSP6_S106del,Spike_S371L,Spike_G339D,NSP3_S1265del,NSP14_I42V,Spike_P681H,Spike_Y144del,Spike_ins214EPE,N_S33del,Spike_S375F,Spike_G446S,N_E31del,NSP3_K38R,Spike_N211del,E_T9I,Spike_V70del,Spike_L981F,NSP12_P323L,Spike_Y145del,Spike_D614G)
EPI_ISL_9012693	17/01/2022	BA.1	BA.1.17.2	GRA	(NSP5_P132H,Spike_H69del,Spike_T95I,Spike_A67V,Spike_S373P,Spike_N969K,Spike_H655Y,Spike_N856K,N_R203K,Spike_G142D,NSP3_A1892T,Spike_Q954H,Spike_A701V,N_P13L,NSP3_L1266I,N_R32del,M_Q19E,Spike_N440K,NSP4_T492I,NSP6_L105del,Spike_N679K,Spike_N764K,Spike_L212I,NSP6_G107del,NSP6_I189V,Spike_T547K,M_D3G,Spike_D796Y,N_G204R,Spike_V143del,M_A63T,NSP3_V1069I,Spike_K417N,NSP6_S106del,Spike_S371L,Spike_G339D,NSP3_S1265del,NSP5_L253F,NSP14_I42V,Spike_P681H,Spike_Y144del,Spike_ins214EPE,N_S33del,Spike_S375F,Spike_G446S,N_E31del,NSP3_K38R,Spike_N211del,E_T9I,Spike_V70del,Spike_L981F,NSP12_P323L,Spike_Y145del,Spike_D614G)
EPI_ISL_9012695	17/01/2022	BA.1	BA.1.1	GRA	(NSP5_P132H,Spike_H69del,Spike_T95I,Spike_A67V,Spike_S373P,Spike_Q493R,Spike_N969K,Spike_H655Y,Spike_N856K,N_R203K,Spike_G142D,NSP3_A1892T,Spike_Q954H,N_P13L,NSP3_L1266I,N_R32del,M_Q19E,Spike_N440K,NSP4_T492I,NSP6_L105del,Spike_N679K,Spike_N764K,Spike_L212I,NSP6_G107del,Spike_R346K,NSP6_I189V,Spike_T547K,M_D3G,Spike_D796Y,N_G204R,Spike_V143del,M_A63T,Spike_K417N,NSP6_S106del,Spike_S371L,Spike_G339D,NSP3_S1265del,NSP14_I42V,Spike_P681H,Spike_Y144del,Spike_ins214EPE,N_S33del,Spike_S375F,Spike_G446S,N_E31del,NSP3_K38R,Spike_N211del,Spike_E484A,E_T9I,Spike_V70del,Spike_L981F,NSP12_P323L,Spike_Y145del,Spike_D614G)
EPI_ISL_9012698	17/01/2022	BA.1	BA.1	GRA	(NSP5_P132H,Spike_H69del,Spike_T95I,Spike_A67V,Spike_S373P,Spike_N969K,Spike_H655Y,Spike_N856K,N_R203K,Spike_G142D,NSP3_A1892T,Spike_Q954H,N_P13L,NSP3_L1266I,N_R32del,M_Q19E,NSP4_T492I,NSP6_L105del,Spike_N679K,Spike_N764K,Spike_L212I,NSP6_G107del,NSP6_I189V,Spike_T547K,M_D3G,Spike_D796Y,N_G204R,Spike_V143del,M_A63T,NSP1_M85del,NS3_H78Y,Spike_K417N,NSP6_S106del,Spike_S371L,Spike_G339D,NSP3_S1265del,NSP14_I42V,Spike_P681H,Spike_Y144del,Spike_ins214EPE,N_S33del,Spike_S375F,N_E31del,NSP3_K38R,Spike_N211del,E_T9I,Spike_V70del,Spike_L981F,NSP12_P323L,Spike_Y145del,Spike_D614G,N_A398V)
EPI_ISL_9012702	17/01/2022	BA.1	BA.1.1	GRA	(NSP5_P132H,Spike_H69del,Spike_T95I,Spike_A67V,Spike_S373P,Spike_N969K,Spike_H655Y,Spike_N856K,N_R203K,Spike_G142D,NSP3_A1892T,Spike_Q954H,N_P13L,NSP3_L1266I,N_R32del,M_Q19E,Spike_N440K,NSP4_T492I,NSP6_L105del,Spike_N679K,Spike_N764K,Spike_L212I,NSP6_G107del,Spike_R346K,NSP6_I189V,Spike_T547K,M_D3G,Spike_D796Y,N_G204R,Spike_V143del,M_A63T,Spike_K417N,NSP6_S106del,Spike_S371L,Spike_G339D,NSP3_S1265del,NSP14_I42V,Spike_P681H,Spike_Y144del,Spike_ins214EPE,N_S33del,Spike_S375F,Spike_G446S,N_E31del,NSP3_K38R,Spike_N211del,E_T9I,Spike_V70del,Spike_L981F,NSP12_P323L,Spike_Y145del,Spike_D614G)
EPI_ISL_9012703	17/01/2022	BA.1	BA.1.17.2	GRA	(NSP5_P132H,Spike_H69del,Spike_T95I,Spike_A67V,Spike_S373P,Spike_N969K,Spike_H655Y,Spike_N856K,N_R203K,Spike_G142D,NSP3_A1892T,Spike_Q954H,Spike_A701V,N_P13L,NSP3_L1266I,N_R32del,M_Q19E,Spike_N440K,NSP4_T492I,NSP6_L105del,Spike_N679K,Spike_N764K,Spike_L212I,NSP6_G107del,NSP6_I189V,Spike_T547K,M_D3G,Spike_D796Y,N_G204R,Spike_V143del,M_A63T,NSP3_V1069I,Spike_K417N,NSP6_S106del,Spike_S371L,Spike_G339D,NSP3_S1265del,NSP3_L1870F,NSP14_I42V,Spike_P681H,Spike_Y144del,Spike_ins214EPE,N_S33del,Spike_S375F,NSP1_F143del,Spike_G446S,NSP1_K141del,N_E31del,NSP3_K38R,Spike_N211del,NSP1_S142del,E_T9I,Spike_V70del,Spike_L981F,NSP12_P323L,Spike_Y145del,Spike_D614G)
EPI_ISL_9012704	17/01/2022	BA.1	BA.1.1	GRA	(NSP5_P132H,Spike_H69del,Spike_T95I,Spike_A67V,Spike_S373P,Spike_N969K,Spike_H655Y,Spike_N856K,N_R203K,Spike_G142D,NSP3_A1892T,Spike_Q954H,N_P13L,NSP3_L1266I,N_R32del,M_Q19E,Spike_N440K,NSP4_T492I,NSP6_L105del,Spike_N679K,Spike_N764K,Spike_L212I,NSP6_G107del,Spike_R346K,NSP6_I189V,Spike_T547K,M_D3G,Spike_D796Y,N_G204R,Spike_V143del,M_A63T,NSP12_I106V,Spike_K417N,NSP6_S106del,Spike_S371L,Spike_G339D,NSP3_S1265del,NSP14_I42V,Spike_P681H,Spike_Y144del,Spike_ins214EPE,N_S33del,Spike_S375F,NSP15_V127F,Spike_G446S,N_E31del,NSP3_K38R,Spike_N211del,E_T9I,Spike_V70del,Spike_L981F,NSP12_P323L,Spike_Y145del,Spike_D614G)
EPI_ISL_9012705	17/01/2022	BA.1	BA.1.1.1	GRA	(NSP5_P132H,Spike_H69del,Spike_T95I,Spike_A67V,Spike_S373P,Spike_N969K,Spike_H655Y,Spike_N856K,N_R203K,Spike_G142D,NSP3_A1892T,Spike_Q954H,N_P13L,NSP3_L1266I,N_R32del,M_Q19E,Spike_N440K,NSP4_T492I,NSP6_L105del,Spike_N679K,Spike_N764K,Spike_L212I,NSP6_G107del,Spike_R346K,NSP6_I189V,Spike_T547K,M_D3G,Spike_D796Y,N_G204R,Spike_V143del,M_A63T,NSP12_Q875R,Spike_K417N,NSP6_S106del,Spike_S371L,Spike_G339D,NSP3_S1265del,NSP14_I42V,Spike_P681H,Spike_Y144del,Spike_ins214EPE,N_S33del,Spike_S375F,Spike_G446S,N_E31del,NSP3_K38R,Spike_N211del,E_T9I,Spike_V70del,Spike_L981F,NSP12_P323L,Spike_Y145del,Spike_D614G)
EPI_ISL_9012709	17/01/2022	BA.1	BA.1	GRA	(NSP5_P132H,Spike_H69del,Spike_T95I,Spike_A67V,Spike_S373P,Spike_Q493R,Spike_N969K,Spike_H655Y,Spike_N856K,Spike_G142D,NSP3_A1892T,Spike_Q954H,N_P13L,NSP3_L1266I,Spike_N501Y,M_Q19E,Spike_N440K,NSP6_L105del,Spike_N679K,Spike_N764K,Spike_L212I,NSP6_G107del,Spike_Y505H,NSP6_I189V,Spike_T547K,M_D3G,Spike_D796Y,Spike_V143del,M_A63T,Spike_G496S,Spike_K417N,NSP6_S106del,Spike_S371L,Spike_G339D,NSP3_S1265del,NSP14_I42V,Spike_P681H,Spike_Y144del,Spike_S375F,Spike_Q498R,Spike_G446S,NSP3_K38R,Spike_N211del,Spike_E484A,E_T9I,Spike_V70del,Spike_L981F,NSP12_P323L,Spike_D614G,Spike_Y145del)
EPI_ISL_11148626	22/01/2022	BA.1	BA.1	GRA	(NSP5_P132H,Spike_H69del,Spike_T95I,Spike_A67V,Spike_S373P,Spike_N969K,Spike_H655Y,Spike_N856K,N_R203K,Spike_G142D,NSP3_A1892T,Spike_Q954H,N_P13L,NSP3_L1266I,NSP1_H83del,N_R32del,M_Q19E,Spike_N440K,NSP4_T492I,NSP6_L105del,Spike_N679K,Spike_N764K,Spike_L212I,NSP6_G107del,Spike_Y505H,NSP1_V84del,NSP6_I189V,Spike_T547K,M_D3G,Spike_D796Y,N_G204R,Spike_V143del,M_A63T,NSP1_M85del,Spike_K417N,NSP6_S106del,Spike_S371L,Spike_G339D,NSP10_P84S,NSP3_S1265del,NSP14_I42V,Spike_P681H,Spike_Y144del,Spike_ins214EPE,NSP1_G82del,N_S33del,Spike_S375F,Spike_G446S,N_E31del,NSP1_V86del,NSP3_K38R,Spike_N211del,E_T9I,Spike_V70del,Spike_L981F,NSP12_P323L,Spike_Y145del,Spike_D614G)
EPI_ISL_11149367	28/01/2022	BA.2	BA.2	GRA	(NSP5_P132H,NSP3_G489S,Spike_L24del,NSP4_T327I,Spike_S373P,Spike_Q493R,Spike_N969K,Spike_H655Y,N_R203K,Spike_V213G,Spike_G142D,Spike_A27S,Spike_Q954H,N_P13L,Spike_P25del,N_R32del,NS3_T223I,Spike_T19I,M_Q19E,Spike_N440K,NSP4_T492I,Spike_N679K,Spike_N764K,NSP6_G107del,Spike_D796Y,N_G204R,Spike_T478K,N_S413R,M_A63T,Spike_S371F,Spike_K417N,NSP13_R392C,Spike_T376A,NSP6_S106del,NSP2_L444F,Spike_G339D,Spike_R408S,NSP4_L438F,NSP14_I42V,NSP4_L264F,Spike_P681H,NSP3_T24I,N_S33del,NSP1_S135R,Spike_S375F,Spike_D405N,Spike_S477N,N_E31del,NSP15_T112I,NSP6_F108del,Spike_E484A,E_T9I,Spike_P26del,NSP12_P323L,Spike_D614G)

EPI_ISL_11149368	28/01/2022	BA.1	BA.1.17.2	GRA	(NSP5_P132H,Spike_H69del,Spike_T95I,Spike_A67V,Spike_S373P,Spike_N969K,Spike_H655Y,Spike_N856K,N_R203K,Spike_G142D,NSP3_A1892T,Spike_Q954H,Spike_A701V,N_P13L,NSP3_L1266I,N_R32del,Spike_A672V,M_Q19E,Spike_N440K,NSP4_T492I,NSP6_L105del,Spike_N679K,Spike_N764K,Spike_L212I,NSP6_G107del,NSP6_I189V,Spike_T547K,M_D3G,Spike_D796Y,N_G204R,Spike_V143del,M_A63T,NSP3_V1069I,Spike_K417N,NSP6_S106del,Spike_S371L,Spike_G339D,Spike_R158del,NSP3_S1265del,NSP14_I42V,Spike_P681H,Spike_Y144del,Spike_ins214EPE,Spike_F157del,N_S33del,Spike_S375F,Spike_G446S,N_E31del,Spike_E156G,NSP3_K38R,Spike_N211del,NSP3_N1293S,E_T9I,Spike_V70del,Spike_L981F,NSP12_P323L,Spike_Y145del,Spike_D614G)
EPI_ISL_13566029	28/01/2022	BA.1	BA.1.1.1	GRA	(NSP5_P132H,Spike_H69del,Spike_T95I,Spike_A67V,Spike_S373P,Spike_Q493R,Spike_N969K,Spike_H655Y,Spike_N856K,N_R203K,Spike_G142D,NSP3_A1892T,Spike_Q954H,N_P13L,NSP3_L1266I,Spike_N501Y,N_R32del,M_Q19E,Spike_N440K,NSP4_T492I,NSP6_L105del,Spike_N679K,Spike_N764K,Spike_L212I,NSP6_G107del,Spike_Y505H,Spike_R346K,NSP6_I189V,Spike_T547K,M_D3G,Spike_D796Y,N_G204R,Spike_V143del,M_A63T,Spike_G496S,NSP12_Q875R,NSP3_L198F,Spike_K417N,NSP6_S106del,Spike_S371L,Spike_G339D,NSP3_S1265del,NSP14_I42V,Spike_P681H,Spike_Y144del,Spike_ins214EPE,N_S33del,Spike_S375F,Spike_Q498R,Spike_G446S,NS3_A54V,N_E31del,NSP3_K38R,Spike_N211del,Spike_E484A,E_T9I,Spike_V70del,Spike_L981F,NSP12_P323L,Spike_D614G,Spike_Y145del)
EPI_ISL_9593340	31/01/2022	BA.1	BA.1.1	GRA	(NSP5_P132H,Spike_H69del,Spike_T95I,Spike_A67V,Spike_S373P,Spike_N969K,Spike_H655Y,Spike_N856K,N_R203K,Spike_G142D,E_G10C,NSP3_A1892T,Spike_Q954H,N_P13L,NSP3_L1266I,N_R32del,M_Q19E,Spike_N440K,NSP4_T492I,NSP6_L105del,Spike_N679K,Spike_N764K,Spike_L212I,NSP6_G107del,NSP13_G170S,Spike_R346K,NSP6_I189V,Spike_T547K,M_D3G,Spike_D796Y,N_G204R,Spike_V143del,M_A63T,Spike_K417N,NSP6_S106del,Spike_S371L,Spike_G339D,NSP3_S1265del,NSP14_I42V,Spike_P681H,Spike_Y144del,Spike_ins214EPE,N_S33del,Spike_S375F,Spike_G446S,N_E31del,E_P54L,NSP3_K38R,Spike_N211del,E_T9I,Spike_V70del,Spike_L981F,NSP12_P323L,NSP2_G12S,Spike_Y145del,Spike_D614G)
EPI_ISL_9593343	31/01/2022	BA.1	BA.1.1	GRA	(NSP5_P132H,Spike_H69del,Spike_T95I,Spike_A67V,Spike_S373P,Spike_N969K,Spike_H655Y,Spike_N856K,N_R203K,Spike_G142D,NSP3_A1892T,Spike_Q954H,N_P13L,NSP3_L1266I,N_R32del,M_Q19E,Spike_N440K,NSP4_T492I,NSP6_L105del,NSP2_L289I,Spike_N679K,Spike_N764K,Spike_L212I,NSP6_G107del,Spike_R346K,NSP6_I189V,Spike_T547K,M_D3G,Spike_D796Y,N_G204R,Spike_V143del,M_A63T,Spike_K417N,NSP6_S106del,Spike_S371L,Spike_G339D,NSP3_S1265del,NSP14_I42V,Spike_P681H,Spike_Y144del,Spike_ins214EPE,N_S33del,Spike_S375F,Spike_G446S,N_E31del,NSP3_K38R,Spike_N211del,E_T9I,Spike_V70del,Spike_L981F,NSP12_P323L,Spike_Y145del,Spike_D614G)
EPI_ISL_9593345	31/01/2022	BA.1	BA.1.17.2	GRA	(NSP5_P132H,Spike_H69del,Spike_T95I,Spike_A67V,Spike_S373P,Spike_N969K,Spike_H655Y,Spike_N856K,N_R203K,Spike_G142D,NSP3_A1892T,Spike_Q954H,Spike_A701V,N_P13L,NSP3_L1266I,N_R32del,M_Q19E,Spike_N440K,NSP4_T492I,NSP6_L105del,Spike_N679K,Spike_N764K,Spike_L212I,NSP6_G107del,NSP6_I189V,Spike_T547K,M_D3G,Spike_D796Y,N_G204R,Spike_V143del,M_A63T,NSP3_V1069I,Spike_K417N,NSP6_S106del,Spike_S371L,Spike_G339D,NSP3_S1265del,NSP14_I42V,Spike_P681H,Spike_Y144del,Spike_ins214EPE,N_S33del,Spike_S375F,Spike_G446S,N_E31del,NSP3_K38R,Spike_N211del,E_T9I,Spike_V70del,Spike_L981F,NSP12_P323L,Spike_Y145del,Spike_D614G)
EPI_ISL_9593348	31/01/2022	BA.1	BA.1.17.2	GRA	(NSP5_P132H,Spike_H69del,Spike_T95I,Spike_A67V,Spike_S373P,Spike_N969K,Spike_H655Y,Spike_N856K,N_R203K,Spike_G142D,NSP3_A1892T,Spike_Q954H,Spike_A701V,N_P13L,NSP3_L1266I,N_R32del,M_Q19E,Spike_N440K,NSP4_T492I,NSP6_L105del,Spike_N679K,Spike_N764K,Spike_L212I,NSP6_G107del,NSP6_I189V,Spike_T547K,M_D3G,Spike_D796Y,N_G204R,Spike_V143del,M_A63T,NSP3_V1069I,Spike_K417N,NSP6_S106del,Spike_S371L,Spike_G339D,NSP3_S1265del,NSP3_D271G,NSP14_I42V,Spike_P681H,Spike_Y144del,Spike_ins214EPE,N_S33del,Spike_S375F,Spike_G446S,N_E31del,NSP3_K38R,Spike_N211del,E_T9I,Spike_V70del,Spike_L981F,NSP12_P323L,Spike_Y145del,Spike_D614G)
EPI_ISL_9593351	31/01/2022	BA.1	BA.1.17.2	GRA	(NSP5_P132H,Spike_H69del,Spike_T95I,Spike_A67V,Spike_S373P,Spike_N969K,Spike_H655Y,Spike_N856K,N_R203K,Spike_G142D,NSP3_A1892T,Spike_Q954H,Spike_A701V,N_P13L,NSP3_L1266I,N_R32del,M_Q19E,Spike_N440K,NSP4_T492I,NSP6_L105del,Spike_N679K,Spike_N764K,Spike_L212I,NSP6_G107del,NS7a_G38V,NSP6_I189V,NSP14_M62L,Spike_T547K,M_D3G,Spike_D796Y,N_G204R,Spike_V143del,M_A63T,NSP3_V1069I,Spike_K417N,NSP6_S106del,Spike_S371L,Spike_G339D,NSP3_S1265del,NSP14_I42V,Spike_P681H,Spike_Y144del,Spike_ins214EPE,NS3_E226G,N_S33del,Spike_S375F,Spike_G446S,N_E31del,NSP3_K38R,Spike_N211del,E_T9I,Spike_V70del,Spike_L981F,NSP12_P323L,Spike_Y145del,Spike_D614G)
EPI_ISL_9593796	31/01/2022	BA.1	BA.1.17.2	GRA	(NSP5_P132H,Spike_H69del,Spike_T95I,Spike_A67V,Spike_S373P,Spike_N969K,Spike_H655Y,Spike_N856K,N_R203K,Spike_G142D,NSP3_A1892T,Spike_Q954H,Spike_A701V,N_P13L,NSP3_L1266I,N_R32del,M_Q19E,Spike_N440K,NSP4_T492I,NSP6_L105del,NSP13_H164Y,Spike_N679K,Spike_N764K,Spike_L212I,NSP6_G107del,NSP6_I189V,Spike_T547K,M_D3G,Spike_D796Y,N_G204R,Spike_V143del,M_A63T,NSP3_V1069I,Spike_K417N,NSP6_S106del,Spike_S371L,Spike_G339D,NSP3_S1265del,NSP14_I42V,Spike_P681H,Spike_Y144del,Spike_ins214EPE,N_S33del,Spike_S375F,M_I76V,Spike_G446S,N_E31del,NSP3_K38R,Spike_N211del,E_T9I,Spike_V70del,Spike_L981F,NSP12_P323L,Spike_Y145del,Spike_D614G)
EPI_ISL_9593868	31/01/2022	BA.1	BA.1.17.2	GRA	(NSP5_P132H,Spike_H69del,Spike_T95I,Spike_A67V,Spike_S373P,Spike_N969K,Spike_H655Y,Spike_N856K,N_R203K,Spike_G142D,NSP3_A1892T,Spike_Q954H,Spike_A701V,N_P13L,NSP3_L1266I,N_R32del,M_Q19E,Spike_N440K,NSP4_T492I,NSP6_L105del,NSP13_H164Y,Spike_N679K,Spike_N764K,Spike_L212I,NSP6_G107del,NSP6_I189V,Spike_T547K,M_D3G,Spike_D796Y,N_G204R,Spike_V143del,M_A63T,NSP3_V1069I,Spike_K417N,NSP6_S106del,Spike_S371L,Spike_G339D,NSP3_S1265del,NSP14_I42V,Spike_P681H,Spike_Y144del,Spike_ins214EPE,N_S33del,Spike_S375F,M_I76V,Spike_G446S,N_E31del,NSP3_K38R,Spike_N211del,E_T9I,Spike_V70del,Spike_L981F,NSP12_P323L,Spike_Y145del,Spike_D614G)
EPI_ISL_9594151	31/01/2022	BA.1	BA.1.17	GRA	(NSP5_P132H,Spike_H69del,Spike_T95I,Spike_A67V,Spike_S373P,Spike_N969K,Spike_H655Y,Spike_N856K,N_R203K,Spike_G142D,NSP3_A1892T,Spike_Q954H,N_P13L,NSP3_L1266I,N_R32del,M_Q19E,Spike_N440K,NSP4_T492I,NSP6_L105del,Spike_N679K,Spike_N764K,Spike_L212I,NSP6_G107del,NS8_A51V,NSP6_I189V,Spike_T547K,M_D3G,Spike_D796Y,N_G204R,Spike_V143del,M_A63T,NSP3_P985S,NSP3_V1069I,Spike_K417N,NSP6_S106del,Spike_S371L,Spike_G339D,NSP3_S1265del,NSP14_I42V,Spike_P681H,Spike_Y144del,Spike_ins214EPE,N_S33del,Spike_S375F,Spike_G446S,NSP3_A1279T,N_E31del,NSP3_K38R,Spike_N211del,E_T9I,Spike_V70del,Spike_L981F,NSP12_P323L,Spike_Y145del,Spike_D614G)
EPI_ISL_9594205	31/01/2022	BA.1	BA.1.15.1	GRA	(NSP5_P132H,Spike_H69del,Spike_T95I,Spike_A67V,Spike_S373P,Spike_N969K,Spike_H655Y,Spike_N856K,N_R203K,Spike_G142D,NSP3_A1892T,Spike_Q954H,NS3_L106F,N_P13L,NSP3_L1266I,N_R32del,M_Q19E,Spike_N440K,NSP4_T492I,NSP6_L105del,Spike_N679K,Spike_N764K,Spike_L212I,NSP6_G107del,NSP6_I189V,Spike_T547K,M_D3G,Spike_D796Y,N_G204R,N_D343G,Spike_V143del,M_A63T,Spike_K417N,NSP6_S106del,Spike_S371L,Spike_I1081V,Spike_G339D,NSP3_S1265del,NSP14_I42V,Spike_P681H,Spike_Y144del,Spike_ins214EPE,N_S33del,Spike_S375F,Spike_G446S,N_E31del,NSP3_K38R,Spike_N211del,E_T9I,Spike_V70del,Spike_L981F,NSP12_P323L,Spike_Y145del,Spike_D614G)

EPI_ISL_9594207	31/01/2022	BA.1	BA.1.15	GRA	(NSP5_P132H,Spike_H69del,Spike_T95I,Spike_A67V,Spike_S373P,Spike_N969K,Spike_H655Y,Spike_N856K,N_R203K,Spike_G142D,NSP3_A1892T,Spike_Q954H,NS3_L106F,N_P13L,NSP3_L1266I,N_R32del,M_Q19E,Spike_N440K,NSP4_T492I,NSP6_L105del,Spike_N679K,Spike_N764K,Spike_L212I,NSP6_G107del,NSP6_I189V,Spike_T547K,M_D3G,Spike_D796Y,N_G204R,N_D343G,Spike_V143del,M_A63T,Spike_K417N,NSP6_S106del,Spike_S371L,Spike_G339D,Spike_P1162S,NSP3_S1265del,NSP14_I42V,Spike_P681H,Spike_Y144del,Spike_ins214EPE,N_S33del,Spike_S375F,Spike_G446S,N_E31del,NSP3_K38R,Spike_N211del,E_T9I,Spike_V70del,Spike_L981F,NSP12_P323L,Spike_Y145del,Spike_D614G)
EPI_ISL_9594210	31/01/2022	BA.1	BA.1.1	GRA	(NSP5_P132H,Spike_H69del,Spike_T95I,Spike_A67V,Spike_S373P,Spike_N969K,Spike_H655Y,Spike_N856K,N_R203K,Spike_G142D,NSP3_A1892T,Spike_Q954H,N_P13L,NSP3_L1266I,N_R32del,M_Q19E,Spike_N440K,NSP4_T492I,NSP6_L105del,Spike_N679K,Spike_N764K,Spike_L212I,NSP6_G107del,Spike_R346K,NSP6_I189V,Spike_T547K,M_D3G,Spike_D796Y,N_G204R,Spike_V143del,M_A63T,Spike_K417N,NSP6_S106del,Spike_S371L,Spike_G339D,NSP3_S1265del,NSP14_I42V,Spike_P681H,Spike_Y144del,Spike_ins214EPE,N_S33del,Spike_S375F,Spike_G446S,N_E31del,NSP3_K38R,Spike_N211del,E_T9I,Spike_V70del,Spike_L981F,NSP12_P323L,Spike_Y145del,Spike_D614G)
EPI_ISL_9594212	31/01/2022	BA.1	BA.1.1	GRA	(NSP5_P132H,Spike_H69del,Spike_T95I,Spike_A67V,Spike_S373P,Spike_N969K,Spike_H655Y,Spike_N856K,N_R203K,Spike_G142D,NSP3_A1892T,Spike_Q954H,N_P13L,NSP3_L1266I,N_R32del,M_Q19E,Spike_N440K,NSP4_T492I,NSP6_L105del,Spike_N679K,Spike_N764K,Spike_L212I,NSP6_G107del,Spike_R346K,NSP6_I189V,Spike_T547K,M_D3G,Spike_D796Y,N_G204R,Spike_V143del,M_A63T,Spike_K417N,NSP6_S106del,Spike_S371L,Spike_G339D,NSP3_S1265del,NSP14_I42V,Spike_P681H,Spike_Y144del,Spike_ins214EPE,N_S33del,Spike_S375F,Spike_G446S,N_E31del,NSP3_K38R,Spike_N211del,E_T9I,Spike_V70del,Spike_L981F,NSP12_P323L,Spike_Y145del,Spike_D614G)
EPI_ISL_9594215	31/01/2022	BA.1	BA.1.1	GRA	(NSP5_P132H,Spike_H69del,Spike_T95I,Spike_A67V,Spike_S373P,Spike_N969K,Spike_H655Y,Spike_N856K,N_R203K,Spike_G142D,NSP3_A1892T,Spike_Q954H,N_P13L,NSP3_L1266I,N_R32del,M_Q19E,Spike_N440K,NSP4_T492I,NSP6_L105del,Spike_N679K,Spike_N764K,Spike_L212I,NSP6_G107del,Spike_R346K,NSP6_I189V,Spike_T547K,M_D3G,Spike_D796Y,N_G204R,Spike_V143del,M_A63T,Spike_K417N,NSP6_S106del,Spike_S371L,Spike_G339D,NSP3_S1265del,NSP14_I42V,Spike_P681H,Spike_Y144del,Spike_ins214EPE,N_S33del,Spike_S375F,Spike_G446S,N_E31del,NSP3_K38R,Spike_N211del,E_T9I,Spike_V70del,Spike_L981F,NSP12_P323L,Spike_Y145del,Spike_D614G)
EPI_ISL_9594300	31/01/2022	BA.1	BA.1.1	GRA	(NSP5_P132H,Spike_H69del,NSP2_T170I,Spike_T95I,NSP3_T1465I,Spike_A67V,Spike_S373P,Spike_N969K,Spike_H655Y,Spike_N856K,N_R203K,Spike_G142D,NSP3_A1892T,Spike_Q954H,N_P13L,NSP3_L1266I,N_R32del,M_Q19E,Spike_N440K,NSP4_T492I,NSP6_L105del,Spike_N679K,Spike_N764K,Spike_L212I,NSP6_G107del,Spike_R346K,NSP6_I189V,NSP3_E134G,Spike_T547K,M_D3G,NSP10_D131G,Spike_D796Y,N_G204R,Spike_V143del,M_A63T,NSP6_M183I,NSP13_E365D,Spike_K417N,NSP6_S106del,Spike_S371L,Spike_G339D,NSP3_S1265del,NSP14_I42V,Spike_P681H,Spike_Y144del,Spike_ins214EPE,N_S33del,Spike_S375F,Spike_G446S,N_E31del,NSP3_K38R,Spike_N211del,E_T9I,Spike_V70del,Spike_L981F,NSP12_P323L,Spike_Y145del,Spike_D614G)
EPI_ISL_9594313	31/01/2022	BA.1	BA.1.17.2	GRA	(NSP5_P132H,Spike_H69del,Spike_T95I,Spike_A67V,Spike_S373P,Spike_N969K,Spike_H655Y,Spike_N856K,N_R203K,Spike_G142D,NSP3_A1892T,Spike_Q954H,Spike_A701V,N_P13L,NSP3_L1266I,N_R32del,M_Q19E,Spike_N440K,NSP4_T492I,NSP6_L105del,NSP13_H164Y,Spike_N679K,Spike_N764K,Spike_L212I,NSP6_G107del,NSP6_I189V,Spike_T547K,M_D3G,Spike_D796Y,N_G204R,Spike_V143del,M_A63T,NSP3_V1069I,Spike_K417N,NSP6_S106del,Spike_S371L,Spike_G339D,NSP3_S1265del,NSP14_I42V,Spike_P681H,Spike_Y144del,Spike_ins214EPE,N_S33del,Spike_S375F,M_I76V,Spike_G446S,N_E31del,NSP3_K38R,Spike_N211del,E_T9I,Spike_V70del,Spike_L981F,NSP12_P323L,Spike_Y145del,Spike_D614G)
EPI_ISL_9594314	31/01/2022	BA.1	BA.1.17.2	GRA	(NSP5_P132H,Spike_H69del,Spike_T95I,Spike_A67V,Spike_S373P,Spike_N969K,Spike_H655Y,Spike_N856K,N_R203K,Spike_G142D,NSP3_A1892T,Spike_Q954H,Spike_A701V,N_P13L,NSP3_L1266I,N_R32del,M_Q19E,Spike_N440K,NSP4_T492I,NSP6_L105del,NSP13_H164Y,Spike_N679K,Spike_N764K,Spike_L212I,NSP6_G107del,NSP6_I189V,Spike_T547K,M_D3G,Spike_D796Y,N_G204R,Spike_V143del,M_A63T,NSP3_V1069I,Spike_K417N,NSP6_S106del,Spike_S371L,Spike_G339D,NSP3_S1265del,NSP14_I42V,Spike_P681H,Spike_Y144del,Spike_ins214EPE,N_S33del,Spike_S375F,M_I76V,Spike_G446S,N_E31del,NSP3_K38R,Spike_N211del,E_T9I,Spike_V70del,Spike_L981F,NSP12_P323L,Spike_Y145del,Spike_D614G)
EPI_ISL_9594315	31/01/2022	BA.1	BA.1	GRA	(NSP5_P132H,Spike_H69del,Spike_T95I,Spike_A67V,Spike_S373P,Spike_N969K,Spike_H655Y,Spike_N856K,N_R203K,Spike_G142D,NSP3_A1892T,Spike_Q954H,N_P13L,NSP3_L1266I,N_R32del,M_Q19E,Spike_N440K,NSP4_T492I,NSP6_L105del,Spike_N679K,Spike_N764K,Spike_L212I,NSP6_G107del,NSP6_I189V,Spike_T547K,M_D3G,Spike_D796Y,N_G204R,Spike_V143del,M_A63T,Spike_K417N,NSP6_S106del,Spike_S371L,Spike_G339D,NSP3_S1265del,NSP14_I42V,Spike_P681H,Spike_Y144del,Spike_ins214EPE,N_S33del,Spike_S375F,Spike_G446S,N_E31del,NSP3_K38R,Spike_N211del,E_T9I,Spike_V70del,Spike_L981F,NSP12_P323L,Spike_Y145del,Spike_D614G)
EPI_ISL_9594316	31/01/2022	BA.1	BA.1.15.1	GRA	(NSP5_P132H,Spike_H69del,Spike_T95I,Spike_A67V,Spike_S373P,Spike_N969K,Spike_H655Y,Spike_N856K,N_R203K,Spike_G142D,NSP3_A1892T,Spike_Q954H,NS3_L106F,N_P13L,NSP3_L1266I,N_R32del,M_Q19E,Spike_N440K,NSP4_T492I,NSP6_L105del,Spike_N679K,Spike_N764K,Spike_L212I,NSP6_G107del,NSP6_I189V,Spike_T547K,M_D3G,Spike_D796Y,N_G204R,N_D343G,Spike_V143del,M_A63T,Spike_K417N,NSP6_S106del,Spike_S371L,Spike_I1081V,Spike_G339D,NSP3_S1265del,NSP14_I42V,Spike_P681H,Spike_Y144del,Spike_ins214EPE,N_S33del,Spike_S375F,Spike_G446S,N_E31del,NSP3_K38R,Spike_N211del,E_T9I,Spike_V70del,Spike_L981F,NSP12_P323L,Spike_Y145del,Spike_D614G)
EPI_ISL_9594317	31/01/2022	BA.1	BA.1.15	GRA	(NSP5_P132H,Spike_H69del,Spike_T95I,Spike_A67V,Spike_S373P,Spike_N969K,Spike_H655Y,Spike_N856K,N_R203K,Spike_G142D,NSP3_A1892T,Spike_Q954H,NS3_L106F,N_P13L,NSP3_L1266I,N_R32del,M_Q19E,NSP4_T492I,NSP6_L105del,Spike_N679K,Spike_N764K,Spike_L212I,NSP6_G107del,NSP6_I189V,Spike_T547K,M_D3G,Spike_D796Y,N_G204R,N_D343G,Spike_V143del,M_A63T,Spike_K417N,NSP6_S106del,Spike_S371L,Spike_G339D,Spike_P1162S,NSP3_S1265del,NSP14_I42V,Spike_P681H,Spike_Y144del,Spike_ins214EPE,N_S33del,Spike_S375F,N_E31del,NSP3_K38R,Spike_N211del,NSP13_P53S,E_T9I,Spike_V70del,Spike_L981F,NSP12_P323L,Spike_Y145del,Spike_D614G)
EPI_ISL_9594318	31/01/2022	BA.1	BA.1.1	GRA	(NSP5_P132H,Spike_H69del,Spike_T95I,Spike_A67V,Spike_S373P,Spike_N969K,Spike_H655Y,Spike_N856K,N_R203K,Spike_G142D,NSP3_A1892T,Spike_Q954H,N_P13L,NSP3_L1266I,N_R32del,M_Q19E,Spike_N440K,NSP4_T492I,NSP6_L105del,Spike_N679K,NSP3_T73I,Spike_N764K,Spike_L212I,NSP6_G107del,Spike_R346K,NSP6_I189V,Spike_T547K,M_D3G,Spike_D796Y,N_G204R,Spike_V143del,M_A63T,Spike_K417N,NSP6_S106del,Spike_S371L,Spike_G339D,NSP3_S1265del,NSP14_I42V,Spike_P681H,Spike_Y144del,Spike_ins214EPE,N_S33del,Spike_S375F,Spike_G446S,N_E31del,NSP3_K38R,Spike_N211del,E_T9I,Spike_V70del,Spike_L981F,NSP12_P323L,Spike_Y145del,Spike_D614G)

EPI_ISL_9594319	31/01/2022	BA.1	BA.1.1	GRA	(NSP5_P132H,Spike_H69del,Spike_T95I,Spike_A67V,Spike_S373P,Spike_N969K,Spike_H655Y,Spike_N856K,N_R203K,Spike_G142D,NSP3_A1892T,Spike_Q954H,N_P13L,NSP3_L1266I,N_R32del,M_Q19E,Spike_N440K,NSP4_T492I,NSP6_L105del,Spike_N679K,Spike_N764K,Spike_L212I,NSP6_G107del,Spike_R346K,NSP6_I189V,Spike_T547K,M_D3G,Spike_D796Y,N_G204R,Spike_V143del,M_A63T,Spike_K417N,NSP6_S106del,Spike_S371L,Spike_G339D,NSP3_S1265del,NSP14_I42V,Spike_P681H,Spike_Y144del,Spike_ins214EPE,N_S33del,Spike_S375F,Spike_G446S,N_E31del,NSP3_K38R,Spike_N211del,E_T9I,Spike_V70del,Spike_L981F,NSP12_P323L,Spike_Y145del,Spike_D614G)
EPI_ISL_9594320	31/01/2022	BA.1	BA.1.17	GRA	(NSP5_P132H,NSP1_V106I,Spike_H69del,Spike_T95I,Spike_A67V,Spike_S373P,Spike_N969K,Spike_H655Y,Spike_N856K,N_R203K,Spike_G142D,NSP3_A1892T,Spike_Q954H,N_P13L,NSP3_L1266I,N_R32del,M_Q19E,Spike_N440K,NSP4_T492I,NSP6_L105del,Spike_N679K,Spike_N764K,Spike_L212I,NSP6_G107del,NSP6_I189V,Spike_T547K,M_D3G,Spike_D796Y,N_G204R,Spike_V143del,M_A63T,NSP3_P985S,NSP3_V1069I,Spike_K417N,NSP6_S106del,Spike_S371L,Spike_G339D,NSP3_S1265del,NSP7_L71F,NSP14_I42V,Spike_P681H,Spike_Y144del,Spike_ins214EPE,N_S33del,Spike_S375F,Spike_G446S,N_E31del,NSP3_K38R,Spike_N211del,E_T9I,Spike_V70del,Spike_L981F,NSP12_P323L,Spike_Y145del,Spike_D614G)
EPI_ISL_9594321	31/01/2022	BA.1	BA.1	GRA	(NSP5_P132H,Spike_H69del,Spike_T95I,Spike_A67V,Spike_S373P,Spike_N969K,Spike_H655Y,Spike_N856K,N_R203K,Spike_G142D,NSP3_A1892T,Spike_Q954H,N_P13L,NSP3_L1266I,N_R32del,NSP4_P123S,M_Q19E,Spike_N440K,NSP4_T492I,NSP6_L105del,Spike_N679K,Spike_N764K,Spike_L212I,NSP6_G107del,NSP6_I189V,Spike_T547K,M_D3G,Spike_D796Y,N_G204R,Spike_V143del,M_A63T,NSP4_S127G,Spike_K417N,NSP6_S106del,Spike_S371L,Spike_G339D,NSP3_S1265del,NSP14_I42V,Spike_P681H,Spike_Y144del,Spike_ins214EPE,N_S33del,Spike_S375F,Spike_G446S,N_E31del,NSP3_K38R,Spike_N211del,NSP3_T787I,E_T9I,Spike_V70del,Spike_L981F,NSP12_P323L,Spike_Y145del,Spike_D614G)
EPI_ISL_9594322	31/01/2022	BA.1	BA.1.21.1	GRA	(NSP5_P132H,Spike_H69del,Spike_T95I,Spike_A67V,NS7b_E3stop,Spike_S373P,Spike_N969K,Spike_H655Y,Spike_N856K,N_R203K,Spike_G142D,NSP3_A1892T,Spike_Q954H,N_P13L,NSP3_L1266I,N_R32del,M_Q19E,Spike_N440K,NSP4_T492I,NSP6_L105del,Spike_N679K,Spike_N764K,Spike_L212I,NSP6_G107del,NSP6_I189V,Spike_T547K,M_D3G,Spike_D796Y,N_G204R,Spike_V143del,M_A63T,Spike_K417N,NSP6_S106del,Spike_S371L,Spike_G339D,NSP3_S1265del,NSP14_I42V,Spike_P681H,NSP6_V114I,Spike_Y144del,Spike_ins214EPE,N_S33del,Spike_S375F,Spike_G446S,N_E31del,NSP3_K38R,Spike_N211del,E_T9I,Spike_V70del,Spike_L981F,NSP12_P323L,Spike_Y145del,Spike_D614G,NSP12_L749M)
EPI_ISL_9594323	31/01/2022	BA.1	BA.1.15.1	GRA	(NSP5_P132H,Spike_H69del,Spike_T95I,Spike_A67V,Spike_S373P,Spike_N969K,Spike_H655Y,Spike_N856K,N_R203K,Spike_G142D,NSP3_A1892T,Spike_Q954H,NS3_L106F,N_P13L,NSP3_L1266I,N_R32del,M_Q19E,Spike_N440K,NSP4_T492I,NSP6_L105del,Spike_N679K,Spike_N764K,Spike_L212I,NSP6_G107del,NSP6_I189V,Spike_T547K,M_D3G,Spike_D796Y,N_G204R,N_D343G,Spike_V143del,M_A63T,Spike_K417N,NSP6_S106del,Spike_S371L,Spike_I1081V,Spike_G339D,NSP3_S1265del,NSP14_I42V,Spike_P681H,Spike_Y144del,Spike_ins214EPE,N_S33del,Spike_S375F,Spike_G446S,N_E31del,NSP3_K38R,Spike_N211del,E_T9I,Spike_V70del,Spike_L981F,NSP12_P323L,Spike_Y145del,Spike_D614G)
EPI_ISL_9594324	31/01/2022	BA.2	BA.2	GRA	(NSP5_P132H,NSP3_G489S,Spike_L24del,NSP4_T327I,Spike_S373P,Spike_N969K,Spike_H655Y,N_R203K,Spike_V213G,Spike_G142D,Spike_A27S,Spike_Q954H,N_P13L,Spike_P25del,N_R32del,NS3_T223I,Spike_T19I,M_Q19E,Spike_N440K,NSP4_T492I,Spike_N679K,Spike_N764K,NSP6_G107del,Spike_D796Y,N_G204R,Spike_T478K,N_S413R,M_A63T,Spike_S371F,Spike_K417N,NSP13_R392C,Spike_T376A,NSP6_S106del,Spike_G339D,Spike_R408S,NSP4_L438F,NSP14_I42V,Spike_P681H,NSP4_L264F,NSP3_T24I,N_S33del,NSP1_S135R,Spike_S375F,Spike_D405N,Spike_S477N,N_E31del,NSP15_T112I,NSP6_F108del,Spike_E484A,E_T9I,Spike_P26del,NSP12_P323L,Spike_D614G)
EPI_ISL_9594325	31/01/2022	BA.1	BA.1	GRA	(NSP5_P132H,Spike_H69del,Spike_T95I,Spike_A67V,Spike_S373P,Spike_N969K,Spike_H655Y,Spike_N856K,N_R203K,Spike_G142D,NSP3_A1892T,Spike_Q954H,N_P13L,NSP3_L1266I,N_R32del,M_Q19E,Spike_N440K,NSP4_T492I,NSP6_L105del,Spike_N679K,Spike_N764K,Spike_L212I,NSP6_G107del,NSP6_I189V,Spike_T547K,M_D3G,Spike_D796Y,N_G204R,Spike_V143del,M_A63T,Spike_K417N,NSP6_S106del,Spike_S371L,Spike_G339D,NSP3_S1265del,NSP14_I42V,Spike_P681H,Spike_Y144del,Spike_ins214EPE,N_S33del,Spike_S375F,Spike_G446S,N_E31del,NSP3_K38R,Spike_N211del,E_T9I,Spike_V70del,Spike_L981F,NSP12_P323L,Spike_Y145del,Spike_D614G,NSP4_N115K)
EPI_ISL_9594326	31/01/2022	BA.1	BA.1.1	GRA	(NSP5_P132H,Spike_H69del,Spike_T95I,Spike_A67V,Spike_S373P,Spike_N969K,Spike_H655Y,Spike_N856K,N_R203K,Spike_G142D,NSP3_A1892T,Spike_Q954H,N_P13L,NSP3_L1266I,N_R32del,M_Q19E,Spike_N440K,NSP4_T492I,NSP6_L105del,Spike_N679K,Spike_N764K,Spike_L212I,NSP6_G107del,NSP13_G170S,Spike_R346K,NSP6_I189V,Spike_T547K,M_D3G,Spike_D796Y,N_G204R,Spike_V143del,M_A63T,Spike_K417N,NSP6_S106del,Spike_S371L,NSP14_L177F,Spike_G339D,NSP3_S1265del,NSP14_I42V,Spike_P681H,Spike_Y144del,Spike_ins214EPE,N_S33del,Spike_S375F,Spike_G446S,N_E31del,NSP3_K38R,Spike_N211del,E_T9I,Spike_V70del,Spike_L981F,NSP12_P323L,Spike_Y145del,Spike_D614G)
EPI_ISL_9596529	31/01/2022	BA.1	BA.1.1.14	GRA	(NSP5_P132H,Spike_H69del,Spike_T95I,Spike_A67V,Spike_S373P,Spike_N969K,Spike_H655Y,Spike_N856K,N_R203K,Spike_G142D,NSP3_A1892T,Spike_Q954H,N_P13L,NSP3_L1266I,N_R32del,M_Q19E,Spike_N440K,NSP4_T492I,NSP6_L105del,Spike_N679K,Spike_N764K,Spike_L212I,NSP6_G107del,Spike_R346K,NSP6_I189V,Spike_T547K,M_D3G,Spike_D796Y,N_G204R,NSP12_I223V,Spike_V143del,M_A63T,Spike_K417N,NSP6_S106del,Spike_S371L,Spike_G339D,NSP3_S1265del,NSP14_I42V,Spike_P681H,Spike_Y144del,Spike_ins214EPE,N_S33del,Spike_S375F,Spike_G446S,N_E31del,NSP3_K38R,Spike_N211del,E_T9I,Spike_V70del,Spike_L981F,NSP12_P323L,Spike_Y145del,Spike_D614G)
EPI_ISL_9596531	31/01/2022	BA.1	BA.1.1	GRA	(NSP5_P132H,Spike_H69del,Spike_T95I,Spike_A67V,Spike_S373P,Spike_N969K,Spike_H655Y,Spike_N856K,N_R203K,Spike_G142D,NSP3_A1892T,Spike_Q954H,N_P13L,NSP3_L1266I,N_R32del,M_Q19E,Spike_N440K,NSP4_T492I,NSP6_L105del,Spike_N679K,Spike_N764K,Spike_L212I,NSP6_G107del,NSP13_G170S,Spike_R346K,NSP6_I189V,Spike_T547K,M_D3G,Spike_D796Y,N_G204R,Spike_V143del,M_A63T,Spike_K417N,NSP6_S106del,Spike_S371L,NSP14_L177F,Spike_G339D,NSP3_S1265del,NSP14_I42V,Spike_P681H,Spike_Y144del,Spike_ins214EPE,N_S33del,Spike_S375F,Spike_G446S,N_E31del,NSP3_K38R,Spike_N211del,E_T9I,Spike_V70del,Spike_L981F,NSP12_P323L,Spike_Y145del,Spike_D614G)
EPI_ISL_9596533	31/01/2022	BA.1	BA.1.1	GRA	(NSP5_P132H,Spike_H69del,Spike_T95I,Spike_A67V,Spike_S373P,Spike_N969K,Spike_H655Y,Spike_N856K,N_R203K,Spike_G142D,NSP3_A1892T,Spike_Q954H,N_P13L,NSP3_L1266I,N_R32del,M_Q19E,Spike_N440K,NSP4_T492I,NSP6_L105del,Spike_N679K,Spike_N764K,Spike_L212I,NSP6_G107del,Spike_R346K,NSP3_T656I,NSP6_I189V,Spike_T547K,M_D3G,Spike_D796Y,N_G204R,Spike_V143del,M_A63T,Spike_K417N,NSP6_S106del,Spike_S371L,Spike_G339D,NSP3_S1265del,NSP14_I42V,Spike_P681H,Spike_Y144del,Spike_ins214EPE,N_S33del,Spike_S375F,Spike_G446S,N_E31del,NSP3_K38R,Spike_N211del,NSP14_S218F,E_T9I,Spike_V70del,Spike_L981F,NSP12_P323L,Spike_Y145del,Spike_D614G)

EPI_ISL_9596534	31/01/2022	BA.1	BA.1.17.2	GRA	(NSP5_P132H,Spike_H69del,Spike_T95I,Spike_A67V,Spike_S373P,Spike_N969K,Spike_H655Y,Spike_N856K,N_R203K,NSP4_L15F,Spike_G142D,NSP3_A1892T,Spike_Q954H,Spike_A701V,N_P13L,NSP3_L1266I,N_R32del,M_Q19E,Spike_N440K,NSP4_T492I,NSP6_L105del,Spike_N679K,Spike_N764K,Spike_L212I,NSP6_G107del,NSP6_I189V,Spike_T547K,M_D3G,Spike_D796Y,N_G204R,NSP12_V257F,Spike_V143del,M_A63T,NSP1_Q63L,NSP3_V1069I,NSP14_I15T,Spike_K417N,NSP6_S106del,Spike_S371L,Spike_G339D,NSP3_S1265del,NSP14_I42V,Spike_P681H,Spike_Y144del,Spike_ins214EPE,N_S33del,Spike_S375F,Spike_G446S,N_E31del,NSP3_K38R,Spike_N211del,E_T9I,Spike_V70del,Spike_L981F,NSP12_P323L,Spike_Y145del,Spike_D614G)
EPI_ISL_9596536	31/01/2022	BA.1	BA.1.17.2	GRA	(NSP5_P132H,Spike_H69del,Spike_T95I,Spike_A67V,Spike_S373P,Spike_N969K,Spike_H655Y,Spike_N856K,N_R203K,Spike_G142D,NSP3_A1892T,Spike_Q954H,Spike_A701V,N_P13L,NSP3_L1266I,N_R32del,M_Q19E,Spike_N440K,NSP4_T492I,NSP6_L105del,NSP13_H164Y,Spike_N679K,Spike_N764K,Spike_L212I,NSP6_G107del,NSP6_I189V,Spike_T547K,M_D3G,Spike_D796Y,N_G204R,Spike_V143del,M_A63T,NSP3_V1069I,Spike_K417N,NSP6_S106del,Spike_S371L,Spike_G339D,NSP3_S1265del,NSP14_I42V,Spike_P681H,Spike_Y144del,Spike_ins214EPE,N_S33del,Spike_S375F,M_I76V,Spike_G446S,N_E31del,NSP3_K38R,Spike_N211del,E_T9I,Spike_V70del,Spike_L981F,NSP12_P323L,Spike_Y145del,Spike_D614G)
EPI_ISL_9596537	31/01/2022	BA.1	BA.1.17.2	GRA	(NSP5_P132H,Spike_H69del,Spike_T95I,Spike_A67V,Spike_S373P,Spike_N969K,Spike_H655Y,Spike_N856K,N_R203K,Spike_G142D,NSP3_A1892T,Spike_Q954H,Spike_A701V,N_P13L,NSP3_L1266I,N_R32del,M_Q19E,Spike_N440K,NSP4_T492I,NSP6_L105del,NSP13_H164Y,Spike_N679K,Spike_N764K,Spike_L212I,NSP6_G107del,NSP6_I189V,Spike_T547K,M_D3G,Spike_D796Y,N_G204R,Spike_V143del,M_A63T,NSP3_V1069I,Spike_K417N,NSP6_S106del,Spike_S371L,Spike_G339D,NSP3_S1265del,NSP14_I42V,Spike_P681H,Spike_Y144del,Spike_ins214EPE,N_S33del,Spike_S375F,M_I76V,Spike_G446S,N_E31del,NSP3_K38R,Spike_N211del,E_T9I,Spike_V70del,Spike_L981F,NSP12_P323L,Spike_Y145del,Spike_D614G)
EPI_ISL_9596538	31/01/2022	BA.1	BA.1.17.2	GRA	(NSP5_P132H,Spike_H69del,Spike_T95I,Spike_A67V,Spike_S373P,Spike_N969K,Spike_H655Y,Spike_N856K,N_R203K,Spike_G142D,NSP3_A1892T,Spike_Q954H,Spike_A701V,N_P13L,NSP3_L1266I,N_R32del,M_Q19E,Spike_N440K,NSP4_T492I,NSP6_L105del,NSP13_H164Y,Spike_N679K,Spike_N764K,Spike_L212I,NSP6_G107del,NSP6_I189V,Spike_T547K,M_D3G,Spike_D796Y,N_G204R,Spike_V143del,M_A63T,NSP3_V1069I,Spike_K417N,NSP6_S106del,Spike_S371L,Spike_G339D,NSP3_S1265del,NSP14_I42V,Spike_P681H,Spike_Y144del,Spike_ins214EPE,N_S33del,Spike_S375F,M_I76V,Spike_G446S,N_E31del,NSP3_K38R,Spike_N211del,E_T9I,Spike_V70del,Spike_L981F,NSP12_P323L,Spike_Y145del,Spike_D614G)
EPI_ISL_9596539	31/01/2022	BA.1	BA.1.17.2	GRA	(NSP5_P132H,Spike_H69del,Spike_T95I,Spike_A67V,Spike_S373P,Spike_N969K,Spike_H655Y,Spike_N856K,N_R203K,Spike_G142D,NSP3_A1892T,Spike_Q954H,Spike_A701V,N_P13L,NSP3_L1266I,N_R32del,M_Q19E,Spike_N440K,NSP4_T492I,NSP6_L105del,NSP13_H164Y,Spike_N679K,Spike_N764K,Spike_L212I,NSP6_G107del,NSP6_I189V,Spike_T547K,M_D3G,Spike_D796Y,N_G204R,Spike_T478K,Spike_V143del,M_A63T,NSP3_V1069I,Spike_K417N,NSP6_S106del,Spike_S371L,Spike_G339D,NSP3_S1265del,NSP14_I42V,Spike_P681H,Spike_Y144del,Spike_ins214EPE,N_S33del,Spike_S375F,M_I76V,Spike_G446S,N_E31del,NSP3_K38R,Spike_N211del,E_T9I,Spike_V70del,Spike_L981F,NSP12_P323L,Spike_Y145del,Spike_D614G)
EPI_ISL_9596542	31/01/2022	BA.1	BA.1.17.2	GRA	(NSP5_P132H,Spike_H69del,Spike_T95I,Spike_A67V,Spike_S373P,Spike_N969K,Spike_H655Y,Spike_N856K,N_R203K,Spike_G142D,NSP3_A1892T,Spike_Q954H,Spike_A701V,N_P13L,NSP3_L1266I,N_R32del,M_Q19E,Spike_N440K,NSP4_T492I,NSP6_L105del,NSP13_H164Y,Spike_N679K,Spike_N764K,Spike_L212I,NSP6_G107del,NSP6_I189V,Spike_T547K,M_D3G,Spike_D796Y,N_G204R,Spike_V143del,M_A63T,NSP3_V1069I,Spike_K417N,NSP6_S106del,Spike_S371L,Spike_G339D,NSP3_S1265del,NSP14_I42V,Spike_P681H,Spike_Y144del,Spike_ins214EPE,N_S33del,Spike_S375F,M_I76V,Spike_G446S,N_E31del,NSP3_K38R,Spike_N211del,E_T9I,Spike_V70del,Spike_L981F,NSP12_P323L,Spike_Y145del,Spike_D614G)
EPI_ISL_9596543	31/01/2022	BA.1	BA.1.17.2	GRA	(NSP5_P132H,Spike_H69del,Spike_T95I,Spike_A67V,Spike_S373P,Spike_N969K,Spike_H655Y,Spike_N856K,N_R203K,Spike_G142D,NSP3_A1892T,Spike_Q954H,Spike_A701V,N_P13L,NSP3_L1266I,N_R32del,M_Q19E,Spike_N440K,NSP4_T492I,NSP6_L105del,NSP13_H164Y,Spike_N679K,Spike_N764K,Spike_L212I,NSP6_G107del,NSP6_I189V,Spike_T547K,M_D3G,Spike_D796Y,N_G204R,Spike_V143del,M_A63T,NSP3_V1069I,Spike_K417N,NSP6_S106del,Spike_S371L,Spike_G339D,NSP3_S1265del,NSP14_I42V,Spike_P681H,Spike_Y144del,Spike_ins214EPE,N_S33del,Spike_S375F,M_I76V,Spike_G446S,N_E31del,NSP3_K38R,Spike_N211del,E_T9I,Spike_V70del,Spike_L981F,NSP12_P323L,Spike_Y145del,Spike_D614G)
EPI_ISL_9596544	31/01/2022	BA.1	BA.1.17.2	GRA	(NSP5_P132H,Spike_H69del,Spike_T95I,Spike_A67V,Spike_S373P,Spike_N969K,Spike_H655Y,Spike_N856K,N_R203K,Spike_G142D,NSP3_A1892T,Spike_Q954H,Spike_A701V,N_P13L,NSP3_L1266I,N_R32del,M_Q19E,Spike_N440K,NSP4_T492I,NSP6_L105del,NSP13_H164Y,Spike_N679K,Spike_N764K,Spike_L212I,NSP6_G107del,NSP6_I189V,Spike_T547K,M_D3G,Spike_D796Y,N_G204R,Spike_V143del,M_A63T,NSP3_V1069I,Spike_K417N,NSP6_S106del,Spike_S371L,Spike_G339D,NSP3_S1265del,NSP14_I42V,Spike_P681H,Spike_Y144del,Spike_ins214EPE,N_S33del,Spike_S375F,M_I76V,Spike_G446S,N_E31del,NSP3_K38R,Spike_N211del,E_T9I,Spike_V70del,Spike_L981F,NSP12_P323L,Spike_Y145del,Spike_D614G)
EPI_ISL_9596546	31/01/2022	BA.1	BA.1.17.2	GRA	(NSP5_P132H,Spike_H69del,Spike_T95I,Spike_A67V,Spike_S373P,Spike_N969K,Spike_H655Y,Spike_N856K,N_R203K,Spike_G142D,NSP3_A1892T,Spike_Q954H,Spike_A701V,N_P13L,NSP3_L1266I,N_R32del,M_Q19E,Spike_N440K,NSP4_T492I,NSP6_L105del,NSP13_H164Y,Spike_N679K,Spike_N764K,Spike_L212I,NSP6_G107del,NSP6_I189V,Spike_T547K,M_D3G,Spike_D796Y,N_G204R,Spike_V143del,M_A63T,NSP3_V1069I,Spike_K417N,NSP6_S106del,Spike_S371L,Spike_G339D,NSP3_S1265del,NSP14_I42V,Spike_P681H,Spike_Y144del,Spike_ins214EPE,N_S33del,Spike_S375F,M_I76V,Spike_G446S,N_E31del,NSP3_K38R,Spike_N211del,E_T9I,Spike_V70del,Spike_L981F,NSP12_P323L,Spike_Y145del,Spike_D614G)
EPI_ISL_9596592	31/01/2022	BA.1	BA.1.17.2	GRA	(NSP5_P132H,Spike_H69del,Spike_T95I,Spike_A67V,Spike_S373P,Spike_N969K,Spike_H655Y,Spike_N856K,N_R203K,Spike_G142D,NSP3_A1892T,Spike_Q954H,Spike_A701V,N_P13L,NSP3_L1266I,N_R32del,M_Q19E,Spike_N440K,NSP4_T492I,NSP6_L105del,NSP13_H164Y,Spike_N679K,Spike_N764K,Spike_L212I,NSP6_G107del,NSP6_I189V,Spike_T547K,M_D3G,Spike_D796Y,N_G204R,Spike_V143del,M_A63T,NSP3_V1069I,Spike_K417N,NSP6_S106del,Spike_S371L,Spike_G339D,NSP3_S1265del,NSP14_I42V,Spike_P681H,Spike_Y144del,Spike_ins214EPE,N_S33del,Spike_S375F,M_I76V,Spike_G446S,N_E31del,NSP3_K38R,Spike_N211del,E_T9I,Spike_V70del,Spike_L981F,NSP12_P323L,Spike_Y145del,Spike_D614G)
EPI_ISL_9596593	31/01/2022	BA.2	BA.2.10	GRA	(NSP5_P132H,NSP3_G489S,Spike_L24del,NSP4_T327I,Spike_S373P,Spike_Q493R,Spike_N969K,Spike_H655Y,N_R203K,Spike_V213G,Spike_G142D,Spike_A27S,Spike_Q954H,N_P13L,Spike_P25del,N_R32del,NS3_T223I,Spike_T19I,M_Q19E,Spike_N440K,NSP4_T492I,Spike_N679K,Spike_N764K,NSP6_G107del,Spike_D796Y,N_G204R,Spike_T478K,N_S413R,M_A63T,NSP6_V114A,Spike_S371F,Spike_K417N,NSP13_R392C,Spike_T376A,NSP6_S106del,Spike_G339D,Spike_R408S,NSP4_L438F,NSP14_I42V,NSP4_L264F,Spike_P681H,NSP3_T24I,N_S33del,NSP1_S135R,Spike_S375F,Spike_D405N,NSP13_S36P,Spike_S477N,N_E31del,NSP15_T112I,NSP6_F108del,Spike_E484A,E_T9I,Spike_P26del,NSP12_P323L,Spike_D614G)

EPI_ISL_9596611	31/01/2022	BA.1	BA.1.1	GRA	(NSP5_P132H,Spike_H69del,Spike_T95I,Spike_A67V,Spike_S373P,Spike_N969K,Spike_H655Y,Spike_N856K,N_R203K,Spike_G142D,NSP3_A1892T,Spike_Q954H,N_P13L,NSP3_L1266I,N_R32del,M_Q19E,Spike_N440K,NSP4_T492I,NSP6_L105del,Spike_N679K,Spike_N764K,Spike_L212I,NSP6_G107del,Spike_R346K,NSP6_I189V,Spike_T547K,M_D3G,Spike_D796Y,N_G204R,Spike_V143del,M_A63T,Spike_K417N,NSP6_S106del,Spike_S371L,Spike_G339D,NSP3_S1265del,NSP14_I42V,Spike_P681H,Spike_Y144del,Spike_ins214EPE,N_S33del,Spike_S375F,Spike_G446S,N_E31del,NSP3_K38R,Spike_N211del,E_T9I,Spike_V70del,Spike_L981F,NSP12_P323L,Spike_Y145del,Spike_D614G)
EPI_ISL_9596677	31/01/2022	BA.1	BA.1.15	GRA	(NSP5_P132H,Spike_H69del,Spike_T95I,Spike_A67V,Spike_S373P,Spike_N969K,Spike_H655Y,Spike_N856K,N_R203K,Spike_G142D,NSP3_A1892T,Spike_Q954H,NS3_L106F,N_P13L,NSP3_L1266I,N_R32del,M_Q19E,Spike_N440K,NSP4_T492I,NSP6_L105del,Spike_N679K,Spike_N764K,Spike_L212I,NSP6_G107del,NSP6_I189V,Spike_T547K,M_D3G,Spike_D796Y,N_G204R,N_D343G,Spike_V143del,M_A63T,Spike_K417N,NSP6_S106del,Spike_S371L,Spike_G339D,Spike_P1162S,NSP3_S1265del,NSP14_I42V,Spike_P681H,Spike_Y144del,Spike_ins214EPE,N_S33del,Spike_S375F,Spike_G446S,N_E31del,NSP3_K38R,Spike_N211del,E_T9I,Spike_V70del,Spike_L981F,NSP12_P323L,Spike_Y145del,Spike_D614G)
EPI_ISL_9596699	31/01/2022	BA.1	BA.1.21.1	GRA	(NSP5_P132H,Spike_H69del,Spike_T95I,Spike_A67V,NS7b_E3stop,Spike_S373P,Spike_N969K,Spike_H655Y,Spike_N856K,N_R203K,Spike_G142D,NSP3_A1892T,Spike_Q954H,N_P13L,NSP3_L1266I,N_R32del,M_Q19E,Spike_N440K,NSP4_T492I,NSP6_L105del,Spike_N679K,Spike_N764K,Spike_L212I,NSP6_G107del,NSP6_I189V,Spike_T547K,M_D3G,Spike_D796Y,N_G204R,Spike_V143del,M_A63T,Spike_K417N,NSP6_S106del,Spike_S371L,Spike_G339D,NSP3_S1265del,NSP14_I42V,Spike_P681H,Spike_Y144del,Spike_ins214EPE,N_S33del,Spike_S375F,Spike_G446S,N_E31del,NSP3_K38R,Spike_N211del,E_T9I,Spike_V70del,Spike_L981F,NSP12_P323L,Spike_Y145del,Spike_D614G,NSP12_L749M)
EPI_ISL_9596709	31/01/2022	BA.1	BA.1	GRA	(NSP5_P132H,Spike_H69del,Spike_T95I,Spike_A67V,Spike_S373P,Spike_N969K,Spike_H655Y,Spike_N856K,N_R203K,Spike_G142D,NSP3_A1892T,Spike_Q954H,N_P13L,NSP3_L1266I,N_R32del,M_Q19E,Spike_N440K,NSP4_T492I,NSP6_L105del,Spike_N679K,Spike_N764K,Spike_L212I,NSP6_G107del,NSP6_I189V,Spike_T547K,M_D3G,Spike_D796Y,N_G204R,Spike_V143del,M_A63T,Spike_K417N,NSP6_S106del,Spike_S371L,Spike_G339D,NSP3_S1265del,NSP14_I42V,Spike_P681H,Spike_Y144del,Spike_ins214EPE,N_S33del,Spike_S375F,Spike_G446S,N_E31del,NSP3_K38R,Spike_N211del,E_T9I,Spike_V70del,Spike_L981F,NSP12_P323L,Spike_Y145del,Spike_D614G)
EPI_ISL_9596723	31/01/2022	BA.1	BA.1	GRA	(NSP5_P132H,Spike_H69del,Spike_T95I,Spike_A67V,Spike_S373P,Spike_N969K,Spike_H655Y,Spike_N856K,N_R203K,Spike_G142D,N_P13L,NSP3_L1266I,N_R32del,M_Q19E,Spike_N440K,NSP4_T492I,NSP6_L105del,Spike_N679K,Spike_N764K,Spike_L212I,NSP6_G107del,NSP6_I189V,Spike_T547K,M_D3G,Spike_D796Y,N_G204R,Spike_T478K,Spike_V143del,M_A63T,Spike_S371F,Spike_K417N,NSP6_S106del,Spike_G339D,Spike_R158del,NSP3_S1265del,NSP14_I42V,Spike_P681H,Spike_Y144del,Spike_ins214EPE,Spike_F157del,N_S33del,Spike_S375F,Spike_G446S,N_E31del,Spike_E156G,NSP3_K38R,Spike_N211del,E_T9I,Spike_V70del,Spike_L981F,NSP12_P323L,Spike_Y145del,Spike_D614G,Spike_L452R)
EPI_ISL_9596733	31/01/2022	BA.1	BA.1	GRA	(NSP5_P132H,Spike_H69del,Spike_T95I,Spike_A67V,Spike_S373P,Spike_N969K,Spike_H655Y,Spike_N856K,N_R203K,Spike_G142D,NSP3_A1892T,N_P13L,NSP3_L1266I,N_R32del,M_Q19E,Spike_N440K,NSP4_T492I,NSP6_L105del,Spike_N679K,Spike_N764K,Spike_L212I,NSP6_G107del,NSP6_I189V,Spike_T547K,M_D3G,N_G204R,Spike_T478K,Spike_V143del,M_A63T,Spike_K417N,NSP6_S106del,Spike_S371L,Spike_G339D,Spike_R158del,NSP3_S1265del,NSP14_I42V,Spike_P681H,Spike_Y144del,Spike_ins214EPE,Spike_F157del,N_S33del,Spike_S375F,Spike_G446S,N_E31del,Spike_E156G,NSP3_K38R,Spike_N211del,E_T9I,Spike_V70del,Spike_L981F,NSP12_P323L,Spike_Y145del,Spike_D614G,Spike_L452R)
EPI_ISL_11149372	09/02/2022	BA.1	BA.1.17	GRA	(NSP5_P132H,Spike_H69del,Spike_T95I,Spike_A67V,Spike_S373P,Spike_N969K,Spike_H655Y,Spike_N856K,N_R203K,Spike_G142D,NSP3_A1892T,Spike_Q954H,N_P13L,NSP3_L1266I,N_R32del,M_Q19E,Spike_N440K,NSP4_T492I,NSP6_L105del,Spike_N679K,Spike_N764K,Spike_L212I,NSP6_G107del,NSP6_I189V,Spike_T547K,M_D3G,Spike_D796Y,N_G204R,Spike_V143del,M_A63T,NSP3_P985S,NSP3_V1069I,Spike_K417N,NSP6_S106del,Spike_S371L,Spike_G339D,NSP3_S1265del,NSP14_I42V,Spike_P681H,Spike_Y144del,Spike_ins214EPE,N_S33del,Spike_S375F,Spike_G446S,N_E31del,NSP3_K38R,Spike_N211del,E_T9I,Spike_V70del,Spike_L981F,NSP12_P323L,Spike_Y145del,Spike_D614G)
EPI_ISL_11149534	10/02/2022	BA.1	BA.1.1	GRA	(NSP5_P132H,Spike_H69del,Spike_T95I,Spike_A67V,Spike_S373P,Spike_N969K,Spike_H655Y,Spike_N856K,N_R203K,Spike_G142D,NSP3_A1892T,Spike_Q954H,N_P13L,NSP3_L1266I,N_R32del,M_Q19E,Spike_N440K,NSP4_T492I,NSP6_L105del,Spike_N679K,Spike_N764K,Spike_L212I,NSP6_G107del,Spike_R346K,NSP6_I189V,Spike_T547K,M_D3G,Spike_D796Y,N_G204R,Spike_T478K,Spike_V143del,M_A63T,Spike_K417N,NSP6_S106del,Spike_S371L,Spike_G339D,NSP3_S1265del,NSP14_I42V,Spike_P681H,Spike_Y144del,Spike_ins214EPE,N_S33del,Spike_S375F,Spike_G446S,N_E31del,NSP3_K38R,Spike_N211del,Spike_P1162L,E_T9I,Spike_V70del,Spike_L981F,NSP12_P323L,Spike_Y145del,Spike_D614G)
EPI_ISL_11149538	14/02/2022	BA.1	BA.1	GRA	(NSP5_P132H,Spike_H69del,Spike_T95I,Spike_A67V,Spike_K417N,NSP6_S106del,Spike_N969K,Spike_H655Y,Spike_N856K,N_R203K,Spike_G142D,NSP3_A1892T,Spike_G339D,N_P13L,NSP3_L1266I,N_R32del,NSP3_S1265del,NSP4_P123S,NSP14_I42V,Spike_P681H,Spike_Y144del,Spike_ins214EPE,M_Q19E,N_S33del,NSP4_T492I,NSP6_L105del,Spike_N679K,Spike_N764K,Spike_L212I,NSP6_G107del,N_E31del,NSP3_K38R,Spike_N211del,NSP6_I189V,Spike_T547K,M_D3G,N_G204R,NSP3_T787I,E_T9I,Spike_V70del,Spike_L981F,Spike_V143del,M_A63T,NSP12_P323L,Spike_Y145del,Spike_D614G,NSP4_S127G)
EPI_ISL_11149540	14/02/2022	BA.3	BA.2	GRA	(NSP5_P132H,NSP3_G489S,Spike_L24del,NSP4_T327I,Spike_S373P,Spike_Q493R,Spike_N969K,Spike_H655Y,N_R203K,Spike_V213G,Spike_G142D,Spike_A27S,Spike_Q954H,N_P13L,Spike_N501Y,Spike_P25del,N_R32del,NS3_T223I,Spike_T19I,M_Q19E,Spike_N440K,NSP4_T492I,Spike_N679K,Spike_N764K,NSP6_G107del,Spike_Y505H,Spike_D796Y,N_G204R,Spike_T478K,N_S413R,M_A63T,Spike_S371F,Spike_K417N,NSP13_R392C,Spike_T376A,NSP6_S106del,Spike_G339D,Spike_R408S,NSP4_L438F,NSP14_I42V,NSP4_L264F,Spike_P681H,NSP3_T24I,N_S33del,NSP1_S135R,Spike_S375F,Spike_D405N,Spike_Q498R,Spike_S477N,N_E31del,NSP15_T112I,NSP6_F108del,Spike_E484A,E_T9I,Spike_P26del,NSP12_P323L,Spike_D614G)
EPI_ISL_11149560	14/02/2022	BA.1	BA.1	GRA	(NSP5_P132H,Spike_H69del,Spike_T95I,Spike_A67V,Spike_S373P,Spike_N969K,Spike_H655Y,Spike_N856K,N_R203K,Spike_G142D,NSP3_A1892T,Spike_Q954H,N_P13L,NSP3_L1266I,N_R32del,M_Q19E,Spike_N440K,NSP4_T492I,NSP6_L105del,Spike_N679K,Spike_N764K,Spike_L212I,NSP6_G107del,Spike_R346K,NSP6_I189V,Spike_T547K,M_D3G,Spike_D796Y,N_G204R,Spike_V143del,M_A63T,N_A336V,Spike_K417N,NSP6_S106del,Spike_S371L,Spike_G339D,NSP3_S1265del,NSP14_I42V,Spike_P681H,Spike_Y144del,Spike_ins214EPE,N_S33del,Spike_S375F,Spike_G446S,N_E31del,NSP3_K38R,Spike_N211del,E_T9I,Spike_V70del,Spike_L981F,NSP12_P323L,Spike_Y145del,Spike_D614G)

EPI_ISL_11149563	15/02/2022	BA.1	BA.1.1	GRA	(NSP5_P132H,Spike_H69del,Spike_T95I,Spike_A67V,Spike_S373P,Spike_N969K,Spike_H655Y,Spike_N856K,N_R203K,Spike_G142D,NSP3_V1229A,NSP3_A1892T,NSP3_E1245K,Spike_Q954H,N_P13L,NSP3_L1266I,N_R32del,M_Q19E,Spike_N440K,NSP4_T492I,NSP6_L105del,Spike_N679K,Spike_N764K,Spike_L212I,NSP6_G107del,Spike_R346K,NSP6_I189V,Spike_T547K,M_D3G,Spike_D796Y,N_G204R,Spike_V143del,M_A63T,NSP3_G334S,Spike_K417N,NSP6_S106del,Spike_S371L,Spike_G339D,NSP3_S1265del,NSP14_I42V,Spike_P681H,Spike_Y144del,Spike_ins214EPE,N_S33del,Spike_S375F,Spike_G446S,N_E31del,NSP3_K38R,Spike_N211del,E_T9I,Spike_V70del,Spike_L981F,NSP12_P323L,Spike_Y145del,Spike_D614G)
EPI_ISL_11149616	16/02/2022	BA.1	BA.1.1	GRA	(NSP5_P132H,Spike_H69del,Spike_T95I,NSP6_P44S,Spike_A67V,Spike_S373P,Spike_N969K,Spike_H655Y,Spike_N856K,N_R203K,Spike_G142D,NSP3_A1892T,Spike_Q954H,N_P13L,NSP3_L1266I,N_R32del,M_Q19E,Spike_N440K,NSP4_T492I,NSP6_L105del,Spike_N679K,Spike_N764K,Spike_L212I,NSP6_G107del,Spike_R346K,NSP6_I189V,Spike_T547K,M_D3G,Spike_D796Y,N_G204R,Spike_V143del,M_A63T,NS7a_T39I,Spike_K417N,NSP6_S106del,Spike_S371L,Spike_G339D,NSP3_S1265del,NSP14_I42V,Spike_P681H,Spike_Y144del,Spike_ins214EPE,N_S33del,Spike_S375F,Spike_G446S,N_E31del,NSP3_K38R,Spike_N211del,E_T9I,Spike_V70del,Spike_L981F,NSP12_P323L,Spike_Y145del,Spike_D614G)
EPI_ISL_11149638	16/02/2022	BA.1	BA.1	GRA	(NSP5_P132H,Spike_H69del,Spike_T95I,Spike_A67V,Spike_S373P,Spike_Q493R,Spike_N969K,Spike_H655Y,Spike_N856K,N_R203K,Spike_G142D,NSP3_A1892T,Spike_Q954H,N_P13L,NSP3_L1266I,Spike_N501Y,N_R32del,M_Q19E,Spike_N440K,NSP4_T492I,NSP6_L105del,Spike_N679K,Spike_N764K,Spike_L212I,NSP6_G107del,Spike_Y505H,NSP13_A237T,NSP6_I189V,Spike_T547K,M_D3G,Spike_D796Y,N_G204R,Spike_V143del,M_A63T,Spike_G496S,Spike_K417N,NSP6_S106del,Spike_S371L,Spike_G339D,NSP3_S1265del,NSP14_I42V,Spike_P681H,Spike_Y144del,Spike_ins214EPE,N_S33del,Spike_S375F,Spike_Q498R,Spike_G446S,N_E31del,NSP3_K38R,Spike_N211del,Spike_E484A,E_T9I,Spike_V70del,Spike_L981F,NSP12_P323L,Spike_D614G,Spike_Y145del)
EPI_ISL_11149642	16/02/2022	BA.1	BA.1	GRA	(NSP5_P132H,Spike_H69del,Spike_T95I,Spike_A67V,Spike_K417N,Spike_S373P,NSP6_S106del,Spike_H655Y,Spike_N856K,N_R203K,Spike_S371L,Spike_G142D,NSP3_A1892T,N_P13L,NSP3_L1266I,N_R32del,NSP3_S1265del,NSP14_I42V,Spike_P681H,Spike_Y144del,M_Q19E,Spike_N440K,N_S33del,NSP4_T492I,Spike_S375F,NSP6_T103A,NSP6_L105del,Spike_G446S,Spike_N679K,NSP6_G107del,N_E31del,NSP3_K38R,NSP6_I189V,Spike_T547K,M_D3G,N_G204R,E_T9I,Spike_V70del,Spike_V143del,Spike_V597A,NSP12_P323L,Spike_Y145del,Spike_D614G)
EPI_ISL_11149738	16/02/2022	BA.1	BA.1.1	GRA	(NSP5_P132H,Spike_H69del,Spike_T95I,Spike_A67V,Spike_S373P,Spike_N969K,Spike_H655Y,Spike_N856K,N_R203K,Spike_G142D,NSP3_A1892T,Spike_Q954H,N_P13L,NSP3_L1266I,N_R32del,M_Q19E,Spike_N440K,NSP4_T492I,NSP6_L105del,Spike_N679K,Spike_N764K,Spike_L212I,NSP6_G107del,Spike_R346K,NSP6_I189V,Spike_T547K,M_D3G,Spike_D796Y,N_G204R,Spike_V143del,M_A63T,Spike_K417N,NSP6_S106del,Spike_S371L,Spike_G339D,NSP3_S1265del,NSP14_I42V,Spike_P681H,Spike_Y144del,Spike_ins214EPE,N_S33del,Spike_S375F,Spike_G446S,N_E31del,NSP3_K38R,Spike_N211del,E_T9I,Spike_V70del,Spike_L981F,NSP12_P323L,Spike_Y145del,Spike_D614G)
EPI_ISL_11149740	20/02/2022	BA.1	BA.1	GRA	(NSP5_P132H,N_D63G,Spike_K417N,Spike_H655Y,Spike_N856K,N_R203K,Spike_G142D,NSP14_I42V,Spike_P681H,NSP1_N178D,Spike_Y144del,NSP4_T492I,M_I82T,Spike_D950N,Spike_N679K,NSP3_K38R,NSP6_I189V,NSP13_N503S,Spike_T547K,Spike_D796Y,N_G204R,N_D343G,Spike_T478K,E_T9I,Spike_V143del,NSP12_P323L,Spike_Y145del,Spike_D614G,Spike_F1156L,Spike_L452R,NSP1_V121F,NSP12_P612L)
EPI_ISL_11150082	20/02/2022	BA.1	BA.1	GRA	(NSP5_P132H,NSP12_G671S,Spike_K417N,Spike_H655Y,N_R203K,Spike_G142D,Spike_G339D,N_P13L,N_R32del,NSP14_I42V,Spike_P681H,Spike_Y144del,M_Q19E,NSP3_A488S,Spike_N440K,N_S33del,NSP5_R217K,Spike_G446S,Spike_N679K,Spike_N764K,NSP15_G169E,N_E31del,Spike_R346K,NSP6_I189V,M_D3G,N_G204R,E_T9I,NSP12_V42A,Spike_V143del,NSP12_V667I,M_A63T,Spike_Y145del,Spike_D614G,NS7a_S98F)
EPI_ISL_11151276	20/02/2022	BA.1	BA.1.1	GRA	(NSP5_P132H,Spike_H69del,Spike_T95I,Spike_A67V,Spike_S373P,Spike_N969K,Spike_H655Y,Spike_N856K,N_R203K,Spike_G142D,NSP3_A1892T,Spike_Q954H,N_P13L,NSP3_L1266I,N_R32del,M_Q19E,Spike_N440K,NSP4_T492I,NSP6_L105del,Spike_N679K,Spike_N764K,Spike_L212I,NSP6_G107del,Spike_R346K,NSP6_I189V,Spike_T547K,M_D3G,Spike_D796Y,N_G204R,Spike_V143del,M_A63T,Spike_K417N,NSP6_S106del,Spike_S371L,Spike_G339D,NSP3_S1265del,NSP14_I42V,Spike_P681H,Spike_Y144del,Spike_ins214EPE,N_S33del,Spike_S375F,Spike_G446S,N_E31del,NSP3_K38R,Spike_N211del,E_T9I,Spike_V70del,Spike_L981F,NSP12_P323L,Spike_Y145del,Spike_D614G)
EPI_ISL_11222945	20/02/2022	BA.1	BA.1.17.2	GRA	(NSP5_P132H,Spike_H69del,Spike_T95I,Spike_A67V,Spike_S373P,Spike_N969K,Spike_H655Y,Spike_N856K,N_R203K,Spike_G142D,NSP3_A1892T,Spike_Q954H,Spike_A701V,N_P13L,NSP3_L1266I,N_R32del,M_Q19E,Spike_N440K,NSP4_T492I,NSP6_L105del,Spike_N679K,Spike_N764K,Spike_L212I,NSP6_G107del,NSP6_I189V,Spike_T547K,M_D3G,Spike_D796Y,N_G204R,Spike_V143del,M_A63T,NSP3_V1069I,Spike_K417N,NSP6_S106del,Spike_S371L,Spike_G339D,NSP3_S1265del,NSP14_I42V,Spike_P681H,Spike_Y144del,Spike_ins214EPE,N_S33del,Spike_S375F,Spike_G446S,N_E31del,NSP3_K38R,Spike_N211del,E_T9I,Spike_V70del,Spike_L981F,NSP12_P323L,Spike_Y145del,Spike_D614G)
EPI_ISL_11223274	21/02/2022	BA.1	BA.1.17	GRA	(NSP5_P132H,Spike_H69del,Spike_T95I,Spike_A67V,Spike_S373P,Spike_Q493R,Spike_N969K,Spike_H655Y,Spike_N856K,N_R203K,Spike_G142D,NSP3_A1892T,Spike_Q954H,N_P13L,NSP3_L1266I,Spike_N501Y,N_R32del,M_Q19E,Spike_N440K,NSP4_T492I,NSP6_L105del,Spike_N679K,Spike_N764K,Spike_L212I,NSP6_G107del,Spike_Y505H,NSP6_I189V,Spike_T547K,M_D3G,Spike_D796Y,N_G204R,Spike_V143del,M_A63T,Spike_G496S,NSP3_P985S,NSP3_V1069I,Spike_K417N,NSP6_S106del,Spike_S371L,Spike_G339D,NSP3_S1265del,NSP14_I42V,Spike_P681H,Spike_Y144del,Spike_ins214EPE,N_S33del,Spike_S375F,N_A125V,Spike_Q498R,Spike_G446S,N_E31del,NSP3_K38R,Spike_N211del,Spike_E484A,E_T9I,Spike_V70del,Spike_L981F,NSP12_P323L,Spike_D614G,Spike_Y145del)
EPI_ISL_11224100	21/02/2022	BA.1	BA.1	GRA	(NSP5_P132H,Spike_H69del,Spike_T95I,Spike_A67V,Spike_S373P,Spike_N969K,Spike_H655Y,Spike_N856K,N_R203K,Spike_G142D,NSP3_A1892T,Spike_Q954H,N_P13L,NSP3_L1266I,N_R32del,M_Q19E,Spike_N440K,NSP4_T492I,NSP6_L105del,Spike_N679K,Spike_N764K,Spike_L212I,NSP6_G107del,NSP6_I189V,Spike_T547K,M_D3G,Spike_D796Y,N_G204R,Spike_T478K,Spike_V143del,M_A63T,N_T379N,Spike_K417N,NSP6_S106del,Spike_S371L,Spike_G339D,NSP3_S1265del,NSP14_I42V,Spike_P681H,Spike_Y144del,Spike_ins214EPE,N_S33del,Spike_S375F,Spike_G446S,N_E31del,NSP3_K38R,Spike_N211del,E_T9I,Spike_V70del,Spike_L981F,NSP12_P323L,Spike_Y145del,Spike_D614G)
EPI_ISL_11224711	21/02/2022	BA.1	BA.1.1	GRA	(NSP5_P132H,Spike_H69del,Spike_T95I,Spike_A67V,Spike_S373P,Spike_Q493R,Spike_N969K,Spike_H655Y,Spike_N856K,N_R203K,Spike_G142D,NSP3_A1892T,Spike_Q954H,N_P13L,NSP3_L1266I,Spike_N501Y,N_R32del,M_Q19E,Spike_N440K,NSP4_T492I,NSP6_L105del,Spike_N679K,Spike_N764K,Spike_L212I,NSP6_G107del,Spike_Y505H,Spike_R346K,NSP6_I189V,Spike_T547K,M_D3G,Spike_D796Y,N_G204R,Spike_V143del,M_A63T,Spike_G496S,Spike_K417N,NSP6_S106del,Spike_S371L,Spike_

					G339D,NSP3_S1265del,NSP14_I42V,Spike_P681H,Spike_Y144del,Spike_ins214EPE,N_S33del,Spike_S375F,Spike_Q498R,Spike_G446S,N_E31del,NSP3_K38R,Spike_N211del,Spike_E484A,E_T9I,Spike_V70del,Spike_L981F,NSP12_P323L,Spike_D614G,Spike_Y145del)
EPI_ISL_12030822	21/02/2022	BA.1	BA.1.1	GRA	(NSP5_P132H,Spike_H69del,Spike_T95I,Spike_A67V,Spike_S373P,Spike_N969K,Spike_H655Y,Spike_N856K,N_R203K,Spike_G142D,NSP3_A1892T,Spike_Q954H,N_P13L,N_R32del,M_Q19E,Spike_N440K,NSP4_T492I,NSP6_L105del,Spike_N679K,Spike_N764K,Spike_L212I,NSP6_G107del,Spike_R346K,NSP6_I189V,Spike_T547K,M_D3G,Spike_D796Y,N_G204R,Spike_V143del,M_A63T,Spike_K417N,NSP6_S106del,Spike_S371L,Spike_G339D,NSP14_I42V,Spike_P681H,Spike_Y144del,Spike_ins214EPE,N_S33del,Spike_S375F,Spike_G446S,N_E31del,NSP3_K38R,Spike_N211del,E_T9I,Spike_V70del,Spike_L981F,NSP12_P323L,Spike_Y145del,Spike_D614G)
EPI_ISL_12031068	24/02/2022	BA.2	BA.2	GRA	(NSP5_P132H,NSP3_G489S,Spike_L24del,NSP4_T327I,Spike_S373P,Spike_Q493R,Spike_N969K,Spike_H655Y,N_R203K,Spike_V213G,Spike_G142D,Spike_A27S,Spike_Q954H,N_P13L,Spike_P25del,N_R32del,NS3_T223I,Spike_T19I,M_Q19E,Spike_N440K,NSP4_T492I,Spike_N679K,Spike_N764K,NSP6_G107del,Spike_D796Y,N_G204R,Spike_T478K,N_S413R,M_A63T,Spike_S371F,Spike_K417N,NSP13_R392C,Spike_T376A,NSP6_S106del,Spike_G339D,Spike_R408S,NSP4_L438F,NSP14_I42V,NSP4_L264F,Spike_P681H,NSP3_T24I,N_S33del,NSP1_S135R,Spike_S375F,Spike_D405N,Spike_S477N,N_E31del,NSP15_T112I,NSP6_F108del,Spike_E484A,E_T9I,Spike_P26del,NSP12_P323L,Spike_D614G)
EPI_ISL_12032292	24/02/2022	BA.1	BA.1.1	GRA	(NSP5_P132H,Spike_H69del,Spike_T95I,Spike_A67V,Spike_S373P,Spike_N969K,Spike_H655Y,Spike_N856K,N_R203K,Spike_G142D,NSP3_A1892T,Spike_Q954H,N_P13L,NSP3_L1266I,N_R32del,M_Q19E,Spike_N440K,NSP4_T492I,NSP6_L105del,Spike_N679K,Spike_N764K,Spike_L212I,NSP6_G107del,Spike_R346K,NSP6_I189V,Spike_T547K,M_D3G,Spike_D796Y,N_G204R,Spike_V143del,M_A63T,N_L230F,Spike_K417N,NSP6_S106del,Spike_S371L,Spike_G339D,NSP3_S1265del,NSP14_I42V,Spike_P681H,Spike_Y144del,Spike_ins214EPE,NSP2_D315N,N_S33del,Spike_S375F,Spike_G446S,N_E31del,NSP3_K38R,Spike_N211del,Spike_Q628K,E_T9I,Spike_V70del,Spike_L981F,NSP12_P323L,Spike_Y145del,Spike_D614G)
EPI_ISL_12031318	27/02/2022	BA.1	BA.1	GRA	(NSP5_P132H,Spike_H69del,Spike_T95I,Spike_A67V,Spike_S373P,Spike_N969K,Spike_H655Y,Spike_N856K,N_R203K,Spike_G142D,NSP3_A1892T,Spike_Q954H,N_P13L,NSP3_L1266I,N_R32del,M_Q19E,Spike_N440K,NSP4_T492I,NSP6_L105del,Spike_N679K,Spike_N764K,Spike_L212I,NSP6_G107del,NSP6_I189V,Spike_T547K,M_D3G,N_G204R,Spike_V143del,M_A63T,Spike_K417N,NSP6_S106del,Spike_S371L,NSP3_S1265del,NSP14_I42V,Spike_P681H,Spike_Y144del,Spike_ins214EPE,N_S33del,Spike_S375F,Spike_G446S,N_E31del,NSP3_K38R,Spike_N211del,E_T9I,Spike_V70del,Spike_L981F,NSP9_T67I,NSP12_P323L,Spike_Y145del,Spike_D614G)
EPI_ISL_13566021	27/02/2022	BA.1	BA.1.1	GRA	(NSP5_P132H,Spike_H69del,Spike_T95I,Spike_A67V,Spike_S373P,Spike_Q493R,Spike_N969K,Spike_H655Y,Spike_N856K,N_R203K,Spike_G142D,NSP3_A1892T,Spike_Q954H,N_P13L,NSP3_L1266I,Spike_N501Y,N_R32del,NSP1_K129R,M_Q19E,Spike_N440K,NSP4_T492I,NSP6_L105del,Spike_N679K,Spike_N764K,Spike_L212I,NSP6_G107del,Spike_Y505H,Spike_R346K,NSP6_I189V,Spike_T547K,M_D3G,Spike_D796Y,N_G204R,Spike_V143del,M_A63T,Spike_G496S,Spike_K417N,NSP6_S106del,Spike_S371L,Spike_G339D,NSP3_S1265del,NSP14_I42V,Spike_P681H,Spike_Y144del,Spike_ins214EPE,N_S33del,Spike_S375F,Spike_Q498R,Spike_G446S,N_E31del,NSP3_K38R,Spike_N211del,Spike_E484A,E_T9I,Spike_V70del,Spike_L981F,NSP12_P323L,Spike_D614G,Spike_Y145del)
EPI_ISL_13566022	27/02/2022	BA.1	BA.1	GRA	(NSP5_P132H,Spike_H69del,Spike_T95I,Spike_A67V,Spike_S373P,Spike_N969K,Spike_H655Y,Spike_N856K,N_R203K,Spike_G142D,NSP3_A1892T,Spike_Q954H,N_P13L,NSP3_L1266I,N_R32del,NSP4_P123S,M_Q19E,Spike_N440K,NSP4_T492I,NSP6_L105del,Spike_N679K,Spike_N764K,Spike_L212I,NSP6_G107del,NSP6_I189V,Spike_T547K,M_D3G,Spike_D796Y,N_G204R,Spike_V143del,M_A63T,NSP4_S127G,Spike_K417N,NSP6_S106del,Spike_S371L,Spike_G339D,Spike_R158del,NSP3_S1265del,NSP14_I42V,Spike_P681H,Spike_Y144del,Spike_ins214EPE,Spike_F157del,N_S33del,Spike_S375F,Spike_G446S,N_E31del,Spike_E156G,NSP3_K38R,Spike_N211del,Spike_M153I,E_T9I,Spike_V70del,Spike_L981F,NSP12_P323L,Spike_Y145del,Spike_D614G)
EPI_ISL_13566023	01/03/2022	BA.2	BA.2	GRA	(NSP5_P132H,NSP3_G489S,Spike_L24del,NSP4_T327I,Spike_S373P,Spike_Q493R,Spike_N969K,Spike_H655Y,N_R203K,Spike_V213G,Spike_G142D,Spike_A27S,Spike_Q954H,N_P13L,Spike_N501Y,Spike_P25del,N_R32del,NS3_T223I,Spike_T19I,M_Q19E,Spike_N440K,NSP4_T492I,Spike_N679K,Spike_N764K,NSP6_G107del,Spike_Y505H,Spike_D796Y,N_G204R,N_S413R,M_A63T,Spike_S371F,Spike_K417N,NSP13_R392C,Spike_T376A,NSP6_S106del,Spike_G339D,Spike_R408S,NSP4_L438F,NSP14_I42V,NSP4_L264F,Spike_P681H,NSP3_T24I,N_S33del,NSP1_S135R,Spike_S375F,Spike_D405N,N_E31del,NSP15_T112I,NSP6_F108del,E_T9I,Spike_P26del,NSP12_P323L,Spike_D614G,NSP12_K91E)
EPI_ISL_13566025	01/03/2022	BA.1	BA.1.7	GRA	(NSP5_P132H,Spike_H69del,Spike_T95I,Spike_A67V,Spike_S373P,Spike_N969K,Spike_H655Y,Spike_N856K,N_R203K,Spike_G142D,N_P13L,NSP3_L1266I,N_R32del,M_Q19E,NSP4_T492I,NSP6_L105del,Spike_N679K,Spike_N764K,NSP6_G107del,NSP6_I189V,Spike_T547K,M_D3G,N_G204R,NSP2_F93S,Spike_T478K,Spike_V143del,M_A63T,NS6_L16V,NS7a_S98F,Spike_S371F,NSP3_T1269I,Spike_K417N,NSP6_S106del,Spike_G339D,NSP3_S1265del,NSP14_I42V,Spike_P681H,Spike_Y144del,N_S33del,Spike_S375F,N_E31del,NSP3_K38R,E_T9I,Spike_V70del,Spike_L981F,NSP12_P323L,Spike_Y145del,Spike_D614G)
EPI_ISL_13566026	01/03/2022	BA.1	BA.1	GRA	(NSP5_P132H,Spike_H69del,Spike_T95I,Spike_A67V,Spike_S373P,Spike_N969K,Spike_H655Y,Spike_N856K,N_R203K,Spike_G142D,NSP3_A1892T,Spike_Q954H,N_P13L,NSP3_L1266I,N_R32del,M_Q19E,NSP4_T492I,NSP6_L105del,NSP13_H164Y,Spike_N679K,Spike_N764K,NSP6_G107del,NSP6_I189V,Spike_T547K,M_D3G,N_G204R,Spike_T478K,Spike_V143del,M_A63T,Spike_K417N,NSP6_S106del,Spike_S371L,NSP3_S1265del,NSP14_I42V,Spike_P681H,Spike_Y144del,N_S33del,Spike_S375F,N_E31del,NSP3_K38R,NSP12_F694L,E_T9I,Spike_V70del,Spike_L981F,NSP12_P323L,Spike_Y145del,Spike_D614G,Spike_L452R)
EPI_ISL_13566027	01/03/2022	BA.2	BA.2	GRA	(NSP5_P132H,NSP3_G489S,Spike_L24del,NSP4_T327I,Spike_S373P,Spike_Q493R,Spike_N969K,Spike_H655Y,N_R203K,Spike_V213G,Spike_G142D,Spike_A27S,Spike_Q954H,N_P13L,Spike_N501Y,Spike_P25del,N_R32del,NS3_T223I,NSP13_T440I,Spike_T19I,M_Q19E,Spike_N440K,NSP4_T492I,Spike_N679K,Spike_N764K,NSP6_G107del,Spike_Y505H,Spike_D796Y,N_G204R,Spike_T478K,N_S413R,M_A63T,Spike_S371F,NSP3_V1603F,Spike_K417N,NSP13_R392C,Spike_T376A,NSP6_S106del,Spike_G339D,Spike_R408S,NSP4_L438F,NSP14_I42V,NSP4_L264F,Spike_P681H,NSP3_T24I,N_S33del,NSP1_S135R,Spike_S375F,Spike_D405N,Spike_S477N,N_E31del,NSP15_T112I,NSP6_F108del,Spike_E484A,E_T9I,Spike_P26del,NSP12_P323L,Spike_D614G)
EPI_ISL_13566036	02/03/2022	BA.1	BA.1.1.1	GRA	(NSP5_P132H,Spike_H69del,Spike_T95I,Spike_A67V,Spike_S373P,Spike_Q493R,Spike_N969K,Spike_H655Y,Spike_N856K,N_R203K,Spike_G142D,NSP3_A1892T,Spike_Q954H,N_P13L,NSP3_L1266I,Spike_N501Y,N_R32del,M_Q19E,Spike_N440K,NSP4_T492I,NSP6_L105del,Spike_N679K,Spike_N764K,Spike_L212I,NSP6_G107del,Spike_Y505H,Spike_R346K,NSP6_I189V,Spike_T547K,M_D3G,Spike_D796Y,N_G204R,Spike_V143del,M_A63T,Spike_G496S,NSP12_Q875R,Spike_K417N,NSP6_S106del,Spike_S371L,Spike_G339D,NSP3_S1265del,NSP14_I42V,Spike_P681H,Spike_Y144del,Spike_ins214EPE,N_S33del,Spike_S375F,Spike_Q498R,Spike_G446S,N_E31del,NSP3_K38R,Spike_N211del,Spike_E484A,E_T9I,Spike_V70del,Spike_L981F,NSP12_P323L,Spike_D614G,Spike_Y145del)

EPI_ISL_13566030	04/03/2022	BA.1	BA.1.13	GRA	(NSP5_P132H,Spike_H69del,Spike_T95I,Spike_A67V,NSP2_K384N,Spike_S373P,Spike_N969K,Spike_H655Y,Spike_N856K,N_R203K,Spike_G142D,NSP3_A1892T,Spike_Q954H,N_P13L,NSP3_L1266I,N_R32del,M_Q19E,Spike_N440K,NSP4_T492I,NSP6_L105del,Spike_N679K,Spike_N764K,Spike_L212I,NSP6_G107del,NSP6_I189V,Spike_T547K,M_D3G,Spike_D796Y,N_G204R,Spike_V143del,M_A63T,NSP6_S106del,Spike_S371L,Spike_K417T,N_D348Y,Spike_G339D,NSP15_L162F,NSP3_S1265del,NSP14_I42V,Spike_P681H,Spike_Y144del,Spike_ins214EPE,N_S33del,Spike_S375F,Spike_S691F,Spike_G446S,N_T366I,N_E31del,NSP3_K38R,Spike_N211del,E_T9I,Spike_V70del,Spike_L981F,NSP12_P323L,Spike_Y145del,Spike_D614G)
EPI_ISL_13566032	05/03/2022	BA.1	BA.1.17.2	GRA	(NSP5_P132H,Spike_H69del,Spike_T95I,Spike_A67V,Spike_S373P,Spike_N969K,Spike_H655Y,Spike_N856K,N_R203K,Spike_G142D,NSP3_A1892T,Spike_Q954H,Spike_A701V,N_P13L,NSP2_R218H,NSP3_L1266I,N_R32del,M_Q19E,Spike_N440K,NSP4_T492I,NSP6_L105del,Spike_N679K,Spike_N764K,Spike_L212I,NSP6_G107del,NSP6_I189V,Spike_T547K,M_D3G,Spike_D796Y,N_G204R,Spike_V143del,M_A63T,NSP3_V1069I,Spike_K417N,NSP6_S106del,Spike_S371L,Spike_G339D,NSP3_S1265del,NSP14_I42V,Spike_P681H,Spike_Y144del,Spike_ins214EPE,N_S33del,Spike_S375F,Spike_G446S,N_E31del,NSP3_K38R,Spike_N211del,E_T9I,Spike_V70del,Spike_L981F,NSP12_P323L,Spike_Y145del,Spike_D614G)
EPI_ISL_13566033	05/03/2022	BA.1	BA.1	GRA	(NSP5_P132H,Spike_H69del,Spike_T95I,Spike_A67V,Spike_S373P,Spike_N969K,Spike_H655Y,Spike_N856K,N_R203K,Spike_G142D,NSP3_A1892T,Spike_Q954H,N_P13L,NSP3_L1266I,N_R32del,M_Q19E,Spike_N440K,NSP4_T492I,NSP6_L105del,Spike_N679K,Spike_N764K,Spike_L212I,NSP6_G107del,NSP6_I189V,Spike_T547K,M_D3G,Spike_D796Y,N_G204R,Spike_V143del,M_A63T,Spike_K417N,NSP6_S106del,Spike_S371L,Spike_G339D,NSP15_D39Y,NSP3_S1265del,NSP14_I42V,Spike_P681H,Spike_Y144del,Spike_ins214EPE,N_S33del,Spike_S375F,Spike_G446S,N_E31del,NSP3_K38R,Spike_N211del,E_T9I,Spike_V70del,Spike_L981F,NSP12_P323L,Spike_Y145del,Spike_D614G)
EPI_ISL_11149332	06/03/2022	BA.2	BA.2	GRA	(NSP5_P132H,NSP3_G489S,Spike_L24del,NSP4_T327I,Spike_S373P,Spike_Q493R,Spike_N969K,Spike_H655Y,N_R203K,Spike_V213G,Spike_G142D,Spike_A27S,Spike_Q954H,N_P13L,Spike_N501Y,Spike_P25del,N_R32del,NS3_T223I,Spike_T19I,M_Q19E,Spike_N440K,NSP4_T492I,Spike_N679K,Spike_N764K,NSP6_G107del,Spike_Y505H,Spike_D796Y,N_G204R,N_S413R,M_A63T,Spike_S371F,Spike_K417N,NSP13_R392C,Spike_T376A,NSP6_S106del,Spike_G339D,Spike_R408S,NSP4_L438F,NSP14_I42V,NSP4_L264F,Spike_P681H,NSP3_T24I,N_S33del,NSP1_S135R,Spike_S375F,Spike_D405N,Spike_Q498R,N_E31del,NSP15_T112I,NSP6_F108del,Spike_E484A,E_T9I,Spike_P26del,NSP12_P323L,Spike_D614G)
EPI_ISL_13566035	07/03/2022	BA.1	BA.1.17.2	GRA	(NSP5_P132H,Spike_H69del,Spike_P809S,Spike_T95I,Spike_A67V,Spike_S373P,Spike_N969K,Spike_H655Y,Spike_N856K,N_R203K,Spike_G142D,NSP2_K142R,NSP3_A1892T,Spike_Q954H,Spike_A701V,N_P13L,NSP3_L1266I,N_R32del,M_Q19E,Spike_N440K,NSP4_T492I,NSP6_L105del,Spike_N679K,Spike_N764K,Spike_L212I,NSP6_G107del,NSP6_I189V,Spike_T547K,M_D3G,Spike_D796Y,N_G204R,Spike_V143del,M_A63T,NSP3_V1069I,Spike_K417N,NSP6_S106del,Spike_S371L,Spike_G339D,NSP3_S1265del,NSP14_I42V,Spike_P681H,Spike_Y144del,Spike_ins214EPE,N_S33del,Spike_S375F,Spike_G446S,N_E31del,NSP3_K38R,Spike_N211del,E_T9I,Spike_V70del,Spike_L981F,NSP12_P323L,Spike_Y145del,Spike_D614G)
EPI_ISL_12280331	01/04/2022	BA.2	BA.2.3.15	GRA	(NSP5_P132H,NSP3_G489S,Spike_L24del,NSP4_T327I,Spike_S373P,Spike_N969K,Spike_H655Y,N_R203K,Spike_V213G,Spike_G142D,Spike_A27S,Spike_Q954H,N_P13L,Spike_P25del,N_R32del,NS3_T223I,Spike_T19I,M_Q19E,Spike_N440K,NSP4_T492I,Spike_N679K,Spike_N764K,NSP6_G107del,N_G204R,M_A63T,Spike_S371F,Spike_K417N,NSP13_R392C,Spike_T376A,NSP6_S106del,Spike_G339D,Spike_R408S,NSP4_L438F,NSP4_A146V,Spike_P681H,NSP4_L264F,NSP3_T24I,N_S33del,NSP1_S135R,Spike_S375F,Spike_D405N,Spike_D215E,N_E31del,NSP15_T112I,NSP6_F108del,E_T9I,Spike_P26del,NSP12_P323L,Spike_D614G)
EPI_ISL_12280333	01/04/2022	BA.2	BA.2	GR	(NSP5_P132H,Spike_S371F,NSP3_G489S,NSP13_R392C,Spike_K417N,Spike_L24del,Spike_S373P,NSP6_S106del,Spike_T376A,Spike_N969K,Spike_H655Y,N_R203K,Spike_V213G,Spike_A27S,Spike_G339D,N_P13L,Spike_R408S,Spike_P25del,N_R32del,NSP4_L438F,NS3_T223I,Spike_T19I,NSP14_I42V,Spike_P681H,NSP4_L264F,M_Q19E,NSP3_T24I,N_S33del,NSP1_S135R,NSP4_T492I,Spike_S375F,Spike_D405N,Spike_N679K,Spike_N764K,NSP6_G107del,N_E31del,NSP15_T112I,NSP6_F108del,N_G204R,E_T9I,N_S413R,Spike_P26del,M_A63T,NSP12_P323L,Spike_D614G)
EPI_ISL_12280332	02/04/2022	BA.2	BA.2.9	GR	(NSP5_P132H,NSP3_G489S,Spike_L24del,NSP4_T327I,Spike_S373P,Spike_N969K,Spike_H655Y,N_R203K,Spike_V213G,Spike_G142D,Spike_A27S,Spike_Q954H,N_P13L,Spike_P25del,N_R32del,NS3_T223I,Spike_T19I,M_Q19E,NSP4_T492I,Spike_N679K,Spike_N764K,NSP6_G107del,N_G204R,M_A63T,Spike_S371F,NSP1_M85del,NS3_H78Y,NSP1_E87K,Spike_K417N,NSP13_R392C,Spike_T376A,NSP6_S106del,Spike_R408S,NSP4_L438F,NSP14_I42V,NSP4_L264F,Spike_P681H,NSP3_T24I,N_S33del,NSP1_S135R,Spike_S375F,Spike_D405N,N_E31del,NSP1_V86del,NSP15_T112I,NSP6_F108del,NSP3_A150D,E_T9I,Spike_P26del,NSP12_P323L,Spike_D614G)
EPI_ISL_12280334	05/04/2022	BA.2	BA.2.9	GRA	(NSP5_P132H,NSP3_G489S,Spike_L24del,NSP4_T327I,Spike_S373P,Spike_N969K,Spike_H655Y,N_R203K,Spike_V213G,Spike_G142D,Spike_A27S,Spike_Q954H,N_P13L,Spike_P25del,N_R32del,NS3_T223I,Spike_T19I,M_Q19E,Spike_N440K,NSP4_T492I,Spike_N679K,Spike_N764K,NSP6_G107del,Spike_P1079S,N_G204R,Spike_T478K,M_A63T,Spike_S371F,NS3_H78Y,Spike_K417N,NSP13_R392C,Spike_T376A,NSP6_S106del,Spike_R408S,NSP4_L438F,NSP14_I42V,Spike_P681H,NSP4_L264F,NSP3_T24I,N_S33del,NSP1_S135R,Spike_S375F,Spike_D405N,Spike_S477N,N_E31del,NSP15_T112I,NSP6_F108del,Spike_E484A,E_T9I,Spike_P26del,NSP12_P323L,Spike_D614G)
EPI_ISL_12280343	05/04/2022	BA.2	BA.2	GR	(NSP5_P132H,NSP3_G489S,Spike_L24del,NSP4_T327I,Spike_S373P,Spike_N969K,Spike_H655Y,N_R203K,Spike_V213G,Spike_G142D,Spike_A27S,Spike_Q954H,N_P13L,Spike_P25del,N_R32del,NS3_T223I,Spike_T19I,M_Q19E,Spike_N440K,NSP4_T492I,Spike_N679K,Spike_N764K,NSP6_G107del,N_G204R,N_S413R,M_A63T,Spike_S371F,Spike_K417N,NSP13_R392C,Spike_T376A,NSP6_S106del,Spike_R408S,NSP4_L438F,Spike_P681H,NSP4_L264F,NSP3_T24I,N_S33del,NSP1_S135R,Spike_S375F,Spike_D405N,Spike_M153V,N_E31del,NSP6_F108del,E_T9I,NSP15_V22L,Spike_P26del,NSP12_P323L,Spike_D614G)
EPI_ISL_12280335	07/04/2022	BA.2	BA.2	GR	(NSP5_P132H,NSP3_G489S,Spike_L24del,NSP4_T327I,Spike_S373P,Spike_N969K,Spike_H655Y,N_R203K,Spike_V213G,Spike_G142D,Spike_A27S,Spike_Q954H,N_P13L,Spike_P25del,N_R32del,NS3_T223I,Spike_T19I,M_Q19E,Spike_N440K,NSP4_T492I,Spike_N679K,Spike_N764K,NSP6_G107del,N_G204R,M_A63T,Spike_S371F,Spike_K417N,NSP13_R392C,Spike_T376A,NSP6_S106del,Spike_R408S,NSP4_L438F,NSP14_I42V,Spike_P681H,NSP4_L264F,NSP3_T24I,N_S33del,NSP1_S135R,Spike_S375F,Spike_D405N,N_E31del,NSP15_T112I,NSP6_F108del,NSP2_P568L,E_T9I,Spike_P26del,NSP12_P323L,Spike_D614G)
EPI_ISL_12280336	07/04/2022	BA.2	BA.2	GRA	(NSP5_P132H,NSP3_G489S,Spike_L24del,NSP4_T327I,Spike_S373P,Spike_N969K,Spike_H655Y,N_R203K,Spike_V213G,Spike_G142D,Spike_A27S,Spike_Q954H,N_P13L,Spike_P25del,N_R32del,NS3_T223I,Spike_T19I,M_Q19E,Spike_N440K,NSP4_T492I,Spike_N679K,Spike_N764K,NSP6_G107del,N_G204R,Spike_T478K,M_A63T,Spike_S371F,Spike_K417N,NSP13_R392C,Spike_T376A,NSP6_S106del,Spike_R408S,NSP4_L438F,NSP14_I42V,Spike_P681H,NSP4_L264F,NSP3_T24I,N_S33del,NSP1_S135R,Spike_S375F,Spike_D405N,Spike_S477N,N_E31del,NSP15_T112I,NSP6_F108del,Spike_E484A,Spike_P26del,NSP12_P323L,Spike_D614G)

EPI_ISL_12280337	07/04/2022	BA.2	BA.2.10	GRA	(NSP5_P132H,NSP3_G489S,Spike_L24del,Spike_S373P,NSP4_T327I,Spike_N969K,Spike_H655Y,N_R203K,Spike_V213G,Spike_G142D,Spike_A27S,Spike_Q954H,N_P13L,Spike_P25del,N_R32del,NS3_T223I,Spike_T19I,M_Q19E,Spike_N440K,NSP4_T492I,Spike_N679K,Spike_N764K,NSP6_G107del,N_G204R,Spike_T478K,M_A63T,Spike_S371F,Spike_K417N,NSP13_R392C,NS3_S216L,Spike_T376A,NSP6_S106del,Spike_R408S,NSP4_L438F,NSP14_I42V,Spike_P681H,NSP4_L264F,NSP3_T24I,N_S33del,NSP1_S135R,Spike_S375F,Spike_D405N,NSP13_S36P,Spike_S477N,N_E31del,NSP15_T112I,NSP6_F108del,Spike_E484A,Spike_P26del,NSP12_P323L,Spike_D614G)
EPI_ISL_12280338	07/04/2022	BA.2	BA.2	GRA	(NSP5_P132H,NSP3_G489S,Spike_L24del,NSP4_T327I,Spike_S373P,Spike_N969K,Spike_H655Y,N_R203K,Spike_V213G,Spike_G142D,Spike_A27S,Spike_Q954H,N_P13L,Spike_P25del,N_R32del,NS3_T223I,Spike_T19I,M_Q19E,Spike_N440K,NSP4_T492I,Spike_N679K,Spike_N764K,NSP6_G107del,N_G204R,M_A63T,Spike_S371F,Spike_K417N,NSP13_R392C,Spike_T376A,NSP6_S106del,Spike_G339D,Spike_R408S,NSP4_L438F,NSP4_L264F,Spike_P681H,NSP3_T24I,N_S33del,NSP1_S135R,Spike_S375F,Spike_D405N,NSP3_L75M,N_E31del,NSP15_T112I,NSP6_F108del,E_T9I,Spike_P26del,NSP12_P323L)
EPI_ISL_12280345	08/04/2022	BA.2	BA.2	GRA	(NSP5_P132H,Spike_S371F,NSP3_G489S,NSP13_R392C,Spike_K417N,Spike_L24del,NSP4_T327I,Spike_S373P,NSP6_S106del,Spike_T376A,Spike_N969K,Spike_H655Y,N_R203K,Spike_V213G,Spike_G142D,Spike_A27S,Spike_Q954H,Spike_G339D,N_P13L,Spike_R408S,Spike_P25del,N_R32del,NSP4_L438F,NS3_T223I,Spike_T19I,Spike_P681H,NSP4_L264F,M_Q19E,NSP3_T24I,Spike_N440K,N_S33del,NSP1_S135R,NSP4_T492I,Spike_S375F,Spike_D405N,Spike_N679K,Spike_N764K,NSP6_G107del,N_E31del,NSP15_T112I,NSP6_F108del,N_G204R,Spike_P26del,M_A63T,NSP12_P323L,Spike_D614G)
EPI_ISL_12280319	11/04/2022	BA.2	BA.2	GRA	(NSP5_P132H,NSP3_G489S,Spike_L24del,NSP4_T327I,Spike_S373P,Spike_N969K,Spike_H655Y,N_R203K,Spike_V213G,Spike_A27S,Spike_Q954H,N_P13L,Spike_P25del,N_R32del,NS3_T223I,Spike_T19I,M_Q19E,Spike_N440K,NSP4_T492I,Spike_N679K,Spike_N764K,NSP6_G107del,N_G204R,Spike_T478K,N_S413R,M_A63T,NSP12_I223M,Spike_S371F,Spike_K417N,NSP13_R392C,Spike_T376A,NSP6_S106del,Spike_G339D,Spike_R408S,NSP4_L438F,NSP4_L264F,Spike_P681H,N_S33del,NSP1_S135R,Spike_S375F,Spike_D405N,Spike_S477N,N_E31del,NSP15_T112I,NSP6_F108del,Spike_E484A,Spike_P26del,NSP12_P323L)
EPI_ISL_12280326	11/04/2022	BA.2	BA.2.9	GRA	(NSP5_P132H,NSP3_G489S,Spike_L24del,NSP4_T327I,Spike_S373P,Spike_N969K,Spike_H655Y,N_R203K,Spike_V213G,Spike_G142D,Spike_A27S,Spike_Q954H,N_P13L,Spike_P25del,N_R32del,NS3_T223I,Spike_T19I,M_Q19E,NSP4_T492I,Spike_N679K,Spike_N764K,NSP6_G107del,N_G204R,N_S413R,NSP14_A353V,M_A63T,Spike_S371F,NS3_H78Y,Spike_K417N,NSP13_R392C,Spike_T376A,NSP6_S106del,Spike_G339D,Spike_R408S,NSP4_L438F,NSP14_I42V,NSP4_L264F,Spike_P681H,NSP3_T24I,N_S33del,NSP1_S135R,Spike_S375F,Spike_D405N,NSP2_L372F,N_E31del,NSP15_T112I,NSP6_F108del,E_T9I,Spike_P26del,NSP12_P323L,Spike_D614G)
EPI_ISL_12280339	11/04/2022	BA.2	BA.2.9	GRA	(NSP5_P132H,NSP3_G489S,Spike_L24del,NSP4_T327I,Spike_S373P,Spike_N969K,Spike_H655Y,N_R203K,Spike_V213G,Spike_G142D,Spike_A27S,Spike_Q954H,N_P13L,Spike_P25del,N_R32del,NS3_T223I,Spike_T19I,M_Q19E,Spike_N440K,NSP4_T492I,Spike_N679K,Spike_N764K,NSP6_G107del,N_G204R,M_A63T,Spike_S371F,NS3_H78Y,Spike_K417N,NSP13_R392C,Spike_T376A,NSP6_S106del,Spike_G339D,Spike_R408S,NSP4_L438F,Spike_P681H,NSP4_L264F,NSP3_T24I,N_S33del,NSP1_S135R,Spike_S375F,Spike_D405N,N_E31del,NSP15_T112I,NSP4_S395G,NSP6_F108del,E_T9I,Spike_P26del,NSP12_P323L,Spike_D614G)
EPI_ISL_12280341	11/04/2022	BA.2	BA.2	GRA	(NSP5_P132H,NSP3_G489S,Spike_L24del,NSP4_T327I,Spike_S373P,Spike_N969K,Spike_H655Y,N_R203K,Spike_V213G,Spike_G142D,Spike_A27S,Spike_Q954H,N_P13L,Spike_P25del,N_R32del,NS3_T223I,Spike_T19I,M_Q19E,Spike_N440K,NSP4_T492I,Spike_N679K,Spike_N764K,NSP6_G107del,N_G204R,M_A63T,Spike_S371F,Spike_K417N,NSP13_R392C,Spike_T376A,NSP6_S106del,Spike_G339D,Spike_R408S,NSP4_L438F,Spike_P681H,NSP4_L264F,NSP3_T24I,N_S33del,NSP1_S135R,Spike_S375F,Spike_D405N,N_E31del,NSP15_T112I,NSP6_F108del,E_T9I,Spike_P26del,NSP12_P323L,Spike_D614G)
EPI_ISL_12280342	11/04/2022	BA.2	BA.2.9	GR	(NSP5_P132H,NSP3_G489S,Spike_L24del,NSP4_T327I,Spike_S373P,Spike_N969K,Spike_H655Y,N_R203K,Spike_V213G,Spike_G142D,Spike_A27S,N_P13L,Spike_P25del,N_R32del,NS3_T223I,Spike_T19I,NSP3_A333V,M_Q19E,Spike_N440K,NSP4_T492I,Spike_N679K,Spike_N764K,NSP6_G107del,N_G204R,N_S413R,M_A63T,Spike_S371F,NS3_H78Y,Spike_K417N,NSP13_R392C,Spike_T376A,NSP6_S106del,Spike_G339D,Spike_R408S,NSP4_L438F,NSP14_I42V,NSP4_L264F,Spike_P681H,NSP3_T24I,N_S33del,NSP1_S135R,Spike_S375F,Spike_D405N,N_E31del,NSP15_T112I,NSP6_F108del,E_T9I,Spike_P26del,NSP12_P323L,Spike_D614G)
EPI_ISL_12280346	11/04/2022	BA.2	BA.2	GRA	(NSP5_P132H,NSP3_G489S,Spike_L24del,NSP4_T327I,Spike_S373P,Spike_N969K,Spike_H655Y,N_R203K,Spike_V213G,Spike_A27S,Spike_Q954H,N_P13L,Spike_P25del,N_R32del,NS3_T223I,Spike_T19I,M_Q19E,Spike_N440K,NSP4_T492I,Spike_N679K,Spike_N764K,NSP6_G107del,N_G204R,Spike_T478K,M_A63T,Spike_S371F,Spike_K417N,NSP13_R392C,Spike_T376A,NSP6_S106del,Spike_G339D,Spike_R408S,NSP4_L438F,NSP4_L264F,Spike_P681H,NSP3_T24I,N_S33del,NSP1_S135R,Spike_S375F,Spike_D405N,Spike_S477N,N_E31del,NSP15_T112I,NSP6_F108del,Spike_E484A,Spike_P26del,NSP12_P323L)
EPI_ISL_12280347	11/04/2022	BA.2	BA.2	GR	(NSP5_P132H,NSP3_G489S,Spike_L24del,NSP4_T327I,Spike_S373P,Spike_N969K,Spike_H655Y,N_R203K,Spike_V213G,Spike_G142D,Spike_A27S,Spike_Q954H,N_P13L,Spike_P25del,N_R32del,NS3_T223I,Spike_T19I,M_Q19E,Spike_N440K,NSP4_T492I,Spike_N679K,Spike_N764K,NSP14_A119V,NSP6_G107del,N_G204R,M_A63T,Spike_S371F,Spike_K417N,NSP13_R392C,Spike_T376A,NSP6_S106del,Spike_R408S,NSP4_L438F,Spike_P681H,NSP4_L264F,NSP3_T24I,N_S33del,NSP1_S135R,Spike_S375F,Spike_D405N,N_E31del,NSP8_P10S,NSP15_T112I,NSP6_F108del,E_T9I,Spike_P26del,NSP12_P323L,Spike_D614G)
EPI_ISL_12280356	11/04/2022	BA.2	BA.2.44	GR	(NSP5_P132H,NSP3_G489S,NSP13_R392C,Spike_K417N,Spike_L24del,NSP4_T327I,Spike_S373P,NSP6_S106del,Spike_T376A,Spike_N969K,Spike_H655Y,N_R203K,Spike_V213G,Spike_G142D,Spike_A27S,Spike_Q954H,N_P13L,Spike_R408S,Spike_P25del,N_R32del,NSP4_L438F,NS3_T223I,Spike_T19I,NSP14_I42V,Spike_P681H,NSP4_L264F,M_Q19E,NSP3_T24I,Spike_N440K,N_S33del,NSP1_S135R,NSP4_T492I,Spike_S375F,Spike_D405N,Spike_N679K,NSP6_G107del,N_E31del,NSP6_F108del,N_G204R,E_T9I,Spike_S371Y,Spike_P26del,M_A63T,NSP12_P323L,Spike_D614G)
EPI_ISL_12280358	11/04/2022	BA.3	BA.3.1	GRA	(NSP5_P132H,Spike_S371F,Spike_H69del,Spike_T95I,NSP6_A88V,Spike_A67V,NSP3_G489S,NSP4_T327I,Spike_S373P,NSP6_S106del,Spike_N969K,Spike_H655Y,N_R203K,Spike_G142D,Spike_K417T,Spike_Q954H,N_P13L,N_R32del,NS3_T223I,NSP14_I42V,Spike_P681H,Spike_Y144del,M_Q19E,N_S33del,NSP1_S135R,NSP4_T492I,Spike_S375F,Spike_D405N,NSP6_L33M,Spike_N679K,Spike_N764K,Spike_L212I,NSP6_G107del,N_E31del,Spike_N211del,NSP6_F108del,N_G204R,E_T9I,Spike_V70del,Spike_V143del,NSP3_A358N,M_A63T,NSP12_P323L,Spike_Y145del,Spike_D614G)
EPI_ISL_12280359	11/04/2022	BA.2	BA.2	GR	(NSP5_P132H,NSP3_G489S,Spike_L24del,NS3_S40L,NSP4_T327I,Spike_S373P,Spike_N969K,Spike_H655Y,N_R203K,Spike_V213G,Spike_G142D,Spike_A27S,Spike_Q954H,N_P13L,Spike_P25del,N_R32del,NS3_T223I,Spike_T19I,M_Q19E,Spike_N440K,NSP4_T492I,Spike_N679K,Spike_N764K,NSP6_G107del,N_G204R,M_A63T,Spike_S371F,Spike_K417N,NSP13_R392C,NSP3_T1334I,Spike_T376A,NSP6_S106del,Spike_R408S,NSP4_L438F,NSP14_I42V,Spike_P681H,NSP4_L264F,NSP3_T24I,N_S33del,NSP1_S135R,Spike_S375F,Spike_D405N,N_E31del,NSP6_F108del,E_T9I,Spike_P26del,NSP12_P323L,Spike_D614G)

EPI_ISL_12280360	11/04/2022	BA.2	BA.2	GR	(NSP5_P132H,NSP3_G489S,Spike_L24del,NSP4_T327I,Spike_S373P,Spike_N969K,Spike_H655Y,N_G243C,N_R203K,Spike_V213G,Spike_G142D,Spike_A27S,Spike_Q954H,N_P13L,Spike_P25del,N_R32del,NS3_T223I,Spike_T19I,M_Q19E,Spike_N440K,NSP4_T492I,Spike_N679K,Spike_N764K,NSP6_G107del,N_G204R,M_A63T,Spike_S371F,Spike_K417N,NSP13_R392C,Spike_T376A,NSP6_S106del,Spike_R408S,NSP4_L438F,Spike_P681H,NSP4_L264F,NSP3_T24I,N_S33del,NSP1_S135R,Spike_S375F,Spike_D405N,N_E31del,NSP15_T112I,NSP6_F108del,E_T9I,Spike_P26del,NSP12_P323L,Spike_D614G)
EPI_ISL_12288955	11/04/2022	BA.2	BA.2.3.15	GR	(NSP5_P132H,NSP3_G489S,Spike_L24del,NSP4_T327I,Spike_S373P,Spike_N969K,Spike_H655Y,N_R203K,Spike_V213G,Spike_G142D,Spike_A27S,Spike_Q954H,N_P13L,Spike_P25del,N_R32del,NS3_T223I,Spike_T19I,M_Q19E,Spike_N440K,NSP4_T492I,Spike_N679K,Spike_N764K,NSP6_G107del,N_G204R,M_A63T,Spike_S371F,Spike_K417N,NSP13_R392C,Spike_T376A,NSP6_S106del,Spike_R408S,NSP4_L438F,NSP4_A146V,Spike_P681H,NSP4_L264F,NSP3_T24I,N_S33del,NSP1_S135R,Spike_S375F,Spike_D405N,Spike_D215E,N_E31del,NSP15_T112I,NSP6_F108del,E_T9I,Spike_P26del,NSP12_P323L,Spike_D614G)
EPI_ISL_12288956	11/04/2022	BA.2	BA.2	GRA	(NSP5_P132H,NSP3_G489S,Spike_L24del,NSP4_T327I,Spike_S373P,Spike_N969K,Spike_H655Y,N_R203K,Spike_V213G,Spike_G142D,Spike_A27S,Spike_Q954H,N_P13L,Spike_P25del,N_R32del,NS3_T223I,NSP2_S263P,Spike_T19I,M_Q19E,Spike_N440K,NSP4_T492I,Spike_N679K,Spike_N764K,NSP6_G107del,NS8_S67F,N_G204R,M_A63T,Spike_S371F,Spike_K417N,NSP13_R392C,NSP1_A131V,Spike_T376A,NSP6_S106del,Spike_G339D,Spike_R408S,NSP4_L438F,NSP4_L264F,Spike_P681H,NSP3_T24I,N_S33del,NSP1_S135R,Spike_S375F,Spike_D405N,N_E31del,NSP15_T112I,NSP6_F108del,E_T9I,Spike_P26del,NSP12_P323L,Spike_D614G)
EPI_ISL_12323369	11/04/2022	BA.2	BA.2	GRA	(NSP5_P132H,NSP3_G489S,Spike_L24del,NSP4_T327I,Spike_S373P,Spike_Q493R,Spike_N969K,Spike_H655Y,N_R203K,Spike_V213G,Spike_G142D,Spike_A27S,Spike_Q954H,N_P13L,Spike_P25del,N_R32del,NS3_T223I,Spike_T19I,M_Q19E,Spike_N440K,NSP4_T492I,Spike_N679K,Spike_N764K,NSP6_G107del,N_G204R,Spike_T478K,N_S413R,M_A63T,Spike_S371F,Spike_K417N,NSP13_R392C,Spike_T376A,NSP6_S106del,Spike_G339D,Spike_R408S,NSP4_L438F,Spike_P681H,NSP4_L264F,NSP3_T24I,N_S33del,NSP1_S135R,Spike_S375F,Spike_D405N,Spike_S477N,N_E31del,NSP15_T112I,NSP6_F108del,Spike_E484A,Spike_P26del,NSP12_P323L,Spike_D614G)
EPI_ISL_12323370	11/04/2022	BA.2	BA.2	GR	(NSP5_P132H,NSP3_G489S,Spike_L24del,NSP4_T327I,Spike_S373P,Spike_H655Y,N_R203K,Spike_V213G,Spike_G142D,Spike_A27S,Spike_Q954H,N_P13L,NSP3_L1266I,Spike_P25del,N_R32del,NS3_T223I,Spike_T19I,M_Q19E,NSP4_T492I,Spike_N679K,Spike_N764K,NSP6_G107del,Spike_D796Y,N_G204R,N_S413R,M_A63T,Spike_S371F,Spike_K417N,NSP16_V9I,NSP13_R392C,Spike_P209S,Spike_T376A,NSP6_S106del,NSP16_S243L,Spike_R408S,NSP4_L438F,NSP3_S1265del,NSP4_L264F,Spike_P681H,NSP16_Y242D,N_S33del,NSP1_S135R,Spike_S375F,Spike_D405N,N_E31del,NSP8_P10S,NSP6_F108del,E_T9I,Spike_P26del,NSP12_P323L,Spike_D614G)
EPI_ISL_12280317	12/04/2022	BA.2	BA.2	GRA	(NSP3_Q1821R,NSP5_P132H,Spike_S371F,N_P6L,NSP3_S1817A,NSP3_G489S,NSP13_R392C,Spike_K417N,Spike_L24del,NSP4_T327I,Spike_S373P,NSP6_S106del,Spike_T376A,Spike_N969K,Spike_H655Y,N_R203K,Spike_V213G,Spike_A27S,Spike_Q954H,Spike_G339D,N_P13L,Spike_R408S,Spike_P25del,N_R32del,NSP4_L438F,NS3_T223I,Spike_T19I,NSP4_L264F,Spike_P681H,M_Q19E,Spike_N440K,N_S33del,NSP1_S135R,NSP4_T492I,Spike_S375F,Spike_D405N,Spike_N679K,Spike_N764K,NSP6_G107del,N_E31del,NSP15_T112I,NSP6_F108del,N_G204R,Spike_P26del,M_A63T,NSP12_P323L)
EPI_ISL_12280318	12/04/2022	BA.2	BA.2	GRA	(NSP5_P132H,NSP3_G489S,Spike_L24del,Spike_K964R,Spike_S373P,N_N27S,Spike_N969K,Spike_H655Y,N_R203K,Spike_V213G,Spike_A27S,Spike_Q954H,N_P13L,Spike_P25del,N_R32del,NS3_T223I,Spike_T19I,M_Q19E,Spike_N440K,NSP4_T492I,Spike_N679K,Spike_N764K,NSP6_G107del,N_G204R,Spike_T478K,N_S413R,M_A63T,Spike_S371F,Spike_K417N,NSP13_R392C,Spike_T376A,NSP6_S106del,Spike_G339D,Spike_R408S,NSP4_L264F,Spike_P681H,N_S33del,NSP1_S135R,Spike_S375F,Spike_D405N,Spike_S477N,N_E31del,NSP15_T112I,NSP6_F108del,Spike_E484A,Spike_P26del,NSP12_P323L)
EPI_ISL_12280320	12/04/2022	BA.1	BA.1.17	GRA	(NSP5_P132H,Spike_H69del,Spike_T95I,Spike_A67V,Spike_K964R,Spike_S373P,Spike_N969K,Spike_H655Y,Spike_N856K,N_R203K,Spike_G142D,Spike_Q954H,N_P13L,NSP3_L1266I,N_R32del,Spike_S939F,M_Q19E,Spike_N440K,NSP4_T492I,NSP6_L105del,Spike_N679K,Spike_N764K,Spike_L212I,NSP6_G107del,Spike_T547K,M_D3G,N_G204R,Spike_V143del,M_A63T,NSP3_P985S,NSP3_V1069I,Spike_K417N,NSP6_S106del,Spike_S371L,Spike_G339D,NSP3_S1265del,Spike_P681H,Spike_Y144del,Spike_ins214EPE,N_S33del,Spike_S375F,Spike_G446S,N_E31del,Spike_N211del,Spike_V70del,Spike_L981F,NSP12_P323L,Spike_Y145del)
EPI_ISL_12280323	12/04/2022	BA.2	BA.2.3.15	GR	(NSP5_P132H,NSP3_G489S,Spike_L24del,NSP4_T327I,Spike_S373P,Spike_N969K,Spike_H655Y,N_R203K,Spike_V213G,Spike_G142D,Spike_A27S,Spike_Q954H,N_P13L,Spike_P25del,N_R32del,NS3_T223I,Spike_T19I,M_Q19E,Spike_N440K,NSP4_T492I,Spike_N679K,Spike_N764K,NSP6_G107del,N_G204R,NSP3_E115D,M_A63T,Spike_S371F,Spike_K417N,NSP13_R392C,Spike_T376A,NSP6_S106del,Spike_R408S,NSP4_L438F,NSP4_A146V,NSP4_L264F,Spike_P681H,NSP3_T24I,N_S33del,NSP1_S135R,Spike_S375F,Spike_D405N,NSP2_R27C,Spike_D215E,N_E31del,NSP6_F108del,NSP13_C441S,E_T9I,Spike_P26del,NSP12_P323L,Spike_D614G)
EPI_ISL_12280324	12/04/2022	BA.2	BA.2.10	GRA	(NSP5_P132H,NSP12_G671S,NSP3_G489S,Spike_L24del,NSP4_T327I,Spike_S373P,Spike_N969K,Spike_H655Y,N_R203K,Spike_V213G,Spike_G142D,Spike_A27S,Spike_Q954H,N_P13L,Spike_P25del,N_R32del,NS3_T223I,Spike_T19I,M_Q19E,Spike_N440K,NSP4_T492I,Spike_N679K,Spike_N764K,NSP6_G107del,N_G204R,M_A63T,Spike_S371F,Spike_K417N,NSP13_R392C,Spike_T376A,NSP6_S106del,Spike_G339D,Spike_R408S,NSP4_L438F,NSP14_I42V,NSP4_L264F,Spike_P681H,NSP3_T24I,N_S33del,NSP1_S135R,Spike_S375F,NSP13_S36P,N_E31del,NSP15_T112I,NSP6_F108del,E_T9I,Spike_P26del,NSP12_P323L,Spike_D614G)
EPI_ISL_12280325	12/04/2022	BA.2	BA.2	O	(NSP5_P132H,Spike_S371F,NSP3_G489S,NSP13_R392C,Spike_K417N,Spike_L24del,NSP4_T327I,Spike_S373P,NSP6_S106del,Spike_T376A,Spike_H655Y,N_R203K,Spike_V213G,Spike_A27S,Spike_Q954H,Spike_G339D,N_P13L,Spike_R408S,Spike_P25del,N_R32del,NSP4_L438F,NS3_T223I,Spike_T19I,NSP4_L264F,Spike_P681H,M_Q19E,Spike_N440K,N_S33del,NSP1_S135R,NSP4_T492I,Spike_S375F,Spike_D405N,Spike_N679K,Spike_N764K,NSP6_G107del,N_E31del,NSP15_T112I,NSP6_F108del,N_G204R,N_S413R,Spike_P26del,M_A63T,NSP12_P323L)
EPI_ISL_12280327	12/04/2022	BA.2	BA.2	GR	(NSP5_P132H,NSP3_G489S,Spike_L24del,NSP4_T327I,Spike_S373P,Spike_N969K,Spike_H655Y,N_R203K,Spike_V213G,Spike_G142D,Spike_A27S,NSP13_T351I,Spike_Q954H,N_P13L,Spike_P25del,N_R32del,NS3_T223I,Spike_T19I,M_Q19E,Spike_N440K,NSP4_T492I,Spike_N679K,Spike_N764K,NSP6_G107del,N_G204R,M_A63T,NSP4_T461I,Spike_S371F,Spike_K417N,NSP13_R392C,Spike_T376A,NSP6_S106del,Spike_R408S,NSP14_I42V,Spike_P681H,NSP4_L264F,NSP3_T24I,N_S33del,NSP1_S135R,Spike_S375F,Spike_D405N,N_E31del,NSP15_T112I,NSP6_F108del,E_T9I,Spike_P26del,NSP12_P323L,Spike_D614G)
EPI_ISL_12280329	12/04/2022	BA.2	BA.2	O	(NSP5_P132H,Spike_S371F,NSP3_G489S,NSP13_R392C,Spike_K417N,Spike_L24del,NSP4_T327I,Spike_S373P,NSP6_S106del,Spike_T376A,Spike_N969K,Spike_H655Y,N_R203K,Spike_V213G,Spike_A27S,Spike_Q954H,N_P13L,Spike_R408S,Spike_P25del,N_R32del,NSP4_L438F,NS3_T223I,Spike_T19I,NSP4_L264F,Spike_P681H,M_Q19E,NSP3_T24I,Spike_N440K,N_S33del,NSP1_S135R,NSP4_T492I,Spike_S375F,NS6_D53S,Spike_D405N,Spike_N679K,Spike_N764K,NSP6_G107del,N_E31del,NSP6_F108del,N_G204R,NS6_L52R,Spike_P26del,M_A63T,NSP12_P323L)

EPI_ISL_12280330	12/04/2022	BA.1	BA.1.1.1	GRA	(NSP5_P132H,Spike_H69del,Spike_T95I,Spike_A67V,Spike_S373P,Spike_N969K,Spike_H655Y,Spike_N856K,N_R203K,Spike_Y449F,Spike_G142D,Spike_Q954H,N_P13L,NSP3_L1266I,N_R32del,M_Q19E,NSP4_T492I,NSP6_L105del,Spike_N679K,Spike_N764K,Spike_L212I,NSP6_G107del,Spike_R346K,NSP6_I189V,Spike_T547K,M_D3G,N_G204R,NS6_L52R,Spike_S758G,Spike_V143del,M_A63T,NSP12_Q875R,Spike_N777K,Spike_K417N,NSP6_S106del,Spike_S371L,Spike_G339D,NSP3_S1265del,NSP14_I42V,Spike_P681H,Spike_Y144del,Spike_ins214EPE,N_S33del,Spike_S375F,NS6_D53S,N_E31del,NSP3_K38R,Spike_N211del,Spike_C760F,E_T9I,Spike_V70del,Spike_L981F,NSP12_P323L,Spike_Y145del,NSP3_P200S)
EPI_ISL_12280357	12/04/2022	BA.1	BA.1	GRA	(NSP5_P132H,Spike_H69del,Spike_T95I,Spike_A67V,Spike_H655Y,Spike_N856K,N_R203K,Spike_G142D,NSP3_A1892T,Spike_Q954H,N_P13L,NSP3_L1266I,N_R32del,M_Q19E,Spike_N440K,NSP4_T492I,NSP6_L105del,Spike_N679K,Spike_N764K,Spike_L212I,NSP6_G107del,Spike_R346K,NSP6_I189V,Spike_T547K,M_D3G,N_G204R,NS6_L52R,Spike_V143del,M_A63T,Spike_K417N,NSP6_S106del,Spike_G339D,NSP3_S1265del,NSP14_I42V,Spike_P681H,Spike_Y144del,Spike_ins214EPE,N_S33del,NS6_D53S,Spike_G446S,N_E31del,NSP3_K38R,Spike_N211del,E_T9I,Spike_V70del,NSP12_P323L,Spike_Y145del,Spike_D614G,NSP12_S27N)
EPI_ISL_12323355	12/04/2022	BA.2	BA.2	GRA	(NSP5_P132H,NSP3_G489S,Spike_L24del,NSP4_T327I,Spike_S373P,Spike_Q493R,Spike_N969K,Spike_H655Y,N_R203K,Spike_V213G,Spike_G142D,Spike_A27S,Spike_Q954H,N_P13L,Spike_P25del,N_R32del,NS3_T223I,Spike_T19I,M_Q19E,Spike_N440K,NSP4_T492I,Spike_N679K,Spike_N764K,NSP6_G107del,N_G204R,Spike_T478K,N_S413R,M_A63T,Spike_S371F,Spike_K417N,NSP13_R392C,Spike_T376A,NSP6_S106del,NSP2_L444F,Spike_R408S,NSP4_L438F,Spike_P681H,NSP4_L264F,NSP3_T24I,N_S33del,NSP1_S135R,Spike_S375F,Spike_D405N,Spike_S477N,N_E31del,NSP15_T112I,NSP6_F108del,Spike_E484A,Spike_P26del,NSP12_P323L,Spike_D614G)
EPI_ISL_12323356	12/04/2022	BA.2	BA.2.3.15	GRA	(NSP5_P132H,NSP3_G489S,Spike_L24del,NSP4_T327I,Spike_S373P,Spike_Q493R,Spike_N969K,Spike_H655Y,N_R203K,Spike_V213G,Spike_G142D,Spike_A27S,Spike_Q954H,N_P13L,Spike_P25del,N_R32del,NS3_T223I,Spike_T19I,M_Q19E,Spike_N440K,NSP4_T492I,Spike_N679K,Spike_N764K,NSP6_G107del,N_G204R,Spike_T478K,N_S413R,M_A63T,NSP6_M143I,Spike_S371F,Spike_K417N,NSP13_R392C,Spike_T376A,NSP6_S106del,Spike_G339D,Spike_R408S,NSP4_L438F,NSP4_A146V,NSP4_L264F,Spike_P681H,NSP3_T24I,N_S33del,NSP1_S135R,Spike_S375F,Spike_D405N,Spike_D215E,Spike_S477N,N_E31del,NSP15_T112I,NSP6_F108del,Spike_E484A,Spike_P26del,NSP12_P323L,Spike_D614G)
EPI_ISL_12323357	12/04/2022	BA.2	BA.2	GRA	(NSP5_P132H,NSP3_G489S,Spike_L24del,NSP4_T327I,Spike_S373P,Spike_Q493R,Spike_N969K,Spike_H655Y,N_R203K,Spike_V213G,Spike_G142D,Spike_A27S,Spike_Q954H,N_P13L,Spike_P25del,N_R32del,NS3_T223I,Spike_T19I,M_Q19E,Spike_N440K,NSP4_T492I,Spike_N679K,Spike_N764K,NSP6_G107del,N_G204R,Spike_T478K,N_S413R,M_A63T,Spike_S371F,Spike_K417N,NSP13_R392C,Spike_T376A,NSP6_S106del,Spike_G339D,Spike_R408S,NSP4_L438F,Spike_P681H,NSP4_L264F,N_S33del,NSP1_S135R,Spike_S375F,Spike_D405N,Spike_S477N,N_E31del,NSP15_T112I,NSP6_F108del,Spike_E484A,Spike_P26del,NSP12_P323L,Spike_D614G)
EPI_ISL_12323362	12/04/2022	BA.2	BA.2	GRA	(NSP5_P132H,Spike_L24del,NSP4_T327I,Spike_S373P,Spike_Q493R,Spike_N969K,Spike_H655Y,N_R203K,Spike_V213G,Spike_G142D,Spike_A27S,Spike_Q954H,N_P13L,Spike_P25del,N_R32del,NS3_T223I,Spike_T19I,M_Q19E,Spike_N440K,NSP4_T492I,Spike_N679K,Spike_N764K,NSP6_G107del,N_G204R,Spike_T478K,N_S413R,M_A63T,Spike_S371F,NSP16_M17I,Spike_K417N,NSP13_R392C,Spike_T376A,NSP6_S106del,Spike_R408S,NSP4_L438F,Spike_P681H,NSP4_L264F,N_S33del,Spike_S375F,Spike_D405N,Spike_S477N,N_E31del,NSP15_T112I,NSP6_F108del,Spike_E484A,E_T9I,Spike_P26del,NSP12_P323L,Spike_D614G)
EPI_ISL_12323365	12/04/2022	BA.2	BA.2	GRA	(NSP5_P132H,NSP3_G489S,Spike_L24del,NSP4_T327I,Spike_S373P,Spike_Q493R,Spike_N969K,Spike_H655Y,N_R203K,Spike_V213G,Spike_G142D,Spike_A27S,Spike_Q954H,N_P13L,Spike_P25del,N_R32del,NS3_T223I,Spike_T19I,NSP3_T350I,M_Q19E,Spike_N440K,NSP4_T492I,Spike_N679K,Spike_N764K,NSP6_G107del,N_G204R,Spike_T478K,N_S413R,M_A63T,Spike_S371F,Spike_K417N,NSP13_R392C,Spike_T376A,NSP6_S106del,Spike_R408S,NSP4_L438F,Spike_P681H,NSP4_L264F,NSP3_T24I,N_S33del,NSP1_S135R,Spike_S375F,Spike_D405N,Spike_S477N,N_E31del,NSP15_T112I,NSP6_F108del,Spike_E484A,Spike_P26del,NSP12_P323L,Spike_D614G)
EPI_ISL_12323366	12/04/2022	BA.2	BA.2.10	GRA	(NSP5_P132H,NSP3_G489S,Spike_L24del,NSP4_T327I,Spike_S373P,Spike_Q493R,Spike_N969K,Spike_H655Y,N_R203K,Spike_V213G,Spike_G142D,Spike_A27S,Spike_Q954H,N_P13L,Spike_P25del,N_R32del,NS3_T223I,Spike_T19I,M_Q19E,Spike_N440K,NSP4_T492I,Spike_N679K,Spike_N764K,NSP6_G107del,N_G204R,Spike_T478K,NSP12_L638F,N_S413R,M_A63T,Spike_S371F,Spike_K417N,NSP13_R392C,Spike_T376A,NSP6_S106del,Spike_R408S,NSP4_L438F,NSP4_L264F,Spike_P681H,NSP3_T24I,N_S33del,NSP1_S135R,Spike_S375F,Spike_D405N,NSP13_S36P,Spike_S477N,N_E31del,NSP15_T112I,NSP6_F108del,Spike_E484A,Spike_P26del,NSP12_P323L,Spike_D614G)
EPI_ISL_12323367	12/04/2022	BA.2	BA.2	GRA	(NSP5_P132H,NSP3_G489S,Spike_L24del,NSP4_T327I,Spike_S373P,Spike_Q493R,Spike_N969K,Spike_H655Y,N_R203K,Spike_V213G,Spike_G142D,NSP12_V424A,Spike_A27S,Spike_Q954H,N_P13L,Spike_P25del,N_R32del,Spike_T19I,M_Q19E,Spike_N440K,NSP4_T492I,Spike_N679K,Spike_N764K,NSP6_G107del,NS3_T223S,N_G204R,Spike_T478K,N_S413R,M_A63T,Spike_S371F,Spike_K417N,NSP13_R392C,Spike_T376A,NSP6_S106del,Spike_R408S,NSP4_L438F,Spike_P681H,NSP4_L264F,NSP3_T24I,N_S33del,NSP1_S135R,Spike_S375F,Spike_D405N,Spike_S477N,N_E31del,NSP15_T112I,NSP6_F108del,Spike_E484A,Spike_P26del,NSP12_P323L,Spike_D614G)
EPI_ISL_12280316	13/04/2022	BA.2	BA.2	GRA	(NSP5_P132H,Spike_S371F,NSP3_G489S,NSP13_R392C,Spike_K417N,Spike_L24del,NSP4_T327I,Spike_S373P,NSP6_S106del,Spike_T376A,Spike_N969K,Spike_H655Y,N_R203K,Spike_V213G,Spike_A27S,Spike_Q954H,Spike_G339D,N_P13L,Spike_R408S,Spike_P25del,N_R32del,NSP4_L438F,NS3_T223I,Spike_T19I,NSP4_L264F,Spike_P681H,N_S416L,M_Q19E,Spike_N440K,N_S33del,NSP1_S135R,NSP4_T492I,Spike_S375F,Spike_D405N,Spike_N679K,Spike_N764K,NSP6_G107del,N_E31del,NSP15_T112I,NSP6_F108del,N_G204R,N_S413R,Spike_P26del,M_A63T,NSP12_P323L)
EPI_ISL_12280321	13/04/2022	BA.2	BA.2.10	GRA	(NSP5_P132H,NSP3_G489S,Spike_L24del,NSP4_T327I,Spike_S373P,Spike_N969K,Spike_H655Y,N_R203K,Spike_V213G,Spike_G142D,Spike_A27S,Spike_Q954H,N_P13L,Spike_P25del,N_R32del,NS3_T223I,Spike_T19I,M_Q19E,Spike_N440K,NSP4_T492I,Spike_N679K,Spike_N764K,NSP6_G107del,N_G204R,Spike_T478K,NS6_L52R,M_A63T,Spike_S371F,Spike_K417N,NSP13_R392C,Spike_T376A,NSP6_S106del,Spike_G339D,Spike_R408S,NSP4_L438F,NSP4_L264F,Spike_P681H,NSP3_T24I,N_S33del,NSP1_S135R,Spike_S375F,NS6_D53S,Spike_D405N,NSP13_S36P,Spike_S477N,N_E31del,NSP15_T112I,NSP6_F108del,Spike_E484A,Spike_P26del,NSP12_P323L)
EPI_ISL_12280328	13/04/2022	BA.2	BA.2.9	GRA	(NSP5_P132H,NSP3_G489S,Spike_L24del,NSP4_T327I,Spike_S373P,Spike_N969K,Spike_H655Y,N_R203K,Spike_V213G,Spike_G142D,Spike_A27S,Spike_Q954H,N_P13L,Spike_P25del,N_R32del,NS3_T223I,Spike_T19I,M_Q19E,Spike_N440K,NSP4_T492I,Spike_N679K,Spike_N764K,NSP6_G107del,N_G204R,M_A63T,Spike_S371F,NS3_H78Y,Spike_K417N,NSP13_R392C,Spike_T376A,NSP6_S106del,Spike_G339D,Spike_R408S,NSP4_L438F,Spike_P681H,NSP4_L264F,NSP3_T24I,N_S33del,NSP1_S135R,Spike_S375F,Spike_D405N,N_E31del,NSP15_T112I,NSP6_F108del,E_T9I,Spike_P26del,NSP12_P323L,Spike_D614G)
EPI_ISL_12280344	13/04/2022	BA.2	BA.2	GRA	(NSP5_P132H,NSP3_G489S,Spike_L24del,NSP4_T327I,Spike_S373P,Spike_N969K,Spike_H655Y,N_R203K,Spike_V213G,Spike_G142D,Spike_A27S,Spike_Q954H,N_P13L,Spike_P25del,N_R32del,NS3_T223I,Spike_T19I,M_Q19E,Spike_N440K,NSP4_T492I,Spike_N679K,Spike_N764K,NSP6_G107del,N_G204R,N_S413R,M_A63T,Spike_S371F,Spike_K417N,NSP13_R392C,Spike_T376A,NSP6_S106del,Spike_G339D,Spike_R408S,NSP4_L438F,Spike_P681H,NSP4_L264F,NSP3_T24I,N_S33del,NSP1_S135R,Spike_S375F,Spike_D405N,N_E31del,NSP15_T112I,NSP6_F108del,E_T9I,Spike_P26del,NSP12_P323L,Spike_D614G)

EPI_ISL_12280351	13/04/2022	BA.2	BA.2	GRA	(NSP5_P132H,Spike_S371F,NSP3_G489S,NSP13_R392C,Spike_K417N,Spike_L24del,NSP4_T327I,Spike_S373P,NSP6_S106del,Spike_T376A,Spike_N969K,Spike_H655Y,N_R203K,Spike_V213G,Spike_A27S,Spike_Q954H,Spike_G339D,N_P13L,Spike_R408S,Spike_P25del,N_R32del,NSP4_L438F,NS3_T223I,Spike_T19I,NSP4_L264F,Spike_P681H,M_Q19E,Spike_N440K,N_S33del,NSP1_S135R,NSP4_T492I,Spike_S375F,Spike_D405N,Spike_N679K,Spike_N764K,NSP6_G107del,N_E31del,NSP15_T112I,NSP6_F108del,N_G204R,Spike_P26del,M_A63T,NSP12_P323L)
EPI_ISL_12280352	13/04/2022	BA.2	BA.2.3	GRA	(NSP5_P132H,NSP3_G489S,Spike_L24del,NSP4_T327I,Spike_S373P,Spike_N969K,Spike_H655Y,N_R203K,Spike_V213G,Spike_A27S,Spike_Q954H,N_P13L,Spike_P25del,N_R32del,NS3_T223I,Spike_T19I,M_Q19E,Spike_N440K,NSP4_T492I,Spike_N679K,Spike_N764K,NSP6_G107del,N_G204R,Spike_T478K,M_A63T,Spike_S371F,Spike_K417N,NSP13_R392C,Spike_T376A,NSP6_S106del,Spike_G339D,Spike_R408S,NSP4_L438F,NSP4_A146V,NSP4_L264F,Spike_P681H,NSP3_T24I,N_S33del,NSP1_S135R,Spike_S375F,Spike_D405N,Spike_S477N,N_E31del,NSP15_T112I,NSP6_F108del,Spike_E484A,Spike_P26del,NSP12_P323L)
EPI_ISL_12280353	13/04/2022	BA.2	BA.2.9	GR	(NSP5_P132H,NSP3_G489S,Spike_L24del,NSP4_T327I,Spike_S373P,Spike_N969K,Spike_H655Y,N_R203K,Spike_V213G,Spike_G142D,Spike_A27S,Spike_Q954H,N_P13L,NSP1_H83del,Spike_P25del,N_R32del,NS3_T223I,Spike_T19I,M_Q19E,Spike_N440K,NSP4_T492I,Spike_N679K,Spike_N764K,NSP6_G107del,NSP1_V84del,N_G204R,NS6_L52R,M_A63T,Spike_S371F,NSP1_M85del,NS3_H78Y,Spike_K417N,NSP13_R392C,Spike_T376A,NSP6_S106del,Spike_R408S,NSP4_L438F,NSP4_L264F,Spike_P681H,NSP3_T24I,NSP1_G82del,N_S33del,NSP1_S135R,Spike_S375F,NS6_D53S,Spike_D405N,N_E31del,NSP1_V86del,NSP3_A1461V,NSP6_F108del,E_T9I,Spike_P26del,NSP12_P323L,Spike_D614G)
EPI_ISL_12280354	13/04/2022	BA.2	BA.2	GR	(NSP5_P132H,NSP3_G489S,Spike_L24del,NSP4_T327I,Spike_S373P,Spike_N969K,Spike_H655Y,N_R203K,Spike_V213G,Spike_S1252P,Spike_G142D,Spike_A27S,Spike_Q954H,N_P13L,Spike_P25del,N_R32del,NS3_T223I,Spike_T19I,M_Q19E,Spike_N440K,NSP4_T492I,Spike_N679K,Spike_N764K,NSP6_G107del,N_G204R,M_A63T,NSP2_N195S,Spike_S371F,Spike_K417N,NSP13_R392C,Spike_T376A,NSP6_S106del,Spike_R408S,NSP4_L438F,NSP14_I42V,Spike_P681H,NSP4_L264F,NSP3_T24I,N_S33del,NSP1_S135R,Spike_S375F,Spike_D405N,N_E31del,NSP6_F108del,E_T9I,Spike_P26del,NSP12_P323L,Spike_D614G)
EPI_ISL_12280355	13/04/2022	BA.2	BA.2	GR	(NSP5_P132H,NSP3_G489S,Spike_L24del,NSP4_T327I,Spike_S373P,Spike_N969K,Spike_H655Y,N_R203K,Spike_V213G,Spike_G142D,Spike_A27S,Spike_Q954H,N_P13L,Spike_P25del,N_R32del,NS3_T223I,Spike_T19I,M_Q19E,Spike_N440K,NSP4_T492I,Spike_N679K,Spike_N764K,NSP6_G107del,NSP12_T739I,N_G204R,M_A63T,NSP2_T149A,Spike_S371F,Spike_K417N,NSP13_R392C,Spike_T376A,NSP6_S106del,Spike_R408S,NSP4_L438F,NSP14_I42V,NSP4_L264F,Spike_P681H,NSP3_T24I,N_S33del,NSP1_S135R,Spike_S375F,Spike_D405N,N_E31del,NSP15_T112I,NSP6_F108del,E_T9I,Spike_P26del,NSP12_P323L,Spike_D614G,NSP12_S27R)
EPI_ISL_12323358	13/04/2022	BA.2	BA.2	GR	(NSP5_P132H,Spike_S371F,NSP3_G489S,NSP13_R392C,Spike_K417N,Spike_L24del,NSP4_T327I,Spike_S373P,NSP6_S106del,Spike_T376A,Spike_H655Y,N_R203K,Spike_V213G,Spike_G142D,Spike_A27S,Spike_Q954H,N_P13L,Spike_R408S,Spike_P25del,N_R32del,NSP4_L438F,NS3_T223I,Spike_T19I,Spike_P681H,NSP4_L264F,M_Q19E,NSP3_T24I,Spike_N440K,N_S33del,NSP1_S135R,NSP4_T492I,Spike_S375F,Spike_D405N,Spike_N679K,Spike_N764K,NSP6_G107del,N_E31del,NSP6_F108del,N_G204R,E_T9I,N_S413R,Spike_P26del,M_A63T,NSP12_P323L,Spike_D614G)
EPI_ISL_12323359	13/04/2022	BA.2	BA.2	GR	(NSP5_P132H,NSP12_I466L,NSP13_R392C,Spike_K417N,Spike_L24del,Spike_P209S,NSP6_S106del,Spike_N969K,Spike_H655Y,N_R203K,Spike_Y449F,Spike_V213G,Spike_A27S,NSP16_S243L,Spike_Q954H,NS8_F120L,N_P13L,Spike_R408S,Spike_P25del,N_R32del,NSP4_L438F,NS3_T223I,Spike_T19I,NS6_K42N,NSP4_L264F,NSP16_Y242D,M_Q19E,N_S33del,NSP4_T492I,NS6_D53S,Spike_D405N,Spike_N764K,NSP6_G107del,N_E31del,NSP6_F108del,N_G204R,N_S413R,NS6_L52R,Spike_P26del,M_A63T,NSP12_P323L,Spike_V1128L,Spike_D614G)
EPI_ISL_12323360	13/04/2022	BA.2	BA.2	GRA	(NSP5_P132H,NSP3_G489S,Spike_L24del,NSP4_T327I,Spike_S373P,Spike_N969K,Spike_H655Y,N_R203K,Spike_V213G,Spike_G142D,Spike_A27S,Spike_Q954H,N_P13L,Spike_P25del,N_R32del,NS3_T223I,Spike_T19I,M_Q19E,Spike_N440K,NSP4_T492I,Spike_N679K,Spike_N764K,NSP6_G107del,N_G204R,N_S413R,M_A63T,Spike_S371F,Spike_K417N,NSP13_R392C,Spike_P209S,Spike_T376A,NSP6_S106del,Spike_G339D,NS8_F120L,Spike_R408S,NSP4_L438F,Spike_P681H,NSP4_L264F,NSP3_T24I,N_S33del,NSP1_S135R,Spike_S375F,Spike_D405N,N_E31del,NSP8_P10S,NSP6_F108del,E_T9I,Spike_P26del,NSP12_P323L,Spike_D614G)
EPI_ISL_12323372	13/04/2022	BA.2	BA.2.3.15	GRA	(NSP5_P132H,NSP3_G489S,Spike_L24del,NSP4_T327I,Spike_S373P,Spike_N969K,Spike_H655Y,N_R203K,Spike_V213G,Spike_G142D,Spike_A27S,Spike_Q954H,N_P13L,Spike_P25del,N_R32del,NS3_T223I,Spike_T19I,M_Q19E,Spike_N440K,NSP4_T492I,Spike_N679K,Spike_N764K,NSP6_G107del,N_G204R,Spike_T478K,N_S413R,M_A63T,Spike_S371F,Spike_K417N,NSP13_R392C,Spike_T376A,NSP6_S106del,Spike_R408S,NSP4_L438F,NSP4_A146V,Spike_P681H,NSP4_L264F,N_S33del,NSP1_S135R,Spike_S375F,Spike_D405N,Spike_D215E,Spike_S477N,N_E31del,NSP15_T112I,NSP6_F108del,Spike_E484A,Spike_P26del,NSP12_P323L,Spike_D614G)
EPI_ISL_12280322	14/04/2022	BA.1	BA.1	GRA	(NSP5_P132H,Spike_H69del,Spike_T95I,Spike_A67V,Spike_S373P,Spike_N969K,Spike_H655Y,Spike_N856K,N_R203K,Spike_G142D,Spike_Q954H,N_P13L,NSP3_L1266I,N_R32del,M_Q19E,Spike_N440K,NSP4_T492I,NSP6_L105del,Spike_N679K,Spike_N764K,Spike_L212I,NSP6_G107del,Spike_R346K,NSP3_T656I,Spike_T547K,M_D3G,N_G204R,NS6_L52R,Spike_V143del,M_A63T,Spike_K417N,NSP6_S106del,Spike_S371L,Spike_G339D,NSP3_S1265del,Spike_P681H,Spike_Y144del,Spike_ins214EPE,N_S33del,Spike_S375F,NS6_D53S,Spike_G446S,N_E31del,Spike_N211del,Spike_V70del,Spike_L981F,NSP12_P323L,Spike_Y145del)
EPI_ISL_12280350	14/04/2022	BA.2	BA.2	GRA	(NSP5_P132H,NSP3_G489S,Spike_L24del,NSP4_T327I,Spike_S373P,Spike_N969K,Spike_H655Y,N_R203K,Spike_V213G,Spike_A27S,Spike_Q954H,N_P13L,Spike_P25del,N_R32del,NS3_T223I,Spike_T19I,M_Q19E,Spike_N440K,NSP4_T492I,NSP3_D853G,Spike_N679K,Spike_N764K,NSP6_G107del,N_G204R,M_A63T,Spike_S371F,Spike_K417N,NSP13_R392C,Spike_T376A,NSP6_S106del,NS3_V97I,Spike_G339D,Spike_R408S,NSP4_L438F,NSP4_L264F,Spike_P681H,NSP3_T24I,N_S33del,NSP1_S135R,Spike_S375F,Spike_D405N,N_E31del,NSP15_T112I,NSP6_F108del,NSP3_T678I,Spike_P26del,NSP12_P323L)
EPI_ISL_12280348	15/04/2022	BA.1	BA.1.17	GRA	(NSP5_P132H,Spike_H69del,Spike_T95I,Spike_A67V,Spike_S373P,Spike_N969K,Spike_H655Y,Spike_N856K,N_R203K,Spike_G142D,NSP2_P129L,Spike_Q954H,N_P13L,NSP3_L1266I,N_R32del,M_Q19E,Spike_N440K,NSP4_T492I,NSP6_L105del,Spike_N679K,Spike_N764K,Spike_L212I,NSP6_G107del,NSP6_I189V,Spike_T547K,M_D3G,N_G204R,NSP14_V510I,Spike_V143del,M_A63T,NSP3_V1069I,Spike_K417N,NSP6_S106del,Spike_S371L,Spike_G339D,NSP3_S1265del,NSP14_I42V,Spike_P681H,Spike_Y144del,Spike_ins214EPE,N_S33del,Spike_S375F,Spike_G446S,NSP3_P822L,N_S21T,N_E31del,NSP3_K38R,Spike_N211del,E_T9I,Spike_V70del,NSP12_P323L,Spike_Y145del,Spike_D614G)
EPI_ISL_12280349	15/04/2022	BA.2	BA.2	GRA	(NSP5_P132H,Spike_S371F,NSP3_G489S,NSP13_R392C,Spike_K417N,Spike_L24del,NSP4_T327I,Spike_S373P,NSP6_S106del,Spike_T376A,Spike_N969K,Spike_H655Y,N_R203K,Spike_V213G,Spike_A27S,Spike_Q954H,Spike_G339D,N_P13L,Spike_R408S,Spike_P25del,N_R32del,NSP4_L438F,NS3_T223I,Spike_T19I,NSP4_L264F,Spike_P681H,M_Q19E,Spike_N440K,N_S33del,NSP1_S135R,NSP4_T492I,Spike_S375F,Spike_D405N,NS7a_T120K,Spike_N679K,Spike_N764K,NSP6_G107del,N_E31del,NSP15_T112I,NSP6_F108del,N_G204R,N_S413R,Spike_P26del,M_A63T,NSP12_P323L)

EPI_ISL_12323363	15/04/2022	BA.2	BA.2.3.15	GRA	(NSP5_P132H,NS3_G254stop,NSP3_G489S,Spike_L24del,NSP4_T327I,Spike_S373P,Spike_Q493R,Spike_N969K,Spike_H655Y,N_R203K,Spike_V213G,Spike_G142D,Spike_A27S,Spike_Q954H,N_P13L,Spike_P25del,N_R32del,NS3_T223I,Spike_T19I,M_Q19E,Spike_N440K,NSP4_T492I,Spike_N679K,Spike_N764K,NSP6_G107del,N_G204R,Spike_T478K,N_S413R,M_A63T,Spike_S371F,Spike_K417N,NSP13_R392C,Spike_T376A,NSP6_S106del,Spike_R408S,NSP4_L438F,NSP4_A146V,Spike_P681H,NSP4_L264F,NSP3_T24I,N_S33del,NSP1_S135R,Spike_S375F,Spike_D405N,Spike_D215E,Spike_S477N,N_E31del,NSP15_T112I,NSP6_F108del,Spike_E484A,E_T9I,Spike_P26del,NSP12_P323L,Spike_D614G)
EPI_ISL_12323364	15/04/2022	BA.2	BA.2.3.15	GRA	(NSP5_P132H,NSP3_G489S,Spike_L24del,NSP4_T327I,Spike_S373P,Spike_Q493R,Spike_N969K,Spike_H655Y,N_R203K,Spike_V213G,Spike_G142D,Spike_A27S,Spike_Q954H,N_P13L,Spike_P25del,N_R32del,NS3_T223I,Spike_T19I,M_Q19E,Spike_N440K,NSP4_T492I,Spike_N679K,Spike_N764K,NSP6_G107del,NS7a_G26S,N_G204R,Spike_T478K,N_S413R,M_A63T,Spike_S371F,Spike_K417N,NSP13_R392C,Spike_T376A,NSP6_S106del,Spike_R408S,NSP4_L438F,NSP4_A146V,Spike_P681H,NSP4_L264F,NSP3_T24I,N_S33del,NSP1_S135R,Spike_S375F,Spike_D405N,Spike_D215E,Spike_S477N,N_E31del,NSP15_T112I,NSP6_F108del,Spike_E484A,Spike_P26del,NSP12_P323L,Spike_D614G)
EPI_ISL_12323373	16/04/2022	BA.2	BA.2	GRA	(NSP5_P132H,NSP3_G489S,Spike_L24del,NSP4_T327I,Spike_S373P,Spike_N969K,Spike_H655Y,N_R203K,Spike_V213G,Spike_G142D,Spike_A27S,Spike_Q954H,N_P13L,Spike_P25del,N_R32del,NS3_T223I,Spike_T19I,M_Q19E,Spike_N440K,NSP4_T492I,Spike_N679K,Spike_N764K,NSP6_G107del,NSP3_T725I,N_G204R,Spike_T478K,N_S413R,M_A63T,Spike_S371F,Spike_K417N,NSP13_R392C,Spike_T376A,NSP6_S106del,Spike_R408S,NSP4_L438F,Spike_P681H,NSP4_L264F,NSP3_T24I,N_S33del,NSP1_S135R,Spike_S375F,Spike_D405N,Spike_S477N,N_E31del,NSP15_T112I,NSP6_F108del,Spike_E484A,Spike_P26del,NSP12_P323L,Spike_D614G)
EPI_ISL_12323374	16/04/2022	BA.2	BA.2	GRA	(NSP5_P132H,NSP3_G489S,Spike_L24del,NSP4_T327I,Spike_S373P,Spike_N969K,Spike_H655Y,N_R203K,Spike_V213G,Spike_G142D,Spike_A27S,Spike_Q954H,N_P13L,Spike_P25del,N_R32del,NS3_T223I,Spike_T19I,M_Q19E,Spike_N440K,NSP4_T492I,Spike_N679K,Spike_N764K,NSP6_G107del,N_G204R,Spike_T478K,N_S413R,M_A63T,Spike_S371F,Spike_K417N,NSP13_R392C,Spike_T376A,NSP6_S106del,Spike_R408S,NSP4_L438F,Spike_P681H,NSP4_L264F,NSP3_T24I,N_S33del,NSP1_S135R,Spike_S375F,Spike_D405N,Spike_S477N,N_E31del,NSP15_T112I,NSP6_F108del,Spike_E484A,Spike_P26del,Spike_A522P,NSP12_P323L,Spike_D614G)
EPI_ISL_12323375	16/04/2022	BA.1	BA.1	GRA	(NSP5_P132H,Spike_H69del,Spike_T95I,Spike_A67V,NSP3_G489S,Spike_K417N,Spike_S373P,NSP6_S106del,Spike_N969K,Spike_H655Y,N_R203K,Spike_S371L,Spike_G142D,Spike_Q954H,N_P13L,N_R32del,Spike_P681H,Spike_Y144del,Spike_ins214EPE,M_Q19E,Spike_N440K,N_S33del,NSP4_T492I,Spike_S375F,NSP6_L105del,Spike_N679K,Spike_N764K,Spike_L212I,NSP6_G107del,N_E31del,Spike_N211del,Spike_R346K,NSP6_I189V,Spike_T547K,M_D3G,N_G204R,E_T9I,Spike_V70del,Spike_V143del,M_A63T,NSP12_P323L,Spike_Y145del,Spike_D614G)
EPI_ISL_12323371	17/04/2022	BA.2	BA.2	GRA	(NSP5_P132H,NSP3_G489S,Spike_L24del,NSP4_T327I,Spike_S373P,Spike_N969K,Spike_H655Y,N_R203K,Spike_V213G,Spike_G142D,Spike_A27S,Spike_Q954H,N_P13L,Spike_P25del,N_R32del,NS3_T223I,Spike_T19I,M_Q19E,Spike_N440K,NSP4_T492I,Spike_N679K,Spike_N764K,NSP6_G107del,N_G204R,Spike_T478K,N_S413R,M_A63T,Spike_S371F,Spike_K417N,NSP13_R392C,Spike_T376A,NSP6_S106del,Spike_R408S,NSP4_L438F,Spike_P681H,NSP4_L264F,NSP3_T24I,N_S33del,NSP1_S135R,Spike_S375F,Spike_D405N,Spike_S477N,NSP3_G1273D,N_E31del,NSP15_T112I,NSP6_F108del,Spike_E484A,Spike_P26del,NSP12_P323L,Spike_D614G)
EPI_ISL_13535785	18/05/2022	BA.5	BA.5.1	GRA	(NSP5_P132H,Spike_H69del,NSP3_G489S,Spike_L24del,NSP4_T327I,Spike_S373P,Spike_N969K,Spike_H655Y,N_R203K,Spike_V213G,Spike_G142D,Spike_A27S,Spike_Q954H,N_P13L,Spike_P25del,N_R32del,NS3_T223I,Spike_T19I,M_Q19E,Spike_N440K,NSP4_T492I,M_D3N,Spike_N679K,Spike_N764K,NSP6_G107del,N_G204R,Spike_T478K,N_S413R,M_A63T,Spike_S371F,Spike_K417N,NSP13_R392C,Spike_F486V,Spike_T376A,NSP6_S106del,NSP14_G17W,NSP6_K270R,Spike_L5F,Spike_R408S,Spike_P681H,NSP4_L264F,NSP3_T24I,N_S33del,NSP1_S135R,Spike_S375F,Spike_D405N,Spike_S477N,NS3_P42L,N_E31del,NSP15_T112I,NSP6_F108del,Spike_E484A,E_T9I,Spike_V70del,Spike_P26del,NSP12_P323L,Spike_D614G,Spike_L452R)
EPI_ISL_13479631	09/06/2022	BA.5	BA.5.1	GR	(NSP5_P132H,Spike_H69del,NSP3_G489S,Spike_L24del,NSP4_T327I,Spike_S373P,Spike_N969K,Spike_H655Y,N_R203K,Spike_V213G,Spike_G142D,Spike_A27S,Spike_Q954H,N_P13L,Spike_P25del,N_R32del,NS3_T223I,Spike_T19I,M_Q19E,Spike_N440K,NSP4_T492I,M_D3N,Spike_N679K,Spike_N764K,NSP6_G107del,N_G204R,N_S413R,M_A63T,Spike_S371F,Spike_K417N,NSP13_R392C,Spike_T376A,NSP6_S106del,Spike_R408S,Spike_P681H,NSP4_L264F,NSP3_T24I,N_S33del,NSP1_S135R,Spike_S375F,Spike_D405N,N_E31del,NSP15_T112I,NSP6_F108del,E_T9I,Spike_V70del,Spike_P26del,NSP12_P323L,Spike_D614G,Spike_L452R)
EPI_ISL_13479630	10/06/2022	BA.4	BA.4	GR	(NSP5_P132H,Spike_H69del,NS6_E55Y,NSP3_G489S,Spike_L24del,NSP4_T327I,Spike_S373P,Spike_N969K,Spike_H655Y,N_R203K,Spike_V213G,Spike_G142D,Spike_A27S,Spike_Q954H,N_P13L,Spike_P25del,N_R32del,NS3_T223I,Spike_T19I,NSP3_T350I,M_Q19E,Spike_N440K,NSP4_T492I,Spike_S640F,Spike_N679K,Spike_N764K,NSP6_G107del,N_G204R,N_S413R,M_A63T,Spike_S371F,NS7b_L11F,N_P151S,Spike_K417N,NSP13_R392C,Spike_T376A,NSP6_S106del,Spike_R408S,NSP4_L264F,Spike_P681H,NSP3_T24I,N_S33del,Spike_S375F,NSP1_S135R,Spike_D405N,NSP1_F143del,NSP1_K141del,N_E31del,NSP1_S142del,NSP15_T112I,NSP6_F108del,E_T9I,Spike_V70del,Spike_P26del,NSP12_P323L,Spike_D614G,Spike_L452R)
EPI_ISL_13479632	10/06/2022	BA.4	BA.4	GR	(NSP5_P132H,Spike_H69del,NS6_E55Y,NSP3_G489S,Spike_L24del,NSP4_T327I,Spike_S373P,Spike_N969K,Spike_H655Y,N_R203K,Spike_V213G,Spike_G142D,Spike_A27S,Spike_Q954H,N_P13L,Spike_P25del,N_R32del,NS3_T223I,Spike_T19I,NSP3_T350I,M_Q19E,Spike_N440K,NSP4_T492I,Spike_S640F,Spike_N679K,Spike_N764K,NSP6_G107del,N_G204R,N_S413R,M_A63T,Spike_S371F,NS7b_L11F,N_P151S,Spike_K417N,NSP13_R392C,Spike_T376A,NSP6_S106del,Spike_R408S,NSP4_L264F,Spike_P681H,NSP3_T24I,N_S33del,Spike_S375F,NSP1_S135R,Spike_D405N,NSP1_F143del,NSP1_K141del,N_E31del,NSP1_S142del,NSP15_T112I,NSP6_F108del,E_T9I,Spike_V70del,Spike_P26del,NSP12_P323L,Spike_D614G,Spike_L452R)
EPI_ISL_13479633	10/06/2022	BA.2	BA.2	GRA	(NSP5_P132H,NSP3_G489S,Spike_L24del,NSP4_T327I,Spike_S373P,Spike_N969K,Spike_H655Y,N_R203K,Spike_V213G,Spike_G142D,Spike_A27S,Spike_Q954H,N_P13L,Spike_P25del,N_R32del,NS3_T223I,Spike_T19I,M_Q19E,Spike_N440K,NSP4_T492I,NS3_G100C,Spike_N679K,Spike_N764K,NSP6_G107del,N_G204R,Spike_T478K,N_S413R,M_A63T,Spike_S371F,Spike_K417N,NSP13_R392C,Spike_T376A,NSP6_S106del,Spike_R408S,NSP4_L438F,Spike_P681H,NSP4_L264F,N_S33del,Spike_S375F,NSP1_S135R,Spike_D405N,Spike_S477N,N_E31del,NSP15_T112I,NSP6_F108del,Spike_E484A,Spike_P26del,NSP12_P323L,Spike_D614G)
EPI_ISL_13479627	11/06/2022	BA.4	BA.4	GR	(NSP5_P132H,Spike_H69del,Spike_N658S,NSP3_A264V,NSP3_G489S,Spike_L24del,NSP4_T327I,Spike_S373P,Spike_H655Y,Spike_N969K,N_R203K,Spike_V213G,Spike_G142D,Spike_A27S,Spike_Q954H,N_P13L,Spike_P25del,N_R32del,NS3_T223I,Spike_T19I,M_Q19E,Spike_N440K,NS8_P36S,NSP4_T492I,Spike_N679K,Spike_N764K,NSP6_G107del,N_G204R,N_S413R,M_A63T,Spike_S371F,NS7b_L11F,NSP10_T12I,N_P151S,Spike_K417N,NSP13_R392C,Spike_T376A,NSP6_S106del,NS3_V273L,Spike_R408S,NSP4_L264F,Spike_P681H,NSP3_T24I,N_S33del,NSP1_S135R,Spike_S375F,Spike_D405N,NSP1_F143del,NSP1_K141del,N_E31del,NSP1_S142del,NSP15_T112I,NSP6_F108del,E_T9I,Spike_V70del,Spike_P26del,NSP12_P323L,Spike_D614G,Spike_L452R)

EPI_ISL_13479629	11/06/2022	BA.2	BA.2.12.1	GRA	(NSP5_P132H,NSP3_G489S,Spike_L24del,NSP4_T327I,Spike_S373P,Spike_N969K,Spike_H655Y,N_R203K,Spike_V213G,Spike_G142D,Spike_A27S,Spike_Q954H,N_P13L,Spike_P25del,N_R32del,NS3_T223I,Spike_T19I,M_Q19E,Spike_N440K,NSP4_T492I,Spike_N679K,Spike_N764K,NSP6_G107del,N_G204R,Spike_T478K,N_S413R,M_A63T,Spike_S371F,Spike_K417N,NSP13_R392C,NS8_Q18stop,Spike_T376A,NSP6_S106del,Spike_R408S,NSP4_L438F,NSP4_L264F,Spike_P681H,NSP3_T24I,N_S33del,NSP1_S135R,Spike_S375F,Spike_D405N,Spike_S477N,N_E31del,NSP15_T112I,NSP6_F108del,Spike_E484A,E_T9I,Spike_P26del,NSP6_F184V,NSP12_P323L,Spike_S704L,N_A152S,Spike_D614G,NS7b_A15S,Spike_L452Q,NSP12_V473I)
EPI_ISL_13479628	13/06/2022	BA.2	BA.2.9	GRA	(NSP5_P132H,NSP3_G489S,Spike_L24del,NSP4_T327I,Spike_S373P,Spike_N969K,Spike_H655Y,N_R203K,Spike_V213G,Spike_G142D,NSP2_A518V,Spike_A27S,Spike_Q954H,N_P13L,Spike_P25del,N_R32del,NS3_T223I,Spike_T19I,M_Q19E,Spike_N440K,NSP4_T492I,Spike_N679K,Spike_N764K,NSP6_G107del,N_G204R,Spike_T478K,N_S413R,M_A63T,Spike_S371F,NS3_H78Y,Spike_K417N,NSP13_R392C,Spike_T376A,NSP6_S106del,NSP4_A231V,Spike_R408S,NSP4_L438F,Spike_P681H,NSP4_L264F,NSP3_T24I,N_S33del,NSP1_S135R,Spike_S375F,Spike_D405N,N_T334I,Spike_S477N,N_E31del,Spike_A1020V,NSP15_T112I,NSP6_F108del,Spike_E484A,Spike_M153I,E_T9I,Spike_P26del,NSP12_P323L,Spike_D614G)
EPI_ISL_13479716	13/06/2022	BA.2	BA.2	GRA	(NSP5_P132H,NSP3_G489S,Spike_L24del,NSP4_T327I,Spike_S373P,Spike_Q493R,Spike_N969K,Spike_H655Y,N_R203K,Spike_V213G,Spike_G142D,Spike_A27S,Spike_Q954H,N_P13L,Spike_P25del,N_R32del,NS3_T223I,Spike_T19I,M_Q19E,Spike_N440K,NSP4_T492I,Spike_N679K,Spike_N764K,NSP6_G107del,N_G204R,Spike_T478K,N_S413R,M_A63T,Spike_S371F,Spike_K417N,NSP13_R392C,Spike_T376A,NSP6_S106del,Spike_R408S,NSP4_L438F,Spike_P681H,NSP4_L264F,NSP3_T24I,N_S33del,NSP1_S135R,Spike_S375F,Spike_D405N,Spike_S477N,N_E31del,NSP15_T112I,NSP6_F108del,Spike_E484A,Spike_P26del,NSP12_P323L,Spike_D614G)
EPI_ISL_13479718	13/06/2022	BA.2	BA.2.56	GRA	(NSP5_P132H,NSP3_G489S,Spike_L24del,NSP4_T327I,Spike_S373P,Spike_N969K,Spike_H655Y,N_R203K,Spike_V213G,Spike_G142D,Spike_A27S,Spike_Q954H,N_P13L,Spike_P25del,N_R32del,NS3_T223I,Spike_T19I,M_Q19E,Spike_N440K,NSP4_T492I,Spike_N679K,Spike_N764K,NSP6_G107del,NSP3_T725I,N_G204R,Spike_T478K,N_S413R,M_A63T,Spike_S371F,Spike_K417N,NSP13_R392C,Spike_T376A,NSP6_S106del,Spike_R408S,NSP4_L438F,Spike_P681H,NSP4_L264F,NSP3_T24I,N_S33del,NSP1_S135R,Spike_S375F,Spike_D405N,Spike_L452M,Spike_S477N,N_E31del,NSP15_T112I,NSP6_F108del,Spike_E484A,Spike_P26del,NSP12_P323L,Spike_D614G)
EPI_ISL_13479721	13/06/2022	BA.4	BA.4	GR	(NSP5_P132H,Spike_H69del,Spike_N658S,NSP3_G489S,Spike_L24del,NSP4_T327I,Spike_S373P,Spike_N969K,Spike_H655Y,N_R203K,Spike_V213G,Spike_G142D,Spike_A27S,Spike_Q954H,N_P13L,Spike_P25del,N_R32del,NS3_T223I,Spike_T19I,M_Q19E,Spike_N440K,NSP4_T492I,Spike_N679K,Spike_N764K,NSP6_G107del,NSP2_T573I,N_G204R,N_S413R,M_A63T,Spike_S371F,NS7b_L11F,N_P151S,Spike_K417N,NSP13_R392C,Spike_T376A,NSP6_S106del,Spike_R408S,Spike_P681H,NSP4_L264F,N_S33del,NSP1_S135R,Spike_S375F,Spike_D405N,NSP1_F143del,NSP1_K141del,N_E31del,NSP1_S142del,NSP15_T112I,NSP6_F108del,Spike_V70del,Spike_P26del,NSP12_P323L,Spike_D614G,Spike_L452R)
EPI_ISL_13479723	13/06/2022	BA.5	BA.5.1	GRA	(NSP5_P132H,Spike_H69del,NSP3_G489S,Spike_L24del,NSP4_T327I,Spike_S373P,Spike_N969K,Spike_H655Y,N_R203K,Spike_V213G,Spike_G142D,Spike_A27S,Spike_Q954H,N_P13L,NSP3_M953T,Spike_P25del,N_R32del,NS3_T223I,Spike_T19I,M_Q19E,Spike_N440K,NS8_P36S,NSP4_T492I,M_D3N,Spike_N679K,Spike_N764K,NSP6_G107del,N_G204R,N_S413R,M_A63T,Spike_S371F,Spike_K417N,NSP13_R392C,Spike_F486V,Spike_T376A,NSP6_S106del,Spike_R408S,Spike_P681H,NSP4_L264F,NSP3_T24I,N_S33del,NSP1_S135R,Spike_S375F,Spike_D405N,N_E31del,NSP15_T112I,NSP6_F108del,Spike_E484A,Spike_V70del,Spike_P26del,NSP12_P323L,Spike_D614G,Spike_L452R)
EPI_ISL_13535752	13/06/2022	BA.2	BA.2	GRA	(NSP5_P132H,NSP3_G489S,Spike_L24del,NSP4_T327I,Spike_S373P,Spike_N969K,Spike_H655Y,N_R203K,Spike_V213G,Spike_G142D,Spike_A27S,Spike_Q954H,N_P13L,Spike_P25del,N_R32del,NS3_T223I,Spike_T19I,M_Q19E,Spike_N440K,NSP4_T492I,Spike_N679K,Spike_N764K,NSP6_G107del,N_G204R,Spike_T478K,N_S413R,M_A63T,Spike_S371F,Spike_K417N,NSP13_R392C,Spike_T376A,NSP6_S106del,Spike_R408S,NSP4_L438F,Spike_P681H,NSP4_L264F,NSP3_T24I,N_S33del,NSP1_S135R,Spike_S375F,Spike_D405N,Spike_S477N,N_E31del,NSP15_T112I,NSP6_F108del,Spike_E484A,E_T9I,Spike_P26del,NSP12_P323L,Spike_D614G)
EPI_ISL_13535754	13/06/2022	BA.2	BA.2.37	GRA	(NSP5_P132H,NSP3_G489S,Spike_L24del,NSP4_T327I,Spike_S373P,Spike_N969K,Spike_H655Y,N_R203K,Spike_V213G,Spike_G142D,Spike_A27S,Spike_Q954H,N_P13L,Spike_P25del,N_R32del,NS3_T223I,Spike_T19I,M_Q19E,Spike_N440K,NSP4_T492I,NSP10_T51I,Spike_N679K,Spike_N764K,NSP6_G107del,N_G204R,Spike_T478K,N_S413R,M_A63T,Spike_S371F,NS8_Q27stop,Spike_K417N,NSP13_R392C,Spike_T376A,NSP6_S106del,Spike_R408S,NSP4_L438F,Spike_P681H,NSP4_L264F,NSP3_T24I,N_S33del,NSP1_S135R,Spike_S375F,Spike_D405N,Spike_S477N,NSP3_R603K,N_E31del,NSP6_F108del,Spike_E484A,E_T9I,Spike_P26del,NSP12_P323L,Spike_D614G)
EPI_ISL_13535759	13/06/2022	BA.2	BA.2.65	GR	(NSP5_P132H,NSP3_G489S,Spike_L24del,NSP4_T327I,Spike_S373P,Spike_H655Y,N_R203K,Spike_V213G,Spike_G142D,Spike_A27S,Spike_Q954H,N_P13L,Spike_P25del,N_R32del,NS3_T223I,Spike_T19I,M_Q19E,Spike_N440K,NSP4_T492I,NSP2_A476V,Spike_N679K,Spike_N764K,NSP6_G107del,N_G204R,N_S413R,M_A63T,Spike_S371F,Spike_K417N,NSP13_R392C,NSP7_M3I,Spike_T376A,NSP6_S106del,Spike_R408S,NSP4_L438F,Spike_P681H,NSP4_L264F,NSP3_T24I,N_S33del,NSP1_S135R,Spike_S375F,Spike_D405N,NSP2_R27C,N_E31del,NSP6_F108del,E_T9I,Spike_P26del,NSP12_P323L,Spike_D614G)
EPI_ISL_13535763	13/06/2022	BA.2	BA.2	GR	(NSP5_P132H,Spike_S371F,NSP3_G489S,NSP13_R392C,Spike_K417N,Spike_L24del,NSP4_T327I,Spike_S373P,NSP6_S106del,Spike_T376A,Spike_H655Y,N_R203K,Spike_V213G,Spike_G142D,Spike_A27S,Spike_Q954H,N_P13L,Spike_R408S,Spike_P25del,N_R32del,NSP4_L438F,NS3_T223I,Spike_T19I,NSP14_I42V,Spike_P681H,NSP4_L264F,M_Q19E,NSP3_T24I,Spike_N440K,N_S33del,NSP1_S135R,NSP4_T492I,Spike_S375F,Spike_D405N,Spike_N679K,Spike_N764K,NSP6_G107del,N_E31del,NSP6_F108del,N_G204R,E_T9I,N_S413R,Spike_P26del,M_A63T,NSP12_P323L,Spike_D614G)
EPI_ISL_13479621	14/06/2022	BA.5	BA.5.2	GR	(NSP5_P132H,Spike_H69del,NSP3_G489S,Spike_L24del,NSP4_T327I,Spike_S373P,Spike_N969K,Spike_H655Y,N_R203K,Spike_V213G,Spike_G142D,Spike_A27S,Spike_Q954H,N_P13L,Spike_P25del,N_R32del,NS3_T223I,Spike_T19I,M_Q19E,Spike_N440K,NSP4_T492I,M_D3N,Spike_N679K,Spike_N764K,NSP6_G107del,N_G204R,N_S413R,M_A63T,Spike_S371F,Spike_K417N,NSP13_R392C,Spike_T376A,NSP6_S106del,Spike_R408S,Spike_P681H,NSP4_L264F,NSP3_T24I,N_S33del,NSP1_S135R,Spike_S375F,Spike_D405N,N_E31del,NSP15_T112I,NSP6_F108del,Spike_V70del,Spike_P26del,NSP12_P323L,Spike_D614G,Spike_L452R)
EPI_ISL_13479623	14/06/2022	BA.4	BA.4.1	GRA	(NSP5_P132H,Spike_H69del,Spike_V3G,NSP3_G489S,Spike_L24del,NSP4_T327I,Spike_S373P,Spike_N969K,Spike_H655Y,N_R203K,Spike_V213G,Spike_G142D,Spike_A27S,Spike_Q954H,N_P13L,Spike_P25del,N_R32del,NS3_T223I,Spike_T19I,M_Q19E,Spike_N440K,NSP4_T492I,Spike_N679K,Spike_N764K,NSP6_G107del,N_G204R,Spike_T478K,N_S413R,M_A63T,Spike_S371F,NS7b_L11F,N_P151S,Spike_K417N,NSP13_R392C,Spike_F486V,Spike_T376A,NSP6_S106del,Spike_R408S,NSP4_L264F,Spike_P681H,NSP3_T24I,N_S33del,NSP1_S135R,Spike_S375F,NSP13_I79V,Spike_D405N,NSP1_F143del,Spike_S477N,NSP1_K141del,N_E31del,NSP1_S142del,NSP15_T112I,NSP6_F108del,Spike_E484A,E_T9I,Spike_V70del,Spike_P26del,NSP12_P323L,Spike_D614G,Spike_L452R)

EPI_ISL_13479624	14/06/2022	BA.2	BA.2	GR	(NSP5_P132H,Spike_H69del,NSP3_G489S,Spike_L24del,NSP4_T327I,Spike_S373P,Spike_N969K,Spike_H655Y,N_R203K,Spike_V213G,Spike_G142D,Spike_A27S,Spike_Q954H,N_P13L,Spike_P25del,N_R32del,NS3_T223I,Spike_T19I,M_Q19E,Spike_N440K,NSP4_T492I,Spike_S640F,Spike_N679K,Spike_N764K,NSP6_G107del,N_G204R,N_S413R,M_A63T,Spike_S371F,NS7b_L11F,N_P151S,Spike_K417N,NSP13_R392C,Spike_T376A,NSP6_S106del,Spike_R408S,Spike_P681H,NSP4_L264F,NSP3_T24I,N_S33del,Spike_S375F,NSP1_S135R,Spike_D405N,NSP1_F143del,NSP1_K141del,N_E31del,NSP1_S142del,NSP6_F108del,E_T9I,Spike_V70del,Spike_P26del,NSP12_P323L,Spike_D614G,Spike_L452R)
EPI_ISL_13479710	14/06/2022	BA.2	BA.2	GRA	(NSP5_P132H,NSP3_G489S,Spike_L24del,NSP4_T327I,Spike_S373P,Spike_Q493R,Spike_N969K,Spike_H655Y,N_R203K,Spike_V213G,Spike_G142D,Spike_A27S,Spike_Q954H,Spike_P251L,N_P13L,Spike_P25del,N_R32del,NS3_T223I,Spike_T19I,M_Q19E,Spike_N440K,NSP4_T492I,Spike_N679K,Spike_N764K,NSP6_G107del,N_G204R,Spike_T478K,N_S413R,M_A63T,NS7b_S5L,Spike_S371F,Spike_K417N,NSP13_R392C,Spike_T376A,NSP6_S106del,NSP2_R46K,Spike_G339D,Spike_R408S,NSP4_L438F,NSP4_L264F,Spike_P681H,NSP3_T24I,N_S33del,NSP1_S135R,Spike_S375F,Spike_D405N,Spike_S477N,N_E31del,NSP15_T112I,NSP6_F108del,Spike_E484A,Spike_P26del,NSP12_P323L,Spike_D614G)
EPI_ISL_13479712	14/06/2022	BA.2	BA.2.9	GRA	(NSP5_P132H,N_T135I,NSP2_L462F,NSP3_G489S,Spike_L24del,NSP4_T327I,NSP3_P1103L,Spike_S373P,Spike_Q493R,Spike_N969K,Spike_H655Y,N_R203K,Spike_V213G,Spike_G142D,Spike_A27S,Spike_Q954H,N_P13L,Spike_P25del,N_R32del,NS3_T223I,Spike_T19I,M_Q19E,Spike_N440K,NSP4_T492I,NSP13_S38P,Spike_N679K,Spike_N764K,NSP6_G107del,N_G204R,Spike_T478K,N_S413R,M_A63T,NSP2_E467G,Spike_S371F,NS8_Q27stop,NS3_H78Y,Spike_K417N,NSP13_R392C,Spike_T376A,NSP6_S106del,Spike_G339D,Spike_R408S,NSP4_L438F,NSP4_L264F,Spike_P681H,NSP3_T24I,N_S33del,NSP1_S135R,Spike_S375F,Spike_D405N,Spike_S477N,N_E31del,NSP15_T112I,NSP6_F108del,Spike_E484A,Spike_P26del,NSP12_P323L,Spike_D614G)
EPI_ISL_13479715	14/06/2022	BA.5	BA.5.1	GRA	(NSP5_P132H,Spike_H69del,NSP3_G489S,Spike_L24del,NSP4_T327I,Spike_S373P,Spike_N969K,Spike_H655Y,N_R203K,Spike_V213G,Spike_G142D,Spike_A27S,Spike_Q954H,N_P13L,Spike_P25del,N_R32del,NS3_T223I,Spike_T19I,M_Q19E,Spike_N440K,NSP4_T492I,M_D3N,Spike_N679K,Spike_N764K,NSP6_G107del,N_G204R,Spike_T478K,N_S413R,M_A63T,Spike_S371F,Spike_K417N,NSP13_R392C,Spike_F486V,Spike_T376A,NSP6_S106del,Spike_R408S,Spike_P681H,NSP4_L264F,NSP3_T24I,N_S33del,NSP1_S135R,Spike_S375F,Spike_D405N,NSP10_H48Y,Spike_S477N,N_E31del,NSP2_D155A,NSP15_T112I,NSP6_F108del,Spike_E484A,Spike_V70del,Spike_P26del,NSP12_P323L,Spike_D614G,Spike_L452R)
EPI_ISL_13479719	14/06/2022	BA.2	BA.2.9	GRA	(NSP5_P132H,NSP3_G489S,Spike_L24del,NSP4_T327I,Spike_S373P,Spike_Q493R,Spike_N969K,Spike_H655Y,N_R203K,Spike_V213G,Spike_G142D,Spike_A27S,Spike_Q954H,N_P13L,Spike_P25del,N_R32del,NS3_T223I,Spike_T19I,M_Q19E,Spike_N440K,NSP4_T492I,Spike_N679K,Spike_N764K,NSP6_G107del,NSP6_V190G,N_G204R,Spike_T478K,N_S413R,M_A63T,Spike_S371F,NS3_H78Y,Spike_K417N,NSP13_R392C,Spike_T376A,NSP6_S106del,Spike_R408S,NSP4_L438F,NSP4_L264F,Spike_P681H,NSP3_T24I,N_S33del,NSP1_S135R,Spike_S375F,Spike_D405N,Spike_S477N,N_E31del,NSP3_A1461V,NSP15_T112I,NSP6_F108del,Spike_E484A,Spike_P26del,NSP12_P323L,Spike_D614G)
EPI_ISL_13479726	14/06/2022	BA.5	BA.5.1	GRA	(NSP5_P132H,NSP13_S350L,Spike_H69del,NSP3_G489S,Spike_L24del,NSP4_T327I,Spike_S373P,Spike_N969K,Spike_H655Y,N_R203K,Spike_V213G,Spike_G142D,Spike_A27S,Spike_Q954H,N_P13L,Spike_P25del,N_R32del,NS3_T223I,Spike_T19I,M_Q19E,Spike_N440K,NSP4_T492I,M_D3N,Spike_N679K,Spike_N764K,NSP6_G107del,N_G204R,Spike_T478K,N_S413R,NSP9_T21I,M_A63T,Spike_S371F,Spike_K417N,NSP13_R392C,Spike_F486V,Spike_T376A,NSP6_S106del,Spike_R408S,NSP1_S135K,Spike_P681H,NSP4_L264F,NSP3_T24I,N_S33del,Spike_S375F,Spike_D405N,Spike_S477N,N_E31del,NSP15_T112I,NSP6_F108del,Spike_E484A,Spike_V70del,Spike_P26del,NSP12_P323L,Spike_D614G,Spike_L452R)
EPI_ISL_13479727	14/06/2022	BA.2	BA.2	GRA	(NSP5_P132H,NSP3_G489S,Spike_L24del,NSP4_T327I,Spike_S373P,Spike_N969K,Spike_H655Y,N_R203K,Spike_V213G,Spike_G142D,Spike_A27S,Spike_Q954H,N_P13L,Spike_P25del,N_R32del,NS3_T223I,Spike_T19I,M_Q19E,Spike_N440K,NSP4_T492I,Spike_N679K,Spike_N764K,NSP6_G107del,N_G204R,Spike_T478K,N_S413R,M_A63T,Spike_S371F,Spike_K417N,NSP13_R392C,Spike_T376A,NSP6_S106del,Spike_G339D,Spike_R408S,NSP4_L438F,Spike_P681H,NSP4_L264F,NSP3_T24I,N_S33del,NSP1_S135R,Spike_S375F,Spike_D405N,Spike_S477N,N_E31del,NSP15_T112I,NSP6_F108del,Spike_E484A,Spike_P26del,NSP12_P323L,Spike_D614G)
EPI_ISL_13535742	14/06/2022	BA.2	BA.2.52	GR	(NSP5_P132H,NSP3_G489S,Spike_L24del,NSP4_T327I,Spike_S373P,Spike_H655Y,N_R203K,Spike_V213G,Spike_G142D,Spike_A27S,Spike_Q954H,N_P13L,Spike_P25del,N_R32del,NS3_T223I,Spike_T19I,M_Q19E,Spike_N440K,NSP4_T492I,NSP2_C475R,Spike_ins248GEN,Spike_N679K,Spike_N764K,NSP6_G107del,N_G204R,N_S413R,M_A63T,Spike_S371F,Spike_K417N,NSP13_R392C,Spike_T376A,NSP6_S106del,Spike_R408S,NSP4_L438F,Spike_P681H,NSP4_L264F,NSP3_T24I,N_S33del,NSP1_S135R,Spike_S375F,Spike_D405N,NSP2_L550I,N_E31del,NSP6_F108del,E_T9I,Spike_P26del,NSP12_P323L,Spike_D614G,Spike_Y248S)
EPI_ISL_13535748	14/06/2022	BA.2	BA.2	GR	(NSP5_P132H,Spike_H69del,Spike_N658S,NSP3_G489S,Spike_L24del,NSP4_T327I,Spike_S373P,Spike_H655Y,N_R203K,Spike_V213G,Spike_G142D,Spike_A27S,Spike_Q954H,N_P13L,Spike_P25del,N_R32del,NS3_T223I,Spike_T19I,M_Q19E,Spike_N440K,NSP4_T492I,Spike_N679K,Spike_N764K,NSP6_G107del,Spike_D796Y,N_G204R,N_S413R,M_A63T,Spike_S371F,NS7b_L11F,N_P151S,Spike_K417N,NSP13_R392C,Spike_T376A,NSP6_S106del,Spike_R408S,Spike_P681H,NSP4_L264F,NSP3_T24I,N_S33del,NSP1_S135R,Spike_S375F,Spike_D405N,NSP1_F143del,NSP1_K141del,N_E31del,NSP1_S142del,NSP6_F108del,E_T9I,Spike_V70del,Spike_P26del,NSP12_P323L,Spike_D614G,Spike_L452R)
EPI_ISL_13535750	14/06/2022	BA.2	BA.2	GRA	(NSP5_P132H,NSP3_G489S,Spike_L24del,NSP4_T327I,Spike_S373P,Spike_H655Y,N_R203K,Spike_V213G,Spike_G142D,Spike_A27S,Spike_Q954H,N_P13L,Spike_P25del,N_R32del,NS3_T223I,Spike_T19I,M_Q19E,Spike_N440K,NSP4_T492I,Spike_N679K,Spike_N764K,NSP6_G107del,N_G204R,Spike_T478K,N_S413R,M_A63T,Spike_S371F,Spike_K417N,NSP13_R392C,Spike_T376A,NSP6_S106del,NSP5_N274S,Spike_R408S,NSP4_L438F,Spike_P681H,NSP4_L264F,NSP3_T24I,N_S33del,NSP1_S135R,Spike_S375F,Spike_D405N,Spike_S477N,N_E31del,NSP6_F108del,Spike_E484A,E_T9I,Spike_P26del,NSP12_P323L,Spike_D614G)
EPI_ISL_13535781	14/06/2022	BA.5	BA.5.1	GRA	(NSP5_P132H,Spike_H69del,NSP3_G489S,Spike_L24del,NSP4_T327I,Spike_S373P,Spike_N969K,Spike_H655Y,N_R203K,Spike_V213G,Spike_G142D,Spike_A27S,Spike_Q954H,N_P13L,Spike_P25del,N_R32del,NS3_T223I,Spike_T19I,M_Q19E,Spike_N440K,NSP4_T492I,M_D3N,Spike_N679K,Spike_N764K,NSP6_G107del,N_G204R,Spike_T478K,N_S413R,M_A63T,Spike_S371F,NSP2_T160I,Spike_K417N,NSP13_R392C,Spike_F486V,Spike_T376A,NSP6_S106del,Spike_R408S,Spike_P681H,NSP4_L264F,NSP3_T24I,N_S33del,NSP1_S135R,Spike_S375F,Spike_D405N,Spike_S477N,N_E31del,NSP15_T112I,NSP6_F108del,Spike_E484A,E_T9I,Spike_V70del,Spike_P26del,NSP12_P323L,Spike_D614G,Spike_L452R)

EPI_ISL_13535794	14/06/2022	BA.5	BA.5.3	GRA	(NSP5_P132H,Spike_H69del,NSP3_G489S,Spike_L24del,NSP4_T327I,Spike_S373P,Spike_N969K,Spike_H655Y,N_R203K,Spike_V213G,Spike_G142D,Spike_A27S,Spike_Q954H,N_P13L,Spike_P25del,N_R32del,NS3_T223I,Spike_T19I,M_Q19E,Spike_N440K,NSP4_T492I,M_D3N,Spike_N679K,Spike_N764K,NSP6_G107del,N_G204R,Spike_T478K,N_S413R,M_A63T,NSP2_Q376K,Spike_S371F,Spike_K417N,NSP13_R392C,Spike_F486V,Spike_T376A,NSP6_S106del,Spike_R408S,Spike_P681H,NSP4_L264F,NSP3_T24I,N_S33del,NSP1_S135R,Spike_S375F,Spike_D405N,Spike_S477N,N_E31del,NSP15_T112I,NSP6_F108del,Spike_E484A,E_T9I,Spike_V70del,Spike_P26del,NSP12_P323L,Spike_D614G,Spike_L452R)
EPI_ISL_13479619	15/06/2022	BA.2	BA.2	GRA	(NSP5_P132H,NSP3_G489S,Spike_L24del,NSP4_T327I,Spike_S373P,Spike_Q493R,Spike_N969K,Spike_H655Y,N_R203K,Spike_V213G,Spike_G142D,Spike_A27S,Spike_Q954H,N_P13L,Spike_P25del,N_R32del,NS3_T223I,Spike_T19I,M_Q19E,NSP4_T492I,Spike_N679K,Spike_N764K,NSP6_G107del,N_S193N,N_G204R,Spike_T478K,N_S413R,M_A63T,NSP16_K160R,Spike_S371F,NSP3_A1527T,Spike_K417N,NSP13_R392C,Spike_T376A,NSP6_S106del,Spike_G339D,Spike_R408S,Spike_E1144G,NSP4_L438F,NSP4_L264F,Spike_P681H,NSP3_T24I,N_S33del,NSP1_S135R,Spike_S375F,Spike_D405N,Spike_S477N,N_E31del,NSP15_T112I,NSP6_F108del,NSP3_P389S,Spike_E484A,Spike_P26del,NSP12_P323L,Spike_D614G)
EPI_ISL_13479620	15/06/2022	BA.2	BA.2	GRA	(NSP5_P132H,NSP3_G489S,Spike_L24del,NSP4_T327I,Spike_S373P,Spike_Q493R,Spike_N969K,Spike_H655Y,N_R203K,Spike_V213G,Spike_G142D,Spike_A27S,Spike_Q954H,N_P13L,Spike_F157S,Spike_P25del,N_R32del,NS3_T223I,Spike_T19I,M_Q19E,Spike_N440K,NSP4_T492I,Spike_N679K,Spike_N764K,NSP6_G107del,N_G204R,Spike_T478K,N_S413R,M_A63T,Spike_S371F,Spike_K417N,NSP13_R392C,Spike_T376A,NSP6_S106del,Spike_R408S,NSP4_L438F,Spike_P681H,NSP4_L264F,NSP3_T24I,N_S33del,NSP1_S135R,Spike_S375F,Spike_D405N,Spike_S477N,N_E31del,NSP15_T112I,NSP6_F108del,Spike_E484A,Spike_P26del,NSP12_P323L,Spike_D614G)
EPI_ISL_13479622	15/06/2022	BA.2	BA.2.3.15	GRA	(NSP5_P132H,NSP3_G489S,Spike_L24del,NSP4_T327I,Spike_S373P,Spike_Q493R,Spike_N969K,Spike_H655Y,N_R203K,Spike_V213G,Spike_G142D,Spike_A27S,Spike_Q954H,N_P13L,Spike_P25del,N_R32del,NS3_T223I,Spike_T19I,M_Q19E,Spike_N440K,NSP4_T492I,Spike_N679K,Spike_N764K,NSP6_G107del,N_G204R,Spike_T478K,N_S413R,NSP2_Q275R,M_A63T,Spike_S371F,Spike_K417N,NSP13_R392C,Spike_T376A,NSP6_S106del,Spike_G339D,Spike_R408S,NSP4_L438F,NSP4_A146V,NSP4_L264F,Spike_P681H,NSP3_T24I,N_S33del,NSP1_S135R,Spike_S375F,Spike_D405N,Spike_D215E,Spike_S477N,N_E31del,NSP15_T112I,NSP6_F108del,Spike_E484A,E_T9I,Spike_P26del,NSP12_P323L,Spike_D614G,NSP3_V281M)
EPI_ISL_13479635	15/06/2022	BA.4	BA.4	GR	(NSP5_P132H,Spike_H69del,Spike_N658S,NSP3_G489S,Spike_L24del,NSP4_T327I,Spike_S373P,Spike_N969K,Spike_H655Y,N_R203K,Spike_V213G,Spike_G142D,Spike_A27S,Spike_Q954H,N_P13L,Spike_P25del,N_R32del,NS3_T223I,Spike_T19I,M_Q19E,Spike_N440K,NSP4_T492I,Spike_N679K,Spike_N764K,NSP6_G107del,N_G204R,N_S413R,M_A63T,Spike_S371F,NS7b_L11F,N_P151S,Spike_K417N,NSP13_R392C,Spike_T376A,NSP6_S106del,Spike_R408S,NSP4_L264F,Spike_P681H,NSP3_T24I,N_S33del,NSP1_S135R,Spike_S375F,Spike_D405N,NSP1_F143del,NSP12_T806I,NSP1_K141del,N_E31del,NSP1_S142del,NSP15_T112I,NSP6_F108del,E_T9I,Spike_V70del,Spike_P26del,NSP12_P323L,Spike_D614G,Spike_L452R)
EPI_ISL_13479658	15/06/2022	BA.4	BA.4	GR	(NSP5_P132H,Spike_H69del,Spike_N658S,NSP3_G489S,Spike_L24del,NSP4_T327I,Spike_S373P,Spike_N969K,Spike_H655Y,N_R203K,Spike_V213G,Spike_G142D,Spike_A27S,Spike_Q954H,N_P13L,Spike_P25del,N_R32del,NS3_T223I,Spike_T19I,M_Q19E,Spike_N440K,NSP4_T492I,Spike_N679K,Spike_N764K,NSP6_G107del,N_G204R,N_S413R,M_A63T,Spike_S371F,NS7b_L11F,N_P151S,Spike_K417N,NSP13_R392C,Spike_T376A,NSP6_S106del,Spike_R408S,Spike_P681H,NSP4_L264F,NSP3_T24I,N_S33del,NSP1_S135R,Spike_S375F,Spike_D405N,NSP1_F143del,NSP1_K141del,N_E31del,NSP1_S142del,NSP15_T112I,NSP6_F108del,E_T9I,Spike_V70del,Spike_P26del,NSP12_P323L,Spike_D614G,Spike_L452R)
EPI_ISL_13479659	15/06/2022	BA.4	BA.4	GR	(NSP5_P132H,Spike_H69del,NSP3_G489S,Spike_L24del,NSP4_T327I,Spike_S373P,Spike_N969K,Spike_H655Y,N_R203K,Spike_V213G,Spike_G142D,Spike_A27S,Spike_Q954H,N_P13L,Spike_P25del,N_R32del,NS3_T223I,Spike_T19I,M_Q19E,Spike_N440K,NSP4_T492I,Spike_S640F,Spike_N679K,Spike_N764K,NSP6_G107del,N_G204R,N_S413R,M_A63T,Spike_S371F,NS7b_L11F,N_P151S,Spike_K417N,NSP13_R392C,Spike_T376A,NSP6_S106del,Spike_R408S,Spike_P681H,NSP4_L264F,NSP3_T24I,N_S33del,Spike_S375F,NSP1_S135R,Spike_D405N,NSP1_F143del,NSP1_K141del,N_E31del,NSP1_S142del,NSP15_T112I,NS6_E55D,NSP6_F108del,E_T9I,Spike_V70del,Spike_P26del,NSP12_P323L,Spike_D614G,Spike_L452R)
EPI_ISL_13479661	15/06/2022	BA.2	BA.2	GRA	(NSP5_P132H,NSP3_G489S,Spike_L24del,NSP4_T327I,Spike_S373P,Spike_N969K,Spike_H655Y,N_R203K,Spike_V213G,Spike_G142D,Spike_A27S,Spike_Q954H,N_P13L,Spike_P25del,N_R32del,NS3_T223I,Spike_T19I,M_Q19E,Spike_N440K,NSP4_T492I,Spike_N679K,Spike_N764K,NSP6_G107del,N_G204R,Spike_T478K,N_S413R,M_A63T,Spike_S371F,Spike_K417N,NSP13_R392C,Spike_T376A,NSP6_S106del,Spike_R408S,NSP4_L438F,Spike_P681H,NSP4_L264F,NSP3_T24I,N_S33del,NSP1_S135R,Spike_S375F,Spike_D405N,NSP3_S126L,Spike_S477N,N_E31del,NSP15_T112I,NSP6_F108del,Spike_E484A,Spike_P26del,NSP12_P323L,Spike_D614G)
EPI_ISL_13479707	15/06/2022	BA.2	BA.2.40.1	GRA	(NSP5_P132H,NSP3_G489S,Spike_L24del,NSP4_T327I,Spike_S373P,Spike_N969K,Spike_H655Y,N_R203K,Spike_V213G,Spike_G142D,Spike_A27S,Spike_Q954H,N_P13L,Spike_P25del,N_R32del,NS3_T223I,Spike_T19I,M_Q19E,Spike_N440K,NSP4_T492I,Spike_N679K,E_F26L,Spike_N764K,NSP6_G107del,N_G204R,Spike_T478K,N_S413R,M_A63T,Spike_S371F,NSP13_R392C,Spike_T376A,NSP6_S106del,Spike_K417T,Spike_R408S,NSP4_L438F,NSP4_L264F,Spike_P681H,NSP3_T24I,N_S33del,NSP1_S135R,Spike_S375F,Spike_D405N,NSP3_S211I,Spike_S477N,N_E31del,NSP15_T112I,NSP6_F108del,Spike_E484A,Spike_P26del,NSP12_P323L,NSP3_P389L,Spike_D614G)
EPI_ISL_13479708	15/06/2022	BA.2	BA.2.44	GRA	(NSP5_P132H,NSP3_G489S,Spike_L24del,NSP4_T327I,Spike_S373P,Spike_N969K,Spike_H655Y,N_R203K,Spike_V213G,Spike_G142D,Spike_A27S,Spike_Q954H,N_P13L,Spike_P25del,N_R32del,NS3_T223I,Spike_T19I,M_Q19E,Spike_N440K,NSP4_T492I,Spike_N679K,NSP6_G107del,N_G204R,Spike_T478K,N_S413R,M_A63T,Spike_K417N,NSP13_R392C,Spike_T376A,NSP6_S106del,Spike_R408S,NSP4_L438F,Spike_P681H,NSP4_L264F,NSP3_T24I,N_S33del,NSP1_S135R,Spike_S375F,Spike_D405N,NSP1_T12I,Spike_S477N,N_E31del,NSP15_T112I,NSP6_F108del,Spike_E484A,Spike_S371Y,Spike_P26del,NSP12_P323L,Spike_D614G)
EPI_ISL_13479711	15/06/2022	BA.4	BA.4.1	GRA	(NSP5_P132H,Spike_H69del,Spike_V3G,NSP3_G489S,Spike_L24del,NSP4_T327I,Spike_S373P,Spike_N969K,Spike_H655Y,N_R203K,Spike_V213G,Spike_G142D,Spike_A27S,Spike_Q954H,N_P13L,Spike_P25del,N_R32del,NS3_T223I,Spike_T19I,M_Q19E,Spike_N440K,NSP4_T492I,Spike_N679K,Spike_N764K,NSP6_G107del,N_G204R,N_S413R,M_A63T,Spike_S371F,NS7b_L11F,N_P151S,Spike_K417N,NSP13_R392C,Spike_T376A,NSP6_S106del,Spike_G339D,Spike_R408S,NSP4_L264F,Spike_P681H,N_S33del,NSP1_S135R,Spike_S375F,NSP13_I79V,Spike_D405N,NSP1_F143del,NSP1_K141del,N_E31del,NSP1_S142del,NSP15_T112I,NSP6_F108del,Spike_V70del,Spike_P26del,NSP12_P323L,Spike_D614G,Spike_L452R)
EPI_ISL_13479610	16/06/2022	BA.5	BA.5.3.1	GRA	(NSP5_P132H,Spike_H69del,NSP3_G489S,Spike_L24del,NSP4_T327I,Spike_S373P,Spike_N969K,Spike_H655Y,N_R203K,Spike_V213G,Spike_G142D,Spike_A27S,Spike_Q954H,N_P13L,Spike_P25del,N_R32del,NS3_T223I,Spike_T19I,M_Q19E,Spike_N440K,NSP4_T492I,N_E136D,M_D3N,Spike_N679K,Spike_N764K,NSP6_G107del,N_G204R,Spike_T478K,N_S413R,NSP6_L260F,M_A63T,NSP2_Q376K,Spike_S371F,Spike_K417N,NSP13_R392C,Spike_F486V,Spike_T376A,NSP6_S106del,Spike_R408S,Spike

					_P681H,NSP4_L264F,NSP3_T24I,N_S33del,NSP1_S135R,Spike_S375F,Spike_D405N,Spike_S477N,N_E31del,NSP15_T112I,NSP6_F108del,Spike_E484A,Spike_V70del,Spike_P26del,NSP12_P323L,Spike_D614G,Spike_L452R)
EPI_ISL_13479611	16/06/2022	BA.4	BA.4	GRA	(NSP5_P132H,Spike_H69del,Spike_N658S,NSP3_G489S,Spike_L24del,NSP4_T327I,Spike_S373P,Spike_N969K,Spike_H655Y,N_R203K,Spike_V213G,Spike_G142D,Spike_A27S,Spike_Q954H,N_P13L,Spike_P25del,N_R32del,NS3_T223I,Spike_T19I,M_Q19E,Spike_N440K,NSP4_T492I,Spike_N679K,Spike_N764K,NSP6_G107del,N_G204R,Spike_T478K,N_S413R,M_A63T,Spike_S371F,NS7b_L11F,N_P151S,Spike_K417N,NSP13_R392C,Spike_F486V,Spike_T376A,NSP6_S106del,Spike_R408S,NSP4_L264F,Spike_P681H,NSP3_T24I,N_S33del,NSP1_S135R,Spike_S375F,Spike_D405N,NSP1_F143del,Spike_S477N,NSP1_K141del,N_E31del,NSP1_S142del,NSP15_T112I,NSP6_F108del,Spike_E484A,E_T9I,Spike_V70del,Spike_P26del,NSP12_P323L,Spike_D614G,Spike_L452R)
EPI_ISL_13479612	16/06/2022	BA.5	BA.5.1	GR	(NSP5_P132H,Spike_H69del,NSP3_G489S,Spike_L24del,NSP4_T327I,Spike_S373P,Spike_N969K,Spike_H655Y,N_R203K,Spike_V213G,Spike_G142D,Spike_A27S,Spike_Q954H,N_P13L,Spike_P25del,N_R32del,NS3_T223I,Spike_T19I,M_Q19E,Spike_N440K,NSP4_T492I,M_D3N,Spike_N679K,Spike_N764K,NSP6_G107del,N_G204R,N_S413R,M_A63T,Spike_S371F,Spike_K417N,NSP13_R392C,Spike_T376A,NSP6_S106del,Spike_R408S,Spike_P681H,NSP4_L264F,NSP3_T24I,N_S33del,NSP1_S135R,Spike_S375F,Spike_D405N,N_E31del,NSP15_T112I,NSP6_F108del,Spike_V70del,Spike_P26del,NSP12_P323L,Spike_D614G,Spike_L452R)
EPI_ISL_13479613	16/06/2022	BA.2	BA.2	GRA	(NSP5_P132H,NSP3_G489S,Spike_L24del,NSP4_T327I,Spike_S373P,Spike_Q493R,Spike_N969K,Spike_H655Y,N_R203K,Spike_V213G,Spike_G142D,Spike_A27S,Spike_Q954H,N_P13L,Spike_P25del,N_R32del,NS3_T223I,Spike_T19I,M_Q19E,Spike_N440K,NSP4_T492I,NS8_E59stop,Spike_N679K,Spike_N764K,NSP6_G107del,N_G204R,Spike_T478K,N_S413R,M_A63T,Spike_S371F,Spike_K417N,NSP13_R392C,Spike_T376A,NSP6_S106del,Spike_R408S,NSP4_L438F,Spike_P681H,NSP4_L264F,NSP3_T24I,N_S33del,NSP1_S135R,Spike_S375F,Spike_D405N,Spike_S477N,N_E31del,NSP15_T112I,NSP6_F108del,Spike_E484A,Spike_P26del,NSP12_P323L,Spike_D614G)
EPI_ISL_13479614	16/06/2022	BA.4	BA.4	GRA	(NSP5_P132H,Spike_H69del,Spike_N658S,NSP3_G489S,Spike_L24del,NSP4_T327I,Spike_S373P,Spike_N969K,Spike_H655Y,N_R203K,Spike_V213G,Spike_G142D,Spike_A27S,Spike_Q954H,N_P13L,Spike_P25del,N_R32del,NS3_T223I,Spike_T19I,M_Q19E,NSP4_T492I,Spike_N679K,Spike_N764K,NSP6_G107del,NSP2_T573I,N_G204R,Spike_T478K,N_S413R,M_A63T,Spike_S371F,NS7b_L11F,N_P151S,Spike_K417N,NSP13_R392C,Spike_F486V,Spike_T376A,NSP6_S106del,Spike_G339D,Spike_R408S,NSP4_L264F,Spike_P681H,NSP3_T24I,N_S33del,NSP1_S135R,Spike_S375F,Spike_D405N,NSP1_F143del,Spike_S477N,NSP1_K141del,N_E31del,NSP1_S142del,NSP15_T112I,NSP6_F108del,Spike_E484A,Spike_V70del,Spike_P26del,NSP12_P323L,Spike_D614G,Spike_L452R)
EPI_ISL_13479615	16/06/2022	BA.4	BA.4.1	GRA	(NSP5_P132H,Spike_H69del,Spike_V3G,NSP3_G489S,Spike_L24del,NSP4_T327I,Spike_S373P,Spike_N969K,Spike_H655Y,N_R203K,Spike_V213G,Spike_G142D,Spike_A27S,Spike_Q954H,N_P13L,Spike_P25del,N_R32del,NS3_T223I,Spike_T19I,M_Q19E,Spike_N440K,NSP4_T492I,Spike_N679K,Spike_N764K,NSP6_G107del,N_G204R,Spike_T478K,N_S413R,M_A63T,Spike_S371F,NS7b_L11F,N_P151S,Spike_K417N,NSP13_R392C,Spike_F486V,Spike_T376A,NSP6_S106del,Spike_R408S,NSP4_L264F,Spike_P681H,NSP3_T24I,N_S33del,NSP1_S135R,Spike_S375F,Spike_D405N,NSP1_F143del,Spike_S477N,NSP1_K141del,N_E31del,NSP1_S142del,NSP15_T112I,NSP6_F108del,Spike_E484A,E_T9I,Spike_V70del,Spike_P26del,NSP12_P323L,Spike_D614G,Spike_L452R)
EPI_ISL_13479617	16/06/2022	BA.5	BA.5.1	GRA	(NSP5_P132H,Spike_H69del,NSP3_G489S,Spike_L24del,NSP4_T327I,Spike_S373P,Spike_N969K,Spike_H655Y,N_R203K,Spike_V213G,Spike_G142D,Spike_A27S,Spike_Q954H,N_P13L,Spike_P25del,N_R32del,NS3_T223I,Spike_T19I,M_Q19E,Spike_N440K,NSP4_T492I,M_D3N,Spike_N679K,Spike_N764K,NSP6_G107del,N_G204R,Spike_T478K,N_S413R,M_A63T,Spike_S371F,Spike_K417N,NSP13_R392C,Spike_F486V,Spike_T376A,NSP6_S106del,Spike_R408S,Spike_P681H,NSP4_L264F,NSP3_T24I,N_S33del,NSP1_S135R,Spike_S375F,Spike_D405N,Spike_S477N,N_E31del,NSP15_T112I,NSP6_F108del,Spike_E484A,Spike_V70del,Spike_P26del,NSP12_P323L,Spike_D614G,Spike_L452R)
EPI_ISL_13479618	16/06/2022	BA.5	BA.5.1	GRA	(NSP5_P132H,Spike_H69del,NSP3_G489S,Spike_L24del,NSP4_T327I,Spike_S373P,Spike_N969K,Spike_H655Y,N_R203K,Spike_V213G,Spike_G142D,Spike_A27S,Spike_Q954H,N_P13L,Spike_P25del,N_R32del,NS3_T223I,Spike_T19I,M_Q19E,Spike_N440K,NSP4_T492I,M_D3N,Spike_N679K,Spike_N764K,NSP6_G107del,N_G204R,Spike_T478K,N_S413R,M_A63T,Spike_S371F,Spike_K417N,NSP13_R392C,Spike_F486V,Spike_T376A,NSP6_S106del,NSP14_G17W,NSP6_K270R,Spike_L5F,Spike_R408S,Spike_P681H,NSP4_L264F,NSP3_T24I,N_S33del,NSP1_S135R,Spike_S375F,Spike_D405N,Spike_S477N,NS3_P42L,N_E31del,NSP15_T112I,NSP6_F108del,Spike_E484A,E_T9I,Spike_V70del,Spike_P26del,NSP12_P323L,Spike_D614G,Spike_L452R)
EPI_ISL_13479652	16/06/2022	BA.5	BA.5.1	GR	(NSP5_P132H,Spike_H69del,NSP3_G489S,Spike_L24del,NSP4_T327I,Spike_S373P,Spike_N969K,Spike_H655Y,N_R203K,Spike_V213G,Spike_G142D,Spike_A27S,Spike_Q954H,N_P13L,Spike_P25del,N_R32del,NS3_T223I,Spike_T19I,M_Q19E,Spike_N440K,NSP4_T492I,M_D3N,Spike_N679K,Spike_N764K,NSP6_G107del,N_G204R,N_S413R,M_A63T,Spike_S371F,Spike_K417N,NSP13_R392C,Spike_T376A,NSP6_S106del,Spike_R408S,Spike_P681H,NSP4_L264F,NSP3_T24I,N_S33del,NSP1_S135R,Spike_S375F,Spike_D405N,NSP3_T1456I,N_E31del,NSP15_T112I,NSP6_F108del,Spike_V70del,Spike_P26del,NSP12_P323L,Spike_D614G,Spike_L452R)
EPI_ISL_13479653	16/06/2022	BA.2	BA.2	GR	(NSP5_P132H,Spike_H69del,NSP1_F143L,NSP3_G489S,Spike_L24del,NSP4_T327I,Spike_S373P,Spike_N969K,Spike_H655Y,N_R203K,Spike_V213G,Spike_G142D,Spike_A27S,Spike_Q954H,N_P13L,Spike_P25del,N_R32del,NS3_T223I,Spike_T19I,M_Q19E,Spike_N440K,NSP4_T492I,Spike_N679K,Spike_N764K,NSP6_G107del,N_G204R,N_S413R,M_A63T,Spike_S371F,Spike_K417N,NSP13_R392C,Spike_T376A,NSP6_S106del,NS6_K48I,Spike_R408S,NSP4_L438F,Spike_P681H,NSP4_L264F,NSP3_T24I,N_S33del,NSP1_S135R,Spike_S375F,NSP12_I266S,Spike_D405N,N_E31del,NSP15_T112I,NSP6_F108del,Spike_V70del,Spike_Y248H,Spike_P26del,NSP12_P323L,Spike_D614G)
EPI_ISL_13479657	16/06/2022	BA.4	BA.4	GR	(NSP5_P132H,Spike_H69del,Spike_N658S,NSP3_G489S,Spike_L24del,NSP4_T327I,Spike_S373P,Spike_N969K,Spike_H655Y,N_R203K,Spike_V213G,Spike_G142D,Spike_A27S,Spike_Q954H,N_P13L,Spike_P25del,N_R32del,NS3_T223I,Spike_T19I,M_Q19E,Spike_N440K,NSP4_T492I,Spike_N679K,Spike_N764K,NSP6_G107del,N_G204R,N_S413R,M_A63T,Spike_S371F,NS7b_L11F,N_P151S,Spike_K417N,NSP13_R392C,Spike_T376A,NSP6_S106del,Spike_R408S,Spike_P681H,NSP4_L264F,Spike_V1228L,NSP3_T24I,N_S33del,NSP1_S135R,Spike_S375F,Spike_D405N,NSP1_F143del,NSP1_K141del,N_E31del,NSP1_S142del,NSP15_T112I,NSP6_F108del,Spike_V70del,Spike_P26del,NSP12_P323L,Spike_D614G,Spike_L452R)
EPI_ISL_13479722	16/06/2022	BA.5	BA.5.1	GR	(NSP5_P132H,Spike_H69del,NSP3_G489S,Spike_L24del,NSP4_T327I,Spike_S373P,Spike_N969K,Spike_H655Y,N_R203K,Spike_V213G,Spike_G142D,Spike_A27S,Spike_Q954H,N_P13L,NSP3_M953T,Spike_P25del,N_R32del,NS3_T223I,Spike_T19I,M_Q19E,NS8_P36S,Spike_N440K,NSP4_T492I,M_D3N,Spike_N679K,Spike_N764K,NSP6_G107del,N_G204R,N_S413R,M_A63T,Spike_S371F,Spike_K417N,NSP13_R392C,Spike_T376A,NSP6_S106del,Spike_R408S,Spike_P681H,NSP4_L264F,NSP3_T24I,N_S33del,NSP1_S135R,Spike_S375F,Spike_D405N,N_E31del,NSP15_T112I,NSP6_F108del,Spike_V70del,Spike_P26del,NSP12_P323L,Spike_D614G,Spike_L452R)

EPI_ISL_13535731	16/06/2022	BA.2	BA.2	GRA	(NSP5_P132H,NSP3_G489S,Spike_L24del,NSP4_T327I,Spike_S373P,Spike_H655Y,N_R203K,Spike_V213G,Spike_G142D,Spike_A27S,Spike_Q954H,N_P13L,Spike_P25del,N_R32del,NS3_T223I,Spike_T19I,M_Q19E,Spike_N440K,NSP4_T492I,Spike_N679K,Spike_N764K,NSP6_G107del,N_G204R,Spike_T478K,N_S413R,M_A63T,Spike_S371F,Spike_K417N,NSP13_R392C,Spike_T376A,NSP6_S106del,Spike_R408S,NSP4_L438F,Spike_P681H,NSP4_L264F,NSP3_T24I,N_S33del,NSP1_S135R,Spike_S375F,Spike_D405N,Spike_S247N,NSP2_R27C,Spike_S477N,N_E31del,NSP6_F108del,Spike_E484A,E_T9I,Spike_P26del,NSP12_P323L,Spike_D614G)
EPI_ISL_13535733	16/06/2022	BA.2	BA.2.9	GR	(NSP5_P132H,NSP3_G489S,Spike_L24del,NSP4_T327I,Spike_S373P,Spike_H655Y,N_R203K,Spike_V213G,Spike_G142D,Spike_A27S,Spike_Q954H,N_P13L,Spike_P25del,N_R32del,NS3_T223I,Spike_T19I,M_Q19E,Spike_N440K,NSP4_T492I,Spike_N679K,Spike_N764K,NSP6_G107del,N_G204R,N_S413R,M_A63T,Spike_S371F,NSP15_D219Y,NS3_H78Y,Spike_K417N,NSP13_R392C,Spike_T376A,NSP6_S106del,NSP6_T181I,Spike_R408S,NSP4_L438F,NSP4_L264F,Spike_P681H,NSP3_T24I,N_S33del,NSP1_S135R,Spike_S375F,Spike_D405N,N_E31del,NSP15_T112I,NSP6_F108del,E_T9I,Spike_P26del,NSP12_P323L,Spike_D614G,NSP6_V149I)
EPI_ISL_13535737	16/06/2022	BA.2	BA.2.9	GRA	(NSP5_P132H,N_T135I,NSP2_L462F,NSP3_G489S,Spike_L24del,NSP4_T327I,NSP3_P1103L,Spike_S373P,Spike_N969K,Spike_H655Y,N_R203K,Spike_V213G,Spike_G142D,Spike_A27S,Spike_Q954H,N_P13L,Spike_P25del,N_R32del,NS3_T223I,Spike_T19I,M_Q19E,Spike_N440K,NSP4_T492I,NSP13_S38P,Spike_N679K,Spike_N764K,NSP6_G107del,N_G204R,Spike_T478K,N_S413R,M_A63T,NSP2_E467G,Spike_S371F,NS8_Q27stop,NS3_H78Y,Spike_K417N,NSP13_R392C,Spike_T376A,NSP6_S106del,Spike_R408S,NSP4_L438F,NSP4_L264F,Spike_P681H,NSP3_T24I,N_S33del,NSP1_S135R,Spike_S375F,Spike_D405N,Spike_S477N,N_E31del,NSP6_F108del,Spike_E484A,E_T9I,Spike_P26del,NSP12_P323L,Spike_D614G)
EPI_ISL_13535738	16/06/2022	BA.2	BA.2	GRA	(NSP5_P132H,NSP3_G489S,Spike_L24del,NSP4_T327I,Spike_S373P,Spike_H655Y,N_R203K,Spike_V213G,Spike_G142D,Spike_A27S,Spike_Q954H,N_P13L,Spike_N501Y,Spike_P25del,N_R32del,NS3_T223I,Spike_T19I,M_Q19E,Spike_N440K,NSP4_T492I,Spike_N679K,Spike_N764K,NSP6_G107del,Spike_D796Y,N_G204R,Spike_T478K,N_S413R,M_A63T,NS3_I37T,Spike_S371F,Spike_K417N,NSP13_R392C,Spike_T376A,NSP6_S106del,Spike_R408S,NSP4_L438F,Spike_P681H,NSP4_L264F,NSP3_T24I,N_S33del,NSP1_S135R,Spike_S375F,Spike_D405N,Spike_S477N,N_E31del,NSP6_F108del,Spike_E484A,E_T9I,Spike_P26del,NSP12_P323L,Spike_D614G)
EPI_ISL_13535740	16/06/2022	BA.2	BA.2	GRA	(NSP5_P132H,N_T296I,NSP3_G489S,Spike_L24del,NSP4_T327I,Spike_S373P,Spike_H655Y,N_R203K,Spike_V213G,Spike_G142D,Spike_A27S,Spike_Q954H,N_P13L,NSP3_T976K,Spike_P25del,N_R32del,NS3_T223I,Spike_T19I,M_Q19E,Spike_N440K,NSP4_T492I,Spike_N679K,Spike_N764K,NSP6_G107del,NSP3_T725I,N_G204R,Spike_T478K,N_S413R,M_A63T,NSP6_L37F,Spike_R346T,Spike_S371F,NS6_M58I,Spike_K417N,NSP13_R392C,Spike_T376A,NSP6_S106del,Spike_R408S,NSP4_L438F,Spike_P681H,NSP4_L264F,NSP3_T24I,N_S33del,NSP1_S135R,Spike_S375F,Spike_D405N,Spike_S477N,N_E31del,NSP6_F108del,Spike_E484A,E_T9I,Spike_P26del,NSP12_P323L,Spike_D614G)
EPI_ISL_13479605	17/06/2022	BA.4	BA.4	GRA	(NSP5_P132H,Spike_H69del,Spike_N658S,NSP3_A264V,NSP3_G489S,Spike_L24del,NSP4_T327I,Spike_S373P,Spike_N969K,Spike_H655Y,N_R203K,Spike_V213G,Spike_G142D,Spike_A27S,Spike_Q954H,N_P13L,Spike_P25del,N_R32del,NS3_T223I,Spike_T19I,M_Q19E,Spike_N440K,NSP4_T492I,Spike_N679K,Spike_N764K,NSP6_G107del,N_G204R,Spike_T478K,N_S413R,M_A63T,Spike_S371F,NS7b_L11F,NSP10_T12I,N_P151S,Spike_K417N,NSP13_R392C,Spike_F486V,Spike_T376A,NSP6_S106del,Spike_R408S,NSP4_L264F,Spike_P681H,NSP3_T24I,N_S33del,NSP1_S135R,Spike_S375F,Spike_D405N,NSP1_F143del,Spike_S477N,NSP1_K141del,N_E31del,NSP1_S142del,NSP15_T112I,NSP6_F108del,Spike_E484A,Spike_V70del,Spike_P26del,NSP12_P323L,Spike_D614G,Spike_L452R)
EPI_ISL_13479608	17/06/2022	BA.4	BA.4	GR	(NSP5_P132H,Spike_H69del,Spike_N658S,NSP3_G489S,Spike_L24del,NSP4_T327I,Spike_S373P,Spike_N969K,Spike_H655Y,N_R203K,Spike_V213G,Spike_G142D,Spike_A27S,Spike_Q954H,N_P13L,Spike_P25del,N_R32del,NS3_T223I,Spike_T19I,M_Q19E,Spike_N440K,NSP4_T492I,Spike_N679K,Spike_N764K,NSP6_G107del,N_G204R,N_S413R,M_A63T,Spike_S371F,NS7b_L11F,N_P151S,Spike_K417N,NSP13_R392C,Spike_T376A,NSP6_S106del,Spike_R408S,Spike_P681H,NSP4_L264F,NSP3_T24I,N_S33del,NSP1_S135R,Spike_S375F,Spike_D405N,NSP1_F143del,NSP1_K141del,N_E31del,NSP1_S142del,NSP15_T112I,NSP6_F108del,Spike_V70del,Spike_P26del,NSP12_P323L,Spike_D614G,Spike_L452R)
EPI_ISL_13479609	17/06/2022	BA.5	BA.5.1	GRA	(NSP5_P132H,Spike_H69del,NSP3_G489S,Spike_L24del,NSP4_T327I,Spike_S373P,Spike_N969K,Spike_H655Y,N_R203K,Spike_V213G,Spike_G142D,Spike_A27S,Spike_Q954H,N_P13L,Spike_P25del,N_R32del,NS3_T223I,Spike_T19I,M_Q19E,Spike_N440K,NSP4_T492I,M_D3N,Spike_N679K,Spike_N764K,NSP6_G107del,N_G204R,Spike_T478K,N_S413R,NSP9_T21I,M_A63T,Spike_S371F,Spike_K417N,NSP13_R392C,Spike_F486V,Spike_T376A,NSP6_S106del,Spike_R408S,Spike_P681H,NSP4_L264F,NSP3_T24I,N_S33del,NSP1_S135R,Spike_S375F,Spike_D405N,Spike_S477N,N_E31del,NSP15_T112I,NSP6_F108del,Spike_E484A,E_T9I,Spike_V70del,Spike_P26del,NSP12_P323L,Spike_D614G,Spike_L452R)
EPI_ISL_13479642	17/06/2022	BA.4	BA.4	GR	(NSP5_P132H,Spike_H69del,Spike_N658S,NSP3_G489S,Spike_L24del,NSP4_T327I,Spike_S373P,Spike_N969K,Spike_H655Y,N_R203K,Spike_V213G,Spike_G142D,Spike_A27S,Spike_Q954H,N_P13L,Spike_P25del,N_R32del,NS3_T223I,Spike_T19I,M_Q19E,Spike_N440K,NSP4_T492I,Spike_N679K,Spike_N764K,NSP6_G107del,N_G204R,N_S413R,M_A63T,Spike_S371F,NS7b_L11F,N_P151S,Spike_K417N,NSP13_R392C,Spike_T376A,NSP6_S106del,Spike_R408S,Spike_P681H,NSP4_L264F,NSP3_T24I,N_S33del,NSP1_S135R,Spike_S375F,Spike_D405N,NSP1_F143del,NSP1_K141del,N_E31del,NSP1_S142del,NSP15_T112I,NSP6_F108del,E_T9I,Spike_V70del,Spike_P26del,NSP12_P323L,Spike_D614G,Spike_L452R)
EPI_ISL_13479644	17/06/2022	BA.5	BA.5.1	GRA	(NSP5_P132H,Spike_H69del,NSP3_G489S,Spike_L24del,NSP4_T327I,Spike_S373P,Spike_N969K,Spike_H655Y,N_R203K,Spike_V213G,Spike_G142D,Spike_A27S,Spike_Q954H,N_P13L,Spike_P25del,N_R32del,NS3_T223I,Spike_T19I,M_Q19E,Spike_N440K,NSP4_T492I,M_D3N,Spike_N679K,Spike_N764K,NSP6_G107del,N_G204R,Spike_T478K,N_S413R,M_A63T,Spike_S371F,M_L90V,Spike_K417N,NSP13_R392C,Spike_F486V,Spike_T376A,NSP6_S106del,NSP14_G17W,NSP6_K270R,Spike_G339D,Spike_R408S,NSP4_L264F,Spike_P681H,NSP3_T24I,N_S33del,NSP1_S135R,Spike_S375F,Spike_D405N,Spike_S477N,N_E31del,NSP15_T112I,NSP6_F108del,Spike_E484A,E_T9I,Spike_V70del,Spike_P26del,NSP12_P323L,Spike_D614G,Spike_L452R)
EPI_ISL_13479645	17/06/2022	BA.2	BA.2	GRA	(NSP5_P132H,Spike_H69del,Spike_N658S,NSP3_G489S,Spike_L24del,NSP4_T327I,Spike_K964R,Spike_S373P,Spike_N969K,Spike_H655Y,N_R203K,Spike_V213G,Spike_G142D,Spike_A27S,Spike_Q954H,N_P13L,Spike_P25del,N_R32del,NS3_T223I,Spike_T19I,M_Q19E,Spike_N440K,NSP4_T492I,Spike_N679K,Spike_N764K,NSP6_G107del,N_G204R,Spike_T478K,N_S413R,M_A63T,Spike_S371F,NS7b_L11F,Spike_K417N,NSP13_R392C,Spike_F486V,Spike_T376A,NSP6_S106del,Spike_G339D,Spike_R408S,NSP4_L264F,Spike_P681H,N_S33del,NSP1_S135R,Spike_S375F,Spike_D405N,NSP1_F143del,Spike_S477N,NSP1_K141del,N_E31del,NSP1_S142del,NSP15_T112I,NSP6_F108del,Spike_E484A,Spike_V70del,Spike_P26del,NSP12_P323L,Spike_L452R)

EPI_ISL_13535727	17/06/2022	BA.2	BA.2	GR	(NSP5_P132H,NSP3_G489S,Spike_L24del,NSP4_T327I,Spike_S373P,Spike_N969K,Spike_H655Y,N_R203K,NSP15_S25A,Spike_V213G,Spike_G142D,Spike_A27S,Spike_Q954H,N_P13L,Spike_P25del,N_R32del,NS3_T223I,Spike_T19I,M_Q19E,Spike_N440K,NSP4_T492I,Spike_N679K,Spike_N764K,NSP6_G107del,N_G204R,N_S413R,M_A63T,Spike_S371F,Spike_K417N,NSP13_R392C,Spike_T376A,NSP6_S106del,NSP3_P1921S,Spike_R408S,NSP4_L438F,Spike_P681H,NSP4_L264F,NSP3_T24I,N_S33del,NSP1_S135R,Spike_S375F,Spike_D405N,N_E31del,NSP6_F108del,E_T9I,Spike_P26del,NSP12_P323L,Spike_D614G)
EPI_ISL_13535783	17/06/2022	BA.5	BA.5.3.1	GRA	(NSP5_P132H,Spike_H69del,NSP3_G489S,Spike_L24del,NSP4_T327I,Spike_S373P,Spike_N969K,Spike_H655Y,N_R203K,Spike_V213G,Spike_G142D,Spike_A27S,Spike_Q954H,N_P13L,Spike_P25del,N_R32del,NS3_T223I,Spike_T19I,M_Q19E,Spike_N440K,NSP4_T492I,N_E136D,M_D3N,Spike_N679K,Spike_N764K,NSP6_G107del,N_G204R,Spike_T478K,N_S413R,M_A63T,NSP2_Q376K,Spike_S371F,Spike_K417N,NSP13_R392C,Spike_F486V,Spike_T376A,NSP6_S106del,Spike_R408S,Spike_P681H,NSP4_L264F,NSP3_T24I,N_S33del,NSP1_S135R,Spike_S375F,Spike_D405N,Spike_S477N,NS3_P240L,N_E31del,NSP15_T112I,NSP6_F108del,Spike_E484A,NS3_K21N,E_T9I,Spike_V70del,Spike_P26del,NSP12_P323L,Spike_D614G,Spike_L452R)
EPI_ISL_13535790	17/06/2022	BA.5	BA.5.3.1	GR	(NSP5_P132H,Spike_H69del,NSP3_G489S,Spike_L24del,NSP4_T327I,Spike_S373P,Spike_N969K,Spike_H655Y,N_R203K,Spike_V213G,Spike_G142D,Spike_A27S,Spike_Q954H,N_P13L,Spike_P25del,N_R32del,NS3_T223I,Spike_T19I,M_Q19E,Spike_N440K,NSP4_T492I,N_E136D,M_D3N,Spike_N679K,Spike_N764K,NSP6_G107del,N_G204R,N_S413R,M_A63T,NSP2_Q376K,Spike_S371F,Spike_K417N,NSP13_R392C,Spike_T376A,NSP6_S106del,Spike_R408S,Spike_P681H,NSP4_L264F,NSP3_T24I,N_S33del,NSP1_S135R,Spike_S375F,Spike_D405N,N_E31del,NSP15_T112I,NSP6_F108del,E_T9I,Spike_V70del,Spike_P26del,NSP12_P323L,Spike_D614G,Spike_L452R)
EPI_ISL_13479602	18/06/2022	BA.2	BA.2.9.2	GR	(NSP5_P132H,NSP4_N203S,NSP3_G489S,Spike_L24del,NSP4_T327I,Spike_S373P,Spike_N969K,Spike_H655Y,N_R203K,Spike_V213G,Spike_G142D,Spike_A27S,Spike_Q954H,N_P13L,Spike_P25del,N_R32del,NS3_T223I,Spike_T19I,M_Q19E,Spike_N440K,NSP4_T492I,Spike_N679K,Spike_N764K,NSP6_G107del,N_G204R,N_S413R,M_A63T,Spike_S371F,NS3_H78Y,Spike_K417N,NSP13_R392C,Spike_T376A,NSP6_S106del,Spike_R408S,NSP4_L438F,Spike_P681H,NSP4_L264F,NSP3_T24I,N_S33del,NSP1_S135R,Spike_S375F,Spike_D405N,NSP3_T1189I,N_E31del,NSP15_T112I,NSP6_F108del,Spike_P26del,NSP12_P323L,Spike_D614G)
EPI_ISL_13479603	18/06/2022	BA.5	BA.5.1	GRA	(NSP5_P132H,Spike_H69del,NSP3_G489S,Spike_L24del,NSP4_T327I,Spike_S373P,Spike_N969K,Spike_H655Y,N_R203K,Spike_V213G,Spike_G142D,Spike_A27S,Spike_Q954H,N_P13L,Spike_P25del,N_R32del,NS3_T223I,Spike_T19I,M_Q19E,Spike_N440K,NSP4_T492I,M_D3N,Spike_N679K,Spike_N764K,NSP6_G107del,N_G204R,Spike_T478K,N_S413R,M_A63T,Spike_S371F,Spike_K417N,NSP13_R392C,Spike_F486V,Spike_T376A,NSP6_S106del,Spike_R408S,Spike_P681H,NSP4_L264F,NSP3_T24I,N_S33del,NSP1_S135R,Spike_S375F,Spike_D405N,Spike_S477N,N_E31del,NSP15_T112I,NSP6_F108del,Spike_E484A,E_T9I,Spike_V70del,Spike_P26del,NSP12_P323L,Spike_D614G,Spike_L452R)
EPI_ISL_13479604	18/06/2022	BA.2	BA.2.12.1	GRA	(NSP5_P132H,NSP3_G489S,Spike_L24del,NSP4_T327I,Spike_S373P,Spike_N969K,Spike_H655Y,N_R203K,Spike_V213G,Spike_G142D,Spike_A27S,Spike_Q954H,N_P13L,Spike_P25del,N_R32del,NS3_T223I,Spike_T19I,M_Q19E,Spike_N440K,NSP4_T492I,Spike_N679K,Spike_N764K,NSP6_G107del,N_G204R,Spike_T478K,N_S413R,M_A63T,Spike_S371F,NS7a_V29I,Spike_K417N,NSP13_R392C,Spike_T376A,NSP6_S106del,Spike_R408S,NSP4_L438F,Spike_P681H,NSP4_L264F,NSP3_T24I,N_S33del,NSP1_S135R,Spike_S375F,Spike_D405N,Spike_S477N,N_E31del,NSP15_T112I,NSP6_F108del,Spike_E484A,E_T9I,Spike_P26del,NSP12_P323L,Spike_D614G,Spike_L452Q)
EPI_ISL_13479607	18/06/2022	BA.4	BA.4	GRA	(NSP5_P132H,Spike_H69del,Spike_N658S,NSP3_G489S,Spike_L24del,NSP4_T327I,Spike_S373P,Spike_N969K,Spike_H655Y,N_R203K,Spike_V213G,Spike_G142D,Spike_A27S,Spike_Q954H,N_P13L,Spike_P25del,N_R32del,NS3_T223I,Spike_T19I,M_Q19E,Spike_N440K,NSP4_T492I,Spike_N679K,Spike_N764K,NSP6_G107del,N_G204R,Spike_T478K,N_S413R,M_A63T,Spike_S371F,NS7b_L11F,N_P151S,Spike_K417N,NSP13_R392C,Spike_F486V,Spike_T376A,NSP6_S106del,Spike_R408S,NSP4_L264F,Spike_P681H,NSP3_T24I,N_S33del,NSP1_S135R,Spike_S375F,Spike_D405N,NSP1_F143del,Spike_S477N,NSP1_K141del,N_E31del,NSP1_S142del,NSP15_T112I,NSP6_F108del,Spike_E484A,Spike_V70del,Spike_P26del,NSP12_P323L,Spike_D614G,Spike_L452R)
EPI_ISL_13479724	18/06/2022	BA.5	BA.5.1	GRA	(NSP5_P132H,Spike_H69del,NSP3_G489S,Spike_L24del,NSP4_T327I,Spike_S373P,Spike_N969K,Spike_H655Y,N_R203K,Spike_V213G,Spike_G142D,Spike_A27S,Spike_Q954H,N_P13L,Spike_P25del,N_R32del,NS3_T223I,Spike_T19I,M_Q19E,Spike_N440K,NSP4_T492I,M_D3N,Spike_N679K,Spike_N764K,NSP6_G107del,N_G204R,Spike_T478K,N_S413R,M_A63T,Spike_S371F,Spike_K417N,NSP13_R392C,Spike_F486V,Spike_T376A,NSP6_S106del,NSP14_G17W,NSP6_K270R,Spike_R408S,Spike_P681H,NSP4_L264F,NSP3_T24I,N_S33del,NSP1_S135R,Spike_S375F,Spike_D405N,Spike_S477N,N_E31del,NSP15_T112I,NSP6_F108del,Spike_E484A,E_T9I,Spike_V70del,Spike_P26del,NSP12_P323L,Spike_D614G,Spike_L452R)
EPI_ISL_13479725	18/06/2022	BA.5	BA.5.1	GR	(NSP5_P132H,Spike_H69del,NSP3_G489S,Spike_L24del,NSP4_T327I,Spike_S373P,Spike_N969K,Spike_H655Y,N_R203K,Spike_V213G,Spike_G142D,Spike_A27S,Spike_Q954H,N_P13L,Spike_P25del,N_R32del,NS3_T223I,Spike_T19I,M_Q19E,Spike_N440K,NSP4_T492I,M_D3N,Spike_N679K,Spike_N764K,NSP6_G107del,N_G204R,N_S413R,M_A63T,Spike_S371F,Spike_K417N,NSP13_R392C,Spike_T376A,NSP6_S106del,Spike_L5F,Spike_R408S,Spike_P681H,NSP4_L264F,NSP3_T24I,N_S33del,NSP1_S135R,Spike_S375F,Spike_D405N,N_E31del,NSP15_T112I,NSP6_F108del,Spike_V70del,Spike_P26del,NSP12_P323L,Spike_D614G,Spike_L452R)
EPI_ISL_13535777	18/06/2022	BA.5	BA.5.2	GRA	(NSP5_P132H,Spike_H69del,NSP3_G489S,Spike_L24del,NSP4_T327I,Spike_S373P,Spike_N969K,Spike_H655Y,N_R203K,Spike_V213G,Spike_G142D,Spike_A27S,Spike_Q954H,N_P13L,Spike_P25del,N_R32del,NS3_T223I,Spike_T19I,M_Q19E,Spike_N440K,NSP4_T492I,M_D3N,Spike_N679K,Spike_N764K,NSP6_G107del,N_G204R,Spike_T478K,N_S413R,M_A63T,Spike_S371F,Spike_K417N,NSP13_R392C,Spike_F486V,Spike_T376A,NSP6_S106del,NSP13_T127N,NS3_L108F,Spike_R408S,Spike_P681H,NSP4_L264F,NSP3_T24I,N_S33del,NSP1_S135R,Spike_S375F,Spike_D405N,Spike_S477N,N_E31del,NSP15_T112I,NSP6_F108del,Spike_E484A,E_T9I,Spike_V70del,Spike_P26del,NSP12_P323L,Spike_D614G,Spike_L452R)
EPI_ISL_13479646	19/06/2022	BA.5	BA.5.1	GRA	(NSP5_P132H,Spike_H69del,NSP3_G489S,Spike_L24del,NSP4_T327I,Spike_S373P,Spike_N969K,Spike_H655Y,NS6_D6G,N_R203K,Spike_V213G,Spike_G142D,Spike_A27S,Spike_Q954H,N_P13L,Spike_P25del,N_R32del,NS3_T223I,Spike_T19I,M_Q19E,Spike_N440K,NSP4_T492I,M_D3N,Spike_N679K,Spike_N764K,NSP6_G107del,N_G204R,Spike_T478K,N_S413R,M_A63T,Spike_S371F,Spike_K417N,NSP13_R392C,Spike_F486V,Spike_T376A,NSP6_S106del,Spike_R408S,Spike_P681H,NSP4_L264F,NSP3_T24I,N_S33del,NSP1_S135R,Spike_S375F,Spike_D405N,Spike_S477N,N_E31del,NSP15_T112I,NSP6_F108del,Spike_E484A,E_T9I,Spike_V70del,Spike_P26del,NSP12_P323L,Spike_D614G,Spike_L452R)
EPI_ISL_13479647	19/06/2022	BA.5	BA.5.1	GRA	(NSP5_P132H,Spike_H69del,NSP3_G489S,Spike_L24del,NSP4_T327I,Spike_S373P,Spike_H655Y,N_R203K,Spike_V213G,Spike_G142D,Spike_A27S,Spike_Q954H,N_P13L,Spike_P25del,N_R32del,NS3_T223I,Spike_T19I,M_Q19E,Spike_N440K,NSP4_T492I,M_D3N,Spike_N679K,Spike_N764K,NSP6_G107del,N_G204R,Spike_T478K,N_S413R,M_A63T,Spike_S371F,Spike_K417N,NSP13_R392C,Spike_F486V,Spike_T376A,NSP6_S106del,Spike_R408S,Spike_P681H,NSP4_L264F,NSP3_T24I,N_S33del,NSP1_S135R,Spike_S375F,Spike_D405N,Spike_S477N,N_E31del,NSP15_T112I,NSP6_F108del,Spike_E484A,E_T9I,Spike_V70del,NSP13_R155L,Spike_P26del,NSP12_P323L,Spike_D614G,Spike_L452R)

EPI_ISL_13479651	19/06/2022	BA.2	BA.2	GRA	(NSP5_P132H,NSP3_G489S,Spike_L24del,NSP4_T327I,Spike_S373P,Spike_N969K,Spike_H655Y,N_R203K,Spike_V213G,Spike_G142D,Spike_A27S,Spike_Q954H,N_P13L,Spike_P25del,N_R32del,NS3_T223I,Spike_T19I,M_Q19E,Spike_N440K,NSP4_T492I,Spike_N679K,Spike_N764K,NSP6_G107del,N_G204R,Spike_T478K,N_S413R,M_A63T,Spike_S371F,NSP1_M85del,NSP1_E87K,Spike_K417N,NSP13_R392C,Spike_T376A,NSP6_S106del,Spike_R408S,NSP4_L438F,NSP4_L264F,Spike_P681H,NSP3_T24I,N_S33del,NSP1_S135R,Spike_S375F,Spike_D405N,NSP3_S126L,Spike_S477N,N_E31del,NSP1_V86del,NSP15_T112I,NSP6_F108del,Spike_E484A,E_T9I,Spike_P26del,NSP12_P323L,Spike_D614G)
EPI_ISL_13479654	19/06/2022	BA.2	BA.2.44	GRA	(NSP5_P132H,NSP4_T319A,NSP3_G489S,Spike_L24del,NSP4_T327I,Spike_S373P,Spike_N969K,Spike_H655Y,N_R203K,Spike_V213G,Spike_G142D,Spike_A27S,Spike_Q954H,N_P13L,Spike_P25del,N_R32del,NS3_T223I,Spike_T19I,M_Q19E,Spike_N440K,NSP4_T492I,Spike_N679K,Spike_N764K,NSP6_G107del,N_G204R,Spike_T478K,N_S413R,M_A63T,Spike_K417N,NSP13_R392C,Spike_T376A,NSP6_S106del,Spike_R408S,NSP4_L438F,Spike_P681H,NSP4_L264F,NSP3_T24I,N_S33del,NSP1_S135R,Spike_S375F,Spike_D405N,Spike_S477N,N_E31del,NSP15_T112I,NSP6_F108del,Spike_E484A,E_T9I,Spike_S371Y,Spike_P26del,NSP12_P323L,Spike_D614G)
EPI_ISL_13479656	19/06/2022	BA.2	BA.2	GR	(NSP5_P132H,NSP3_G489S,Spike_L24del,NSP4_T327I,Spike_S373P,Spike_N969K,Spike_H655Y,N_R203K,Spike_V213G,Spike_G142D,NSP10_L138I,Spike_A27S,NSP13_T351I,Spike_Q954H,N_P13L,Spike_P25del,N_R32del,NS3_T223I,Spike_T19I,M_Q19E,Spike_N440K,NSP4_T492I,Spike_N679K,Spike_N764K,NSP6_G107del,N_G204R,N_S413R,M_A63T,NSP4_T461I,Spike_S371F,Spike_K417N,NSP13_R392C,Spike_T376A,NSP6_S106del,Spike_R408S,Spike_P681H,NSP4_L264F,NSP3_T24I,N_S33del,NSP1_S135R,Spike_S375F,Spike_D405N,N_E31del,NSP15_T112I,NSP6_F108del,E_T9I,Spike_P26del,NSP12_P323L,Spike_D614G)
EPI_ISL_13479713	19/06/2022	BA.5	BA.5.1	GR	(NSP5_P132H,Spike_H69del,NSP3_G489S,Spike_L24del,NSP4_T327I,Spike_S373P,Spike_N969K,Spike_H655Y,N_R203K,Spike_V213G,Spike_G142D,NS3_T89I,Spike_A27S,Spike_Q954H,N_P13L,Spike_P25del,N_R32del,NS3_T223I,Spike_T19I,NSP5_S46F,M_Q19E,Spike_N440K,NSP4_T492I,M_D3N,Spike_N679K,Spike_N764K,NSP6_G107del,N_G204R,N_S413R,NSP9_T21I,M_A63T,NSP3_R1297S,Spike_S371F,Spike_K417N,NSP13_R392C,Spike_T376A,NSP6_S106del,Spike_R408S,Spike_P681H,NSP4_L264F,NSP3_T24I,N_S33del,NSP1_S135R,Spike_S375F,Spike_D405N,N_E31del,NSP15_T112I,NSP6_F108del,E_T9I,Spike_V70del,Spike_P26del,NSP12_P323L,Spike_D614G,Spike_L452R)
EPI_ISL_13535761	19/06/2022	BA.2	BA.2	GR	(NSP5_P132H,NSP3_G489S,Spike_L24del,NSP4_T327I,Spike_S373P,Spike_N969K,Spike_H655Y,N_R203K,Spike_V213G,Spike_G142D,NSP10_L138I,Spike_A27S,NSP13_T351I,Spike_Q954H,N_P13L,Spike_P25del,N_R32del,NS3_T223I,Spike_T19I,M_Q19E,Spike_N440K,NSP4_T492I,Spike_N679K,Spike_N764K,NSP6_G107del,N_G204R,N_S413R,M_A63T,NSP4_T461I,Spike_S371F,Spike_K417N,NSP13_R392C,Spike_T376A,NSP6_S106del,Spike_R408S,Spike_P681H,NSP4_L264F,NSP3_T24I,N_S33del,NSP1_S135R,Spike_S375F,Spike_D405N,N_E31del,NSP15_T112I,NSP6_F108del,E_T9I,Spike_P26del,NSP12_P323L,Spike_D614G)
EPI_ISL_13535765	19/06/2022	BA.2	BA.2	GR	(NSP5_P132H,NSP3_G489S,Spike_L24del,NSP4_T327I,Spike_S373P,Spike_H655Y,N_R203K,Spike_V213G,Spike_G142D,Spike_A27S,Spike_Q954H,N_P13L,Spike_P25del,N_R32del,NS3_T223I,Spike_T19I,M_Q19E,Spike_N440K,NSP4_T492I,Spike_N679K,Spike_N764K,NSP6_G107del,N_S193N,N_G204R,N_S413R,M_A63T,NSP16_K160R,Spike_S371F,NSP3_A1527T,Spike_K417N,NSP13_R392C,Spike_T376A,NSP6_S106del,Spike_R408S,Spike_E1144G,NSP4_L438F,Spike_P681H,NSP4_L264F,NSP3_T24I,N_S33del,NSP1_S135R,Spike_S375F,Spike_D405N,N_E31del,NSP6_F108del,NSP3_P389S,E_T9I,Spike_P26del,NSP12_P323L,Spike_D614G)
EPI_ISL_13535774	19/06/2022	BA.4	BA.4	GRA	(NSP5_P132H,Spike_H69del,NS6_E55Y,NSP3_G489S,Spike_L24del,NSP4_T327I,Spike_S373P,Spike_N969K,Spike_H655Y,N_R203K,Spike_V213G,Spike_G142D,Spike_A27S,Spike_Q954H,N_P13L,Spike_P25del,N_R32del,NS3_T223I,Spike_T19I,M_Q19E,Spike_N440K,NSP4_T492I,Spike_S640F,Spike_N679K,Spike_N764K,NSP6_G107del,Spike_D796Y,N_G204R,N_S413R,M_A63T,Spike_S371F,NS7b_L11F,N_P151S,Spike_K417N,NSP13_R392C,Spike_T376A,NSP6_S106del,Spike_R408S,NSP14_I42V,NSP4_L264F,Spike_P681H,NSP3_T24I,N_S33del,Spike_S375F,NSP1_S135R,Spike_D405N,NSP1_F143del,NSP1_K141del,N_E31del,NSP1_S142del,NSP15_T112I,NSP6_F108del,E_T9I,Spike_V70del,Spike_P26del,NSP12_P323L,Spike_D614G,Spike_L452R)
